# CRISPR-free base editors with enhanced activity and expanded targeting scope in mitochondrial and nuclear DNA

**DOI:** 10.1038/s41587-022-01256-8

**Published:** 2022-04-04

**Authors:** Beverly Y. Mok, Anna V. Kotrys, Aditya Raguram, Tony P. Huang, Vamsi K. Mootha, David R. Liu

**Affiliations:** 1 Merkin Institute of Transformative Technologies in Healthcare, Broad Institute of MIT and Harvard, Cambridge, MA USA; 2 Department of Chemistry and Chemical Biology, Harvard University, Cambridge, MA USA; 3 Howard Hughes Medical Institute, Harvard University, Cambridge, MA USA; 4 Howard Hughes Medical Institute and Department of Molecular Biology, Massachusetts General Hospital, Harvard Medical School, Boston, MA USA; 5 Broad Institute of MIT and Harvard, Cambridge, MA USA

**Keywords:** Gene targeting, Targeted gene repair

## Abstract

The all-protein cytosine base editor DdCBE uses TALE proteins and a double-stranded DNA-specific cytidine deaminase (DddA) to mediate targeted C•G-to-T•A editing. To improve editing efficiency and overcome the strict TC sequence-context constraint of DddA, we used phage-assisted non-continuous and continuous evolution to evolve DddA variants with improved activity and expanded targeting scope. Compared to canonical DdCBEs, base editors with evolved DddA6 improved mitochondrial DNA (mtDNA) editing efficiencies at TC by 3.3-fold on average. DdCBEs containing evolved DddA11 offered a broadened HC (H = A, C or T) sequence compatibility for both mitochondrial and nuclear base editing, increasing average editing efficiencies at AC and CC targets from less than 10% for canonical DdCBE to 15–30% and up to 50% in cell populations sorted to express both halves of DdCBE. We used these evolved DdCBEs to efficiently install disease-associated mtDNA mutations in human cells at non-TC target sites. DddA6 and DddA11 substantially increase the effectiveness and applicability of all-protein base editing.

## Abstract (web summary)

Continuously evolved double-stranded DNA deaminases increase the efficiency and targeting scope of all-protein base editors.

## Main

Each human cell can contain several hundred copies of circular mtDNA that encodes RNAs and proteins that mediate ATP production^1–3^. Owing to the essential role of the mitochondria in energy homeostasis, single-nucleotide mutations in the mtDNA can contribute to developmental disorders, neuromuscular disease, cancer progression and a growing number of other human diseases^4–7^. Technologies that enable the precise installation of point mutations within mtDNA could reveal the role of these mutations in pathogenesis and provide ways to correct them for potential therapeutic applications.

Programmable nucleases can make targeted double-strand breaks within mtDNA copies that contain specific mutations, resulting in the elimination of those copies^8–12^. Nucleases, however, cannot introduce specified sequence changes. Genome editing agents, including base editors^13,14^ and prime editors^15^, directly install precise changes in a target DNA sequence but typically rely on a guide RNA sequence to direct CRISPR–Cas proteins for binding to its target DNA. Owing to the challenge of importing guide RNAs into the mitochondria, CRISPR-based systems have, thus far, not been used reliably for mtDNA engineering^16,17^.

To begin to address this challenge, we recently developed DdCBE to enable targeted C•G-to-T•A conversions within mtDNA^18^. DdCBE uses two TALE proteins to specify the double-stranded DNA (dsDNA) region for editing. Each TALE is fused to a non-toxic half of DddA and one copy of uracil glycosylase inhibitor (UGI) protein. Binding of two TALE fused split-DddA–UGI fusions to adjacent sites promotes reassembly of functional DddA for deamination of target cytosines within the dsDNA spacing region. DdCBEs have been applied for mitochondrial base editing in human embryo, mice, zebrafish and plants^19–24^.

In our initial studies, we observed a range of mtDNA editing efficiencies (4.6–49%) depending on the position of the target C within the spacing region between the DNA-bound DdCBE halves^18^. We hypothesized that enhancing the activity of split DddA could increase mtDNA editing efficiencies at putative 5′-TC contexts by improving the compatibility of DddA with different TALE designs and deaminase orientations.

Given the strict sequence preference of DddA, our initial DdCBE is limited predominantly to TC targets. In this study, we sought to increase DdCBE activity at both TC and non-TC targets by applying rapid phage-assisted continuous evolution (PACE) and related phage-assisted non-continuous evolution (PANCE) methods^25,26^. Development of a selection circuit for DdCBE activity followed by PANCE and PACE resulted in several DddA variants with conserved mutations enriched during evolution. Evolved variants DddA6 and DddA11 mediated ~4.3-fold average improvement in mtDNA base editing efficiency at TC targets compared to wild-type DddA. Notably, DddA11 increased average bulk editing levels at AC and CC targets in the mtDNA and nucleus from less than 10% with canonical DdCBE to ~15–30%. These variants collectively enable the installation or correction of C•G-to-T•A point mutations at both TC and non-TC targets, substantially expanding the overall utility of all-protein DdCBEs.

## Results

### Adapting BE-PACE to evolve TALE-based DdCBEs

**Fig. 1:**
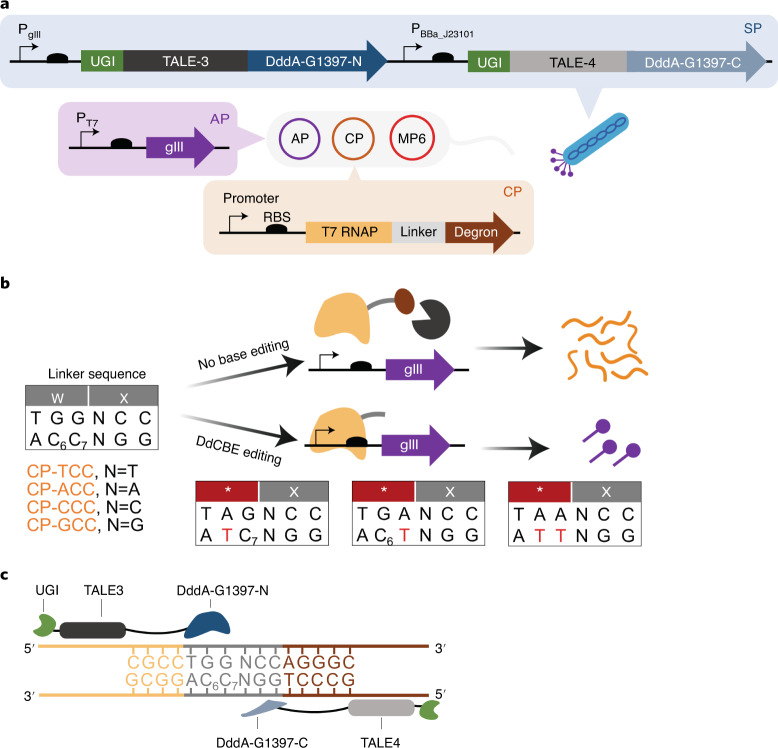
Phage-assisted evolution of DddA-derived cytosine base editor for improved activity and expanded targeting scope. **a**, Selection to evolve DdCBE using PANCE and PACE. An AP (purple) contains gIII driven by the T7 promoter. The CP (orange) expresses a T7 RNAP–degron fusion. The evolving T7-DdCBE containing DddA split at G1397 is encoded in the SP (blue). Where relevant, the promoters are indicated. **b**, A 2-amino-acid linker connects T7 RNAP to the degron. The linker sequence contains cytidines C_6_ and C_7_ that are targets for DdCBE editing. The nucleotide at position 8 can be varied to T, A, C or G to form plasmids CP-TCC, CP-ACC, CP-CCC and CP-GCC, respectively. In the absence of target C-to-T editing, expression of degron (brown) results in proteolysis of T7 RNAP (orange) and inhibition of gIII expression. Active T7-DdCBE edits one or both target cytidines to install a stop codon (*) within the linker, thus restoring active T7 RNAP to mediate gIII expression. **c**, Architecture of T7-DdCBE and the 15-bp target spacing region. Nucleotides corresponding to DNA sequences within T7 RNAP, linker and degron genes are colored in orange, gray and brown, respectively.

PACE uses an M13 phage that is modified to contain an evolving gene in place of gene III (gIII)^27^. gIII encodes a capsid protein pIII that is essential for producing infectious phage progeny. To establish a selection circuit, gIII is encoded in an accessory plasmid (AP) within the *Escherichia coli* host cell such that gIII expression is dependent on the evolving activity. We previously reported a BE-PACE system to evolve CRISPR cytosine base editors^25^. In this system, the AP encodes gIII under the control of a T7 promoter. A complementary plasmid (CP) encodes T7 RNA polymerase (T7 RNAP) fused to a degron through a 2-amino-acid linker (Fig. 1a). In the absence of C•G-to-T•A editing of the linker sequence, the degron triggers constitutive proteolysis of T7 RNAP, preventing gIII expression (Fig. 1b). The target cytosines for DdCBE-mediated editing in this selection are C_6_ and C_7_, where the subscripted numbers refer to their positions in the spacing region, counting the DNA nucleotide immediately after the binding site of the left-side TALE (TALE3) as position 1 (Fig. 1c). Successful C•G-to-T•A editing of either or both C_6_ and C_7_ targets introduces a stop codon within the linker to prevent translation of the degron tag. Active T7 RNAP then initiates gIII expression (Fig. 1b). The nucleotide at position 8 may be modified to A, T, C or G to enable selection against TC and non-TC contexts (Fig. 1b).

To enhance phage propagation in the selection circuit, we hypothesized that a DdCBE architecture with maximal editing efficiency would provide a favorable starting point to evolve activity against TC and non-TC targets. We designed a DdCBE that consisted of a left-side TALE (TALE3) and a right-side TALE (TALE4) flanking a 15-base pair (bp) spacing region, with targets C_6_ and C_7_ within the transcription template strand (Fig. 1c). We fused one copy of UGI to the N-terminus of the TALE protein and split DddA at G1397 to maximize editing of cytosine targets in the transcription template strand^18^. The resulting UGI–TALE3–DddA-G1397-N and UGI–TALE4–DddA-G1397-C fusions, which we refer to hereafter as T7-DdCBE, were encoded in the selection phage (SP) to co-evolve both halves of DdCBE (Fig. 1a). The phage genome is continuously mutagenized by an arabinose-inducible mutagenesis plasmid (MP6)^28^ (Fig. 1a).

**Extended Data Fig. 1:**
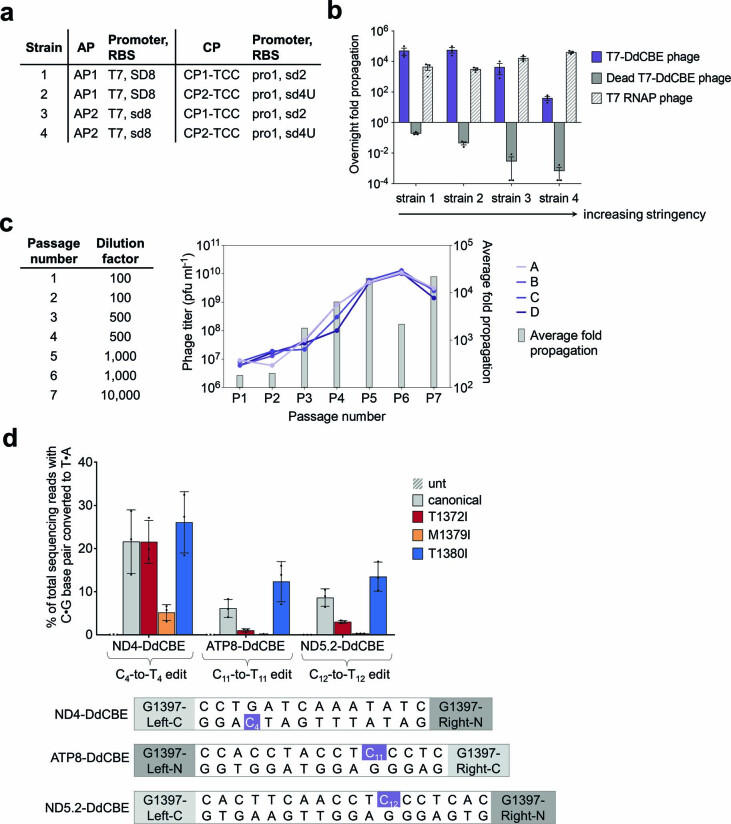
Evolution of canonical T7-DdCBE for improved TC activity using PANCE. **a**, Strains for screening selection stringency. Strains were generated by transformation with a variant of an AP and a variant of a CP. All CPs encode a TCC linker. Relative RBS strengths of SD8, sd8, sd2 and sd4U are 1.0, 0.20, 0.010 and 0.00040, respectively. **b**, Overnight phage propagation of indicated SPs in host strains with increasing stringencies. Dead T7-DdCBE phage contained the catalytically inactivating E1347A mutation in DddA. The fold phage propagation is the output phage titer divided by the input titer. **c**, Phage passage schedule for canonical T7-DdCBE evolution in PANCE using strain 4 transformed with MP6. Table indicates the dilution factor for the input phage population. Output phage titers for each replicate (A, B, C and D) are shown for each passage. Average fold propagation was obtained by averaging the fold propagation obtained from the four replicates A-D. **d**, Mitochondrial base editing efficiencies of HEK293T cells treated with canonical DdCBE or with DdCBEs containing the indicated mutations within DddA. For each base editor, the DddA split orientation and target cytosine (purple) within the spacing region is indicated. For b and d, values and error bars reflect the mean±s.d of n = 3 independent biological replicates.

To modulate selection stringency, we generated host strains 1–4. Each host strain contained combinations of AP and CP with different ribosome-binding-site strengths, such that strain 1 resulted in the lowest selection stringency and strain 4 provided the highest stringency. All tested CPs encoded the TCC linker sequence (Extended Data Fig. 1a). We then tested overnight SP propagation in these host strains. At the highest stringency, we observed ~100-fold overnight phage propagation of an SP containing an active T7-DdCBE, consistent with DdCBE’s ability to edit 5′-TC targets. Notably, phage containing an inactivating E1347A DddA mutation (dead T7-DdCBE phage) did not propagate (Extended Data Fig. 1b). These results establish the dependence of phage propagation on DdCBE activity and that BE-PACE can be successfully adapted to select TALE-based DdCBEs.

### Phage-assisted evolution of DdCBE toward higher editing efficiency at 5′-TC

We reasoned that beginning evolution with PANCE may be useful to increase activity and phage propagation before moving into PACE^26^. PANCE is less stringent because fresh host cells are manually infected with SP from a preceding passage, so no phage is lost to continuous dilution.

To evolve DdCBEs for higher activity at TC targets, we initiated PANCE of canonical T7-DdCBE by infecting SP into high-stringency strain 4 transformed with MP6 (Extended Data Fig. 1a). After seven passages, phage populations from all four replicates propagated approximately 10,000-fold overnight (Extended Data Fig. 1c). Isolated clonal phages from two or more independent replicates were enriched for the mutations T1372I, M1379I and T1380I within the DddA gene (Supplementary Table 1).

**Fig. 2:**
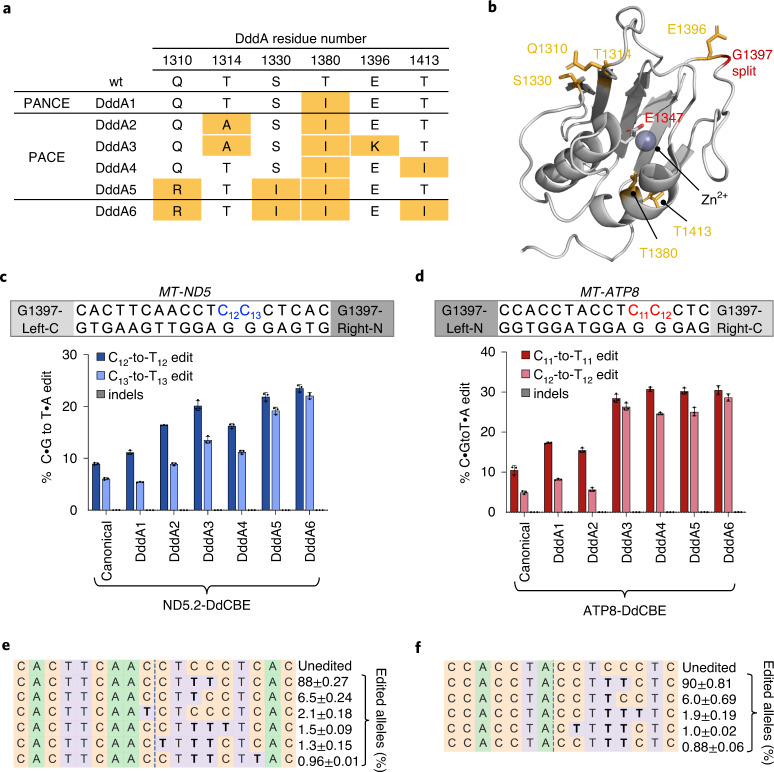
Evolved DddA variants improve mitochondrial base editing activity at 5′-TC. **a**, Mutations within the DddA gene of T7-DdCBE. Variants were isolated after evolution of canonical T7-DdCBE using PANCE and PACE in strain 4 transformed with MP6 (Extended Data Fig. 1a). DddA6 was rationally designed by incorporating the T1413I mutation into DddA5. **b**, Crystal structure of DddA (gray, PDB 6U08) complexed with DddI immunity protein (not shown). Positions of mutations enriched after PANCE and PACE are colored in orange. The catalytic residue E1347 is shown. DddA was split at G1397 (red) to generate T7-DdCBE. **c**, **d**, mtDNA editing efficiencies and indel frequencies of HEK293T cells treated with ND5.2-DdCBE (**c**) or ATP8-DdCBE (**d**). The genotypes of DddA variants correspond to **a**. For each base editor, the DNA spacing region, target cytosines and DddA split orientation are shown. **e**, Frequencies of *MT-ND5* alleles produced by DddA6 in **c**. **f**, Frequencies of *MT-ATP8* alleles produced by DddA6 in **d**. For **e** and **f**, tables are representative of *n* = 3 independent biological replicates. For **c**–**f**, values and errors reflect the mean ± s.d. of *n* = 3 independent biological replicates.

To validate the editing activity associated with these DddA genotypes, we incorporated each mutation into our previously published G1397 split DdCBEs that targeted human *MT-ATP8*, *MT-ND4* and *MT-ND5* (Supplementary Note 1)^18^. We plasmid-transfected HEK293T cells with canonical versions of ATP8-DdCBE, ND4-DdCBE or ND5.2-DdCBE and compared their editing efficiencies to those produced from the corresponding mutant DdCBEs. Although T1372I and M1379I impaired editing, T1380I increased C•G-to-T•A conversions by an average of 1.2-fold to 2.0-fold across the three mtDNA sites (Extended Data Fig. 1d). It is possible that the benefit of T1372I and M1379I may require additional mutations evolved during PANCE but not tested in mammalian cells. These results indicate that PANCE of canonical T7-DdCBE was able to yield a DddA variant that modestly improved TC editing. We refer to the DddA (T1380I) mutant as DddA1 (Fig. 2a).

To further increase selection stringency, we conducted PACE using an SP encoding the DddA1 variant of T7-DdCBE (T7-DdCBE-DddA1). After 140 hours of continuous propagation at a flow rate of 1.5–3.0 lagoon volumes per hour, distinct mutations enriched across the four replicates, with the starting T1380I mutation maintained in all lagoons (Supplementary Table 2). We selected the most enriched genotype in each of the four replicates (DddA2, DddA3, DddA4 and DddA5) and tested their mtDNA editing efficiencies (Fig. 2a,b). DddA2, DddA3, DddA4 and DddA5 improved average TC editing efficiencies from 7.6 ± 2.4% with starting DddA to 14 ± 5.8%, 22 ± 6.1%, 21 ± 7.9% and 24 ± 4.4%, respectively, within *MT-ND5* and *MT-ATP8* (Fig. 2c,d).

The T1413I mutation in DddA4, which is in the C-terminal half of split DddA, improved base editing efficiency of DddA4 by an average of 1.6-fold compared to DddA1. Given that T1413I is positioned along the interface between the two split DddA halves (Fig. 2b), we hypothesized that this mutation might promote the reconstitution of split DddA halves. Incorporating T1413I into DddA5 to form DddA6 (Q1310R + S1330I + T1380I + T1413I) resulted in a modest editing efficiency improvement to 26 ± 3.7%, a 3.4-fold average improvement in TC editing activity compared to wild-type DddA (Fig. 2c d). Close to 90% of the edited alleles produced from DddA6 contained a TCC-to-TTT conversion, suggesting that consecutive cytosines are likely targets for processive base editing (Fig. 2e,f). These results establish DddA6 as a dsDNA cytidine deaminase variant with enhanced editing activity at TC sequences.

**Extended Data Fig. 2:**
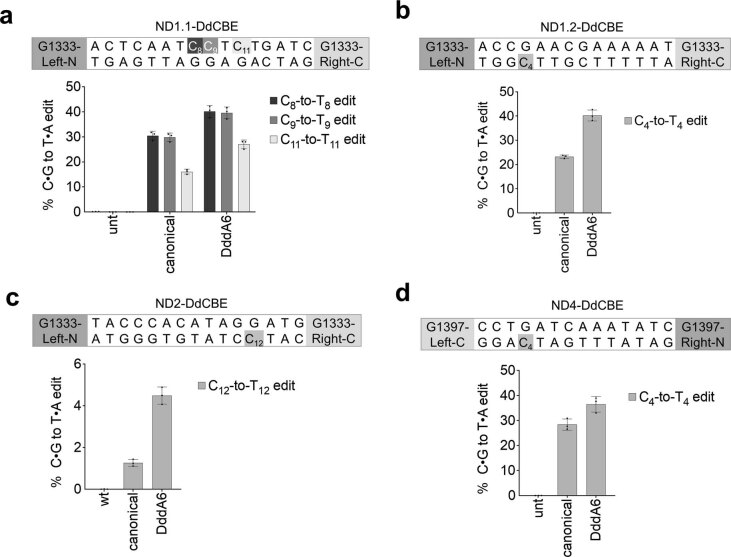
DddA6 is compatible with split-G1333 and split-G1397 DdCBE orientations. **a-d**, Mitochondrial base editing efficiencies of HEK293T cells treated with (**a**) ND1.1-DdCBE, (**b**) ND1.2-DdCBE, (**c**) ND2-DdCBE and (**d**) ND4-DdCBE. Target spacing regions and split DddA orientations are shown above each plot. Values and error bars reflect the mean±s.d of n = 3 independent biological replicates.

We evolved DddA6 from DddA split at G1397. To check if DddA6 is compatible with the G1333 split, we tested DddA6 at three mtDNA sites using DdCBEs split at G1333 and observed a 1.3-fold to 3.6-fold improvement in editing efficiencies compared to wild-type DddA (Extended Data Fig. 2a–c). These data indicate that mutations in DddA6 can enhance mtDNA editing efficiencies of the G1333 split variant, but the extent of improvement is lower than with the G1397 split. We noted that editing improvements mediated by DddA6 were modest at sites that exhibit efficient editing even with wild-type DddA, such as *MT-ND1* and *MT-ND4* (Extended Data Fig. 2a,d). For sites already efficiently edited with canonical DdCBEs, other deaminase-independent factors, such as mtDNA repair, could limit editing efficiency more than deaminase activity.

### Evolving DddA variants with expanded sequence context compatibility

**Fig. 3:**
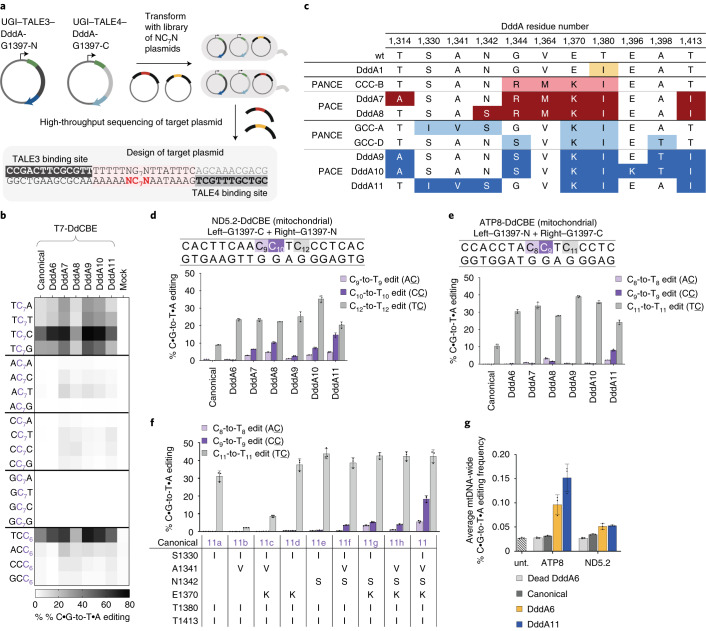
Evolved DddA variants show enhanced editing at TC and non-TC target sequences in mtDNA. **a**, Bacterial plasmid assay to profile sequence preferences of evolved DddA variants. T7-DdCBE edits the NC_7_N sequence of the target plasmid library. **b**, Heat map showing C•G-to-T•A editing efficiencies of NC_7_N sequence in each target plasmid, including the second cytosine in NCC_6_ sequences. Genotypes of listed variants correspond to Figs. 2a and 3c. Mock-treated cells did not express T7-DdCBE and contained only the library of target plasmids. Shading levels reflect the mean of *n* = 3 independent biological replicates. **c**, Genotypes of DddA variants after evolving T7-DdCBE-DddA1 using context-specific PANCE and PACE. Mutations enriched for activity on a CCC linker or GCC linker are highlighted in red and blue, respectively. **d**, **e**, Mitochondrial C•G-to-T•A editing efficiencies of HEK293T cells treated with canonical and evolved variants of ND5.2-DdCBE (**d**) or ATP8-DdCBE (**e**). Target spacing regions and split DddA orientations are shown for each base editor. Cytosines highlighted in light purple and dark purple are in non-TC contexts. **f**, Mitochondrial base editing efficiencies of reversion mutants from ATP8-DdCBE-DddA11 (labeled as 11) in HEK293T cells. Reversion mutants are designated 11a–11h. Amino acids that differ from those in canonical ATP8-DdCBE are indicated, so the absence of an amino acid indicates a reversion to the corresponding canonical amino acid in the first column. **g**, Average percentage of genome-wide C•G-to-T•A off-target editing in mtDNA for indicated DdCBE and controls in HEK293T cells. For **d**–**g**, values and error bars reflect the mean ± s.d. of *n* = 3 independent biological replicates.

To assess if the enhanced activity of DddA6 would enable base editing at target cytosines not in the native TC sequence context, bacteria expressing the evolved T7-DdCBE were transformed with a plasmid library encoding NC_7_N targets, where N = A, T, C or G. After overnight incubation, the plasmid library was isolated and subjected to high-throughput sequencing to measure the C•G-to-T•A conversion at each of the 16 NC_7_N targets (Fig. 3a).

Consistent with earlier human mtDNA editing results, DddA6 improved the average editing efficiencies of bacterial plasmids containing TC_7_N substrates by approximately 1.3-fold. DddA6-mediated editing at non-TC sequences, however, remained negligible (<0.2%) (Fig. 3b), suggesting the need to further evolve DddA to deaminate non-TC targets.

**Extended Data Fig. 3:**
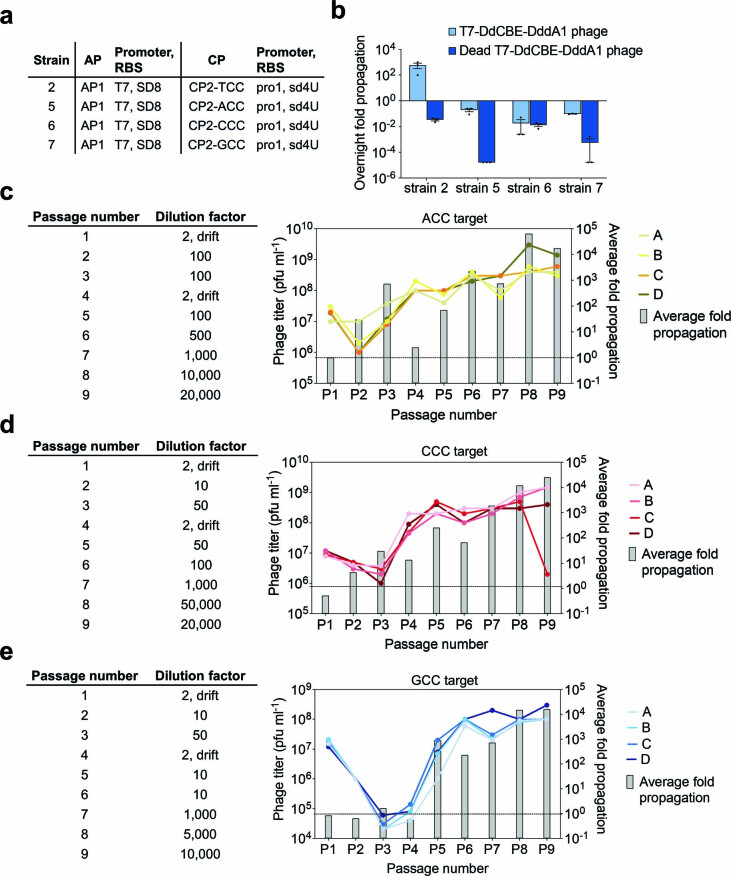
Evolution of DddA1-containing T7-DdCBE for expanded targeting scope using PANCE. **a**, Strains for overnight phage propagation assays on non-TC linker substrates. **b**, Overnight fold propagation of indicated SP in host strains encoding TC or non-TC linkers. Strains correspond to Extended Data Fig. 3a. T7-DdCBE-DddA1 phage harbors a T1380I mutation in DddA. Dead T7-DdCBE-DddA1 phage contains an additional catalytically inactivating E1347A mutation in DddA. Values and error bars reflect the mean±s.d of n = 3 independent biological replicates. **c-e**, Phage passage schedule for T7-DdCBE-DddA1 evolution in PANCE using (**c**) strain 5 transformed with MP6, (**d**) strain 6 transformed with MP6 or (**e**) strain 7 transformed with MP6. Tables indicate the dilution factor for the input phage population (see Methods for drifting procedure) For a given linker target, the output phage titers for each replicate (A, B, C and D) are shown for each passage. Average fold propagations above the dotted line in each graph represent propagation >1-fold. Average fold propagation was obtained by averaging the fold propagation obtained from the four replicates.

We modified the linker sequence in the CP to contain ACC, CCC or GCC (Fig. 1b). We co-transformed cells with AP1 and one of three CP plasmids (CP2-ACC, CP2-CCC or CP2-GCC) to generate three high-stringency *E. coli* strains (5, 6 and 7) for infection with SP encoding T7-DdCBE-DddA1 (Fig. 2 and Extended Data Fig. 3a). A large drop in overnight phage titers across strains 5, 6 and 7 suggested that the starting T7-DdCBE-DddA1 activity against non-TC sequences was too low to support PACE, so we initiated evolution with PANCE (Extended Data Fig. 3b). We first conducted a round of mutagenic drift to diversify the phage genome in the absence of selection pressure^29^. Next, we initiated three parallel PANCE campaigns of T7-DdCBE-DddA1 (PANCE-ACC, PANCE-CCC and PANCE-GCC). Each campaign was challenged with a non-TC linker and was conducted in four replicates (Extended Data Fig. 3c–e).

We isolated phage that propagated more than 10,000-fold overnight after nine passages of PANCE. The surviving DddA genotypes were strongly enriched for N1342S and E1370K mutations across all linker targets. Positions A1341 and G1344 were replaced with different amino acids depending on the linker target (Supplementary Table 3).

Given the substantial increase in phage propagation strength after nine PANCE passages (surviving, in total, ~10^16–^^19^-fold dilution), we increased selection stringency by challenging three surviving phage populations (PANCE-CCC-B, PANCE-GCC-A and PANCE-GCC-D) to 138 hours of PACE at a flow rate of 1.5–3.5 lagoon volumes per hour. For PACE, we used the same MP6-transformed strains 6 and 7 that had been applied earlier in PANCE (Extended Data Fig. 3a). The resulting PACE-evolved DddA variants acquired the additional mutations T1314A, E1396K and T1413I (Supplementary Table 4).

### Characterizing sequence context preferences of DddA variants

From each phage population that survived selection on a CCC or GCC linker target, we sequenced 6–8 clones. We chose five PACE-evolved DddA variants (DddA7, DddA8, DddA9, DddA10 and DddA11) for further characterization based on their genotypes (Fig. 3c). We profiled their sequence context preferences using the same bacterial NC_7_N plasmid assay used to characterize DddA6 (Fig. 3a).

All variants, except DddA8, maintained or improved editing efficiencies at TC (Fig. 3b). DddA9 and DddA10 resulted in approximately 2.0-fold higher TC editing than canonical T7-DdCBE but very low CC editing (<3.0%) (Fig. 3b). Although the average AC and CC editing levels by canonical T7-DdCBE were negligible (<0.66%), DddA7, DddA8 and DddA11 yielded an average of 4.3% editing at these contexts (Fig. 3b). These results demonstrate that PACE can be successfully applied to evolve for DddA variants that show expanded targeting activity beyond TC.

To validate the activity of these DddA variants in human mtDNA, we replaced wild-type DddA in ND5.2-DdCBE and ATP8-DdCBE with DddA7, DddA8, Ddd9, DddA10 or DddA11 (Supplementary Note 1). Consistent with bacterial plasmid editing results, DddA9 and DddA10 resulted in similar improvements in TC editing as DddA6 but did not exhibit consistent non-TC editing across multiple mtDNA sites (Fig. 3d,e).

**Extended Data Fig. 4:**
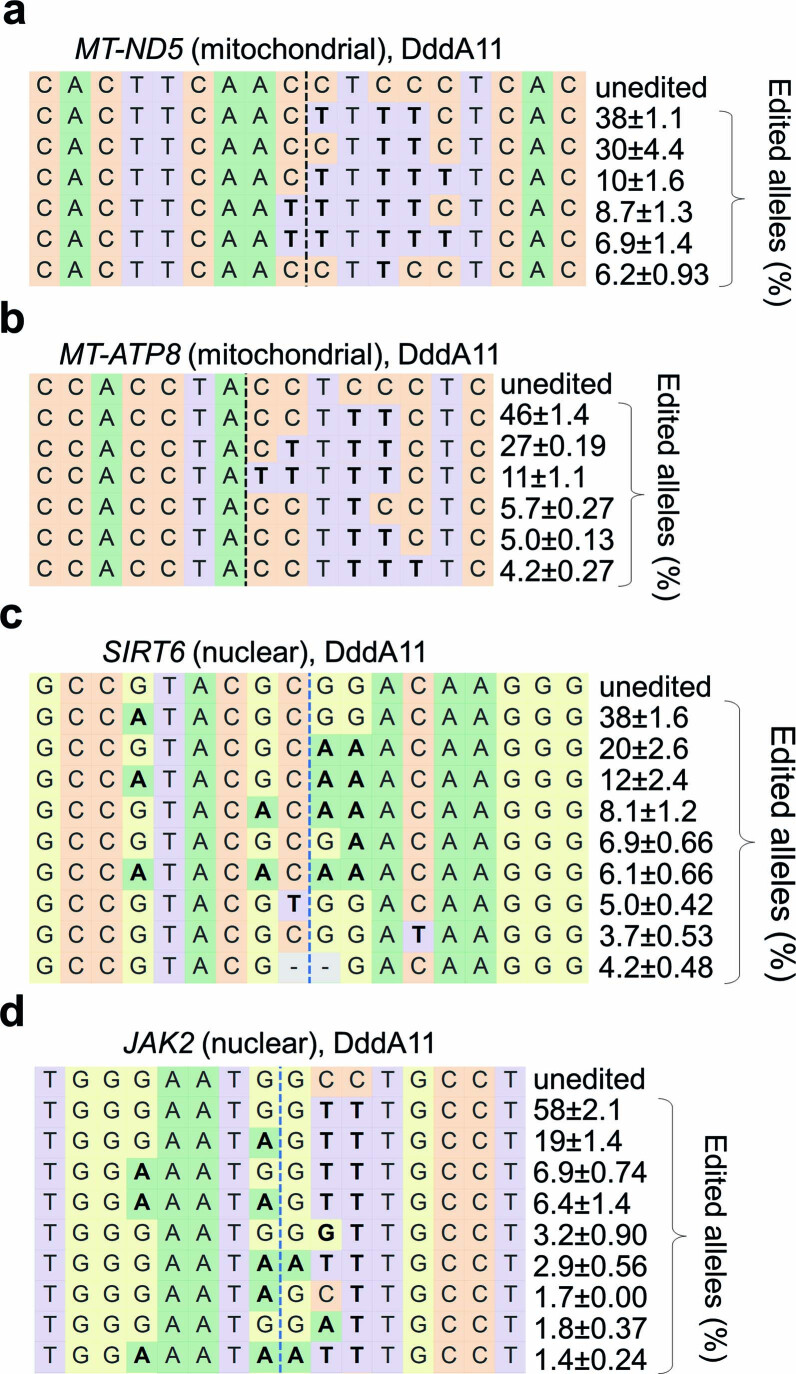
Allele compositions from mitochondrial and nuclear editing by DddA11-containing DdCBEs. **a**, Frequencies of mitochondrial *MT-ND5* alleles produced by DddA11 variant of ND5.2-DdCBE. **b**, Frequencies of mitochondrial *MT-ATP8* alleles produced by DddA11 variant of ATP8-DdCBE. **c**, Frequencies of nuclear *SIRT6* alleles produced by DddA11 variant of SIRT6-DdCBE. **d**, Frequencies of nuclear *JAK2* alleles produced by DddA11 variant of JAK2-DdCBE. Each table is representative of n = 3 independent biological replicates. Values and errors reflect the mean±s.d of n = 3 independent biological replicates.

Among variants tested, DddA11 supported the highest mtDNA editing efficiencies at AC (4.3–5.0%) and CC (7.6–16%) (Fig. 3d,e). Processive editing of consecutive cytosines in the spacing region could edit a preceding cytosine to a thymine, thus changing the starting ACC target into ATC_10_ in *MT-ND5* and ATC_9_ in *MT-ATP8* (Fig. 3d,e). To clarify if C_10_ and C_9_ are edited as ACC or ATC targets, we compared the percentage of edited alleles that contained an ACT or ATT product. We noted that most of the on-target edited alleles retained the preceding 5′-C (48% for ND5.2-DdCBE and 27% for ATP8-DdCBE) (Extended Data Fig. 4a,b). We thus classified C_10_-to-T_10_ and C_9_-to-T_9_ conversions mediated by ND5.2-DdCBE and ATP8-DdCBE, respectively, as editing of CC sequences (Fig. 3d,e).

Given that DddA11 resulted in the highest non-TC editing efficiencies, we generated eight reversion mutants of ATP8-DdCBE containing DddA11 (11a–h) to identify the contributions of individual mutations. Variants 11f, 11g and 11h resulted in detectable AC and CC conversions averaging 3.0%, indicating that a combination of at least two of the three mutations A1341V, N1342S and E1370K is sufficient to enable editing of AC and CC. To maximize non-TC editing, S13330I should be incorporated (Fig. 3f). These results collectively suggest that the combination of the six mutations—S1330I, A1341V, N1342S, E1370K, T1380I and T1413I—enable DddA11 to catalyze efficient base editing at AC and CC contexts that are poorly edited by canonical DdCBE.

**Extended Data Fig. 5:**
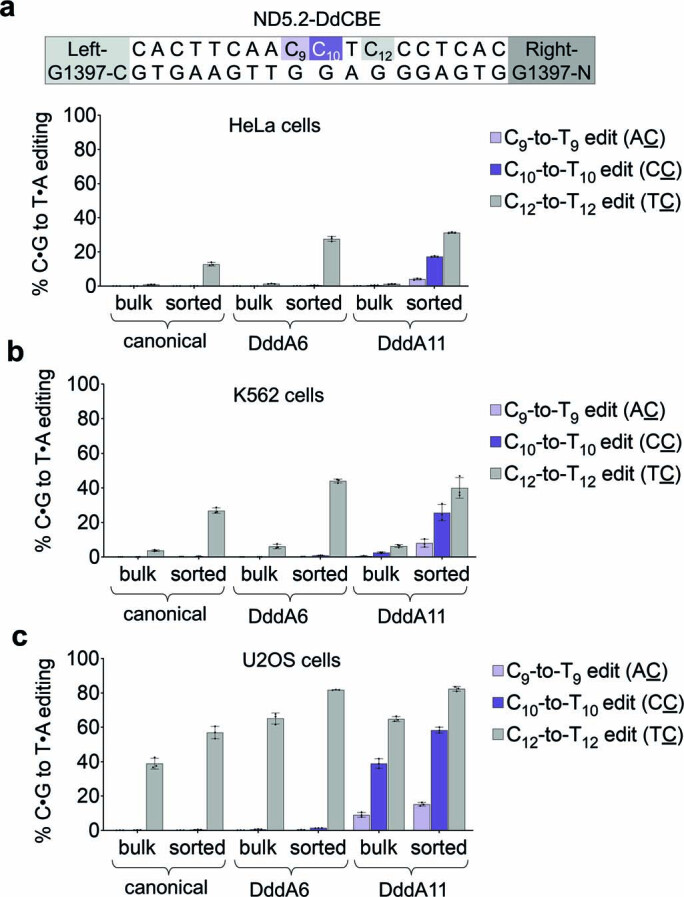
Evolved DddA variants mediate mitochondrial base editing in multiple human cell lines. **a-c**, Mitochondrial DNA editing efficiencies of canonical and evolved ND5.2-DdCBE in (**a**) HeLa cells, (**b**) K562 cells, and (**c**) U2OS cells. Editing efficiencies were measured for unsorted cells (bulk) and isolated DdCBE-expressing cells (sorted). Target spacing region and split DddA orientation are shown. Cytosines highlighted in light purple and dark purple are in non-TC contexts. Values and error bars reflect the mean±s.d of n = 3 independent biological replicates.

Next, we tested mitochondrial base editing by DddA6 and DddA11 in three other human cell lines. We fused fluorescent markers eGFP and mCherry to the right and left halves of ND5.2-DdCBE, respectively, by a self-cleaving P2A sequence^30^ to enable fluorescence-activated cell sorting (FACS) of nucleofected cells that express both halves of the DdCBE. For poorly transfected cells, such as HeLa, enriching for cells expressing eGFP and mCherry (eGFP^+^mCherry^+^) substantially increased TC and non-TC editing levels from less than 1% to 4–31% (Extended Data Fig. 5a and Supplementary Note 2). In K562 cells and U2OS cells, this enrichment strategy improved average editing by 11-fold and 1.5-fold, respectively, to efficiencies ranging from 20% to 60% (Extended Data Fig. 5b,c). These results indicate that cell lines other than HEK293T support improved mitochondrial base editing by evolved DddA variants.

### Mitochondrial off-target activity of evolved DddA variants

To profile mitochondrial off-target editing activities of DdCBEs containing DddA6 and DddA11, we performed assay for transposase-accessible chromatin using sequencing (ATAC-seq) of whole mitochondrial genomes from HEK293T cells transfected with plasmids encoding canonical or evolved variants of ND5.2-DdCBE or ATP8-DdCBE. A sequencing depth of approximately 3,000–8,000× was obtained per sample (Supplementary Table 5).

**Extended Data Fig. 6:**
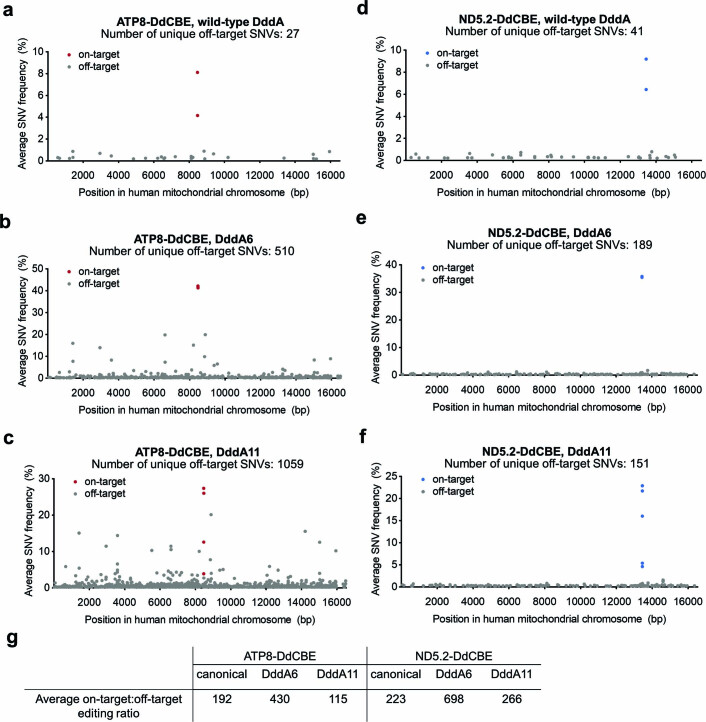
Mitochondrial genome-wide off-target C•G-to-T•A mutations. Average frequency and mitochondrial genome position of each unique C•G-to-T•A single nucleotide variant (SNV) is shown for HEK293T cells treated with (**a**) canonical ATP8-DdCBE, (**b**) ATP8-DdCBE containing DddA6, (**c**) ATP8-DdCBE containing DddA11, (**d**) canonical ND5.2-DdCBE, (**e**) ND5.2-DdCBE containing DddA6 and (**f**) ND5.2-DdCBE containing DddA11. **g**, Ratio of average on-target:off-target editing for the indicated canonical and evolved DdCBE. The ratio was calculated for each treatment condition as: (average frequency of all on-target C•G base pairs)÷(average frequency of non-target C•G base pairs present in the mitochondrial genome).

Consistent with previous results^18^, the average frequencies of mtDNA-wide off-target editing arising from canonical DdCBEs (0.033 ± 0.002%) were similar to those of the untreated control or DdCBEs containing dead DddA6 (0.028 ± 0.001%) (Fig. 3g). Off-target frequencies associated with ND5.2-DdCBE were 1.5-fold higher for DddA6 and DddA11 compared to wild-type DddA and 3.0-fold to 4.8-fold higher for ATP8-DdCBE (Fig. 3g). We analyzed the average frequencies of all unique off-target single-nucleotide variants (SNVs) containing a C•G-to-T•A conversion in cells treated with ATP8-DdCBE or ND5.2-DdCBE variants (Extended Data Fig. 6a–f). Although all 27 SNVs in cells treated with canonical ATP8-DdCBE were less than 1.0% (Extended Data Fig. 6a), 76 and 159 SNVs with more than 1% editing were detected for cells treated with DddA6 and DddA11, respectively (Extended Data Fig. 6b,c). As expected, these results suggest that deaminase-dependent off-target editing increases in the presence of DddA variants with higher activity and expanded targeting scope, although the frequency of off-target editing per on-target editing event remains similar to that of canonical DdCBE (Extended Data Fig. 6g).

In addition to deaminase-dependent off-target editing, the TALE repeats also contribute to overall off-target activity. For ND5.2-DdCBEs containing DddA6 or DddA11, we observed fewer than four SNVs with more than 1% frequency—far lower than those observed in ATP8-DdCBE containing the same DddA6 or DddA11 (compare Extended Data Fig. 6b,c to 6e,f). We hypothesize that TALE repeats that bind promiscuously to multiple DNA bases are more likely to result in higher off-target editing when fused to the evolved DddA variants^31,32^.

These results collectively indicate that, although mtDNA off-target editing increases for DdCBEs that use DddA6 and DddA11, consistent with their higher activity and expanded targeting scope, ratios of off-target:on-target editing remain similar to canonical DdCBE (Extended Data Fig. 6g).

### Profiling the editing window of evolved DddA variants

**Fig. 4:**
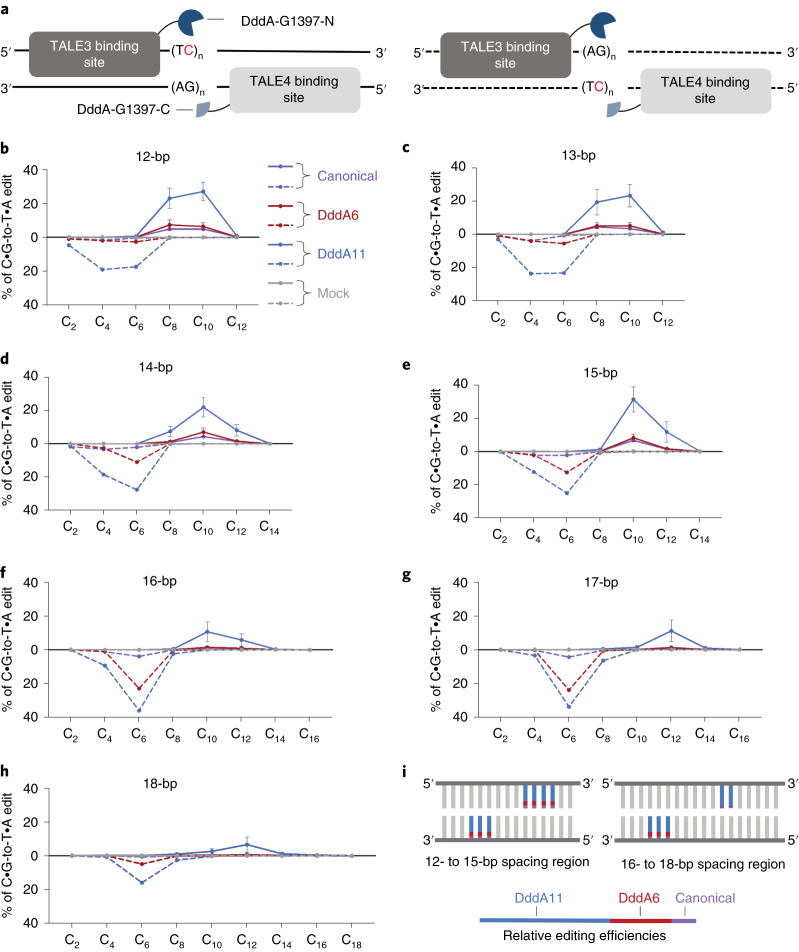
Editing windows of canonical and evolved T7-DdCBE. **a**, Split orientation of T7-DdCBE and its target spacing region. Each spacing region contains TC repeats within the top strand (left, solid line) or bottom strand (right, dashed line). Lengths of spacing regions ranged from 12 bp to 18 bp. **b**–**h**, Editing efficiencies mediated by canonical DdCBE (purple), DddA6-containing DdCBE (red) and DddA11-containing DdCBE (blue) are shown for each cytosine positioned within the spacing region length of 12 bp (**b**), 13 bp (**c**), 14 bp (**d**), 15 bp (**e**), 16 bp (**f**), 17 bp (**g**) and 18 bp (**h**). Subscripted numbers refer to the positions of cytosines in the spacing region, counting the DNA nucleotide immediately after the binding site of TALE3 as position 1. Editing efficiencies associated with the top and bottom strand are shown as solid and dashed lines, respectively. Mock-treated cells contained only the library of target plasmids (gray). For **b**–**h**, values and error bars reflect the mean ± s.d. of *n* = 3 independent biological replicates. **i**, Approximate editing windows for canonical (purple), DddA6 (red) and DddA11 (blue) variants of T7-DdCBE containing the G1397 split. The length of each colored line reflects the approximate relative editing efficiency for each DddA variant.

We wondered if the improved activity of the evolved DddA6 and DddA11 variants might influence the editing window for DdCBE editing. Using the bacterial plasmid assay for context profiling, we generated a separate library of 14 target plasmids for editing by canonical and evolved T7-DdCBEs containing the G1397 split. The spacing region in each target plasmid contained repeats of TC sequences ranging from 12 bp to 18 bp. These repeats were positioned on the top or bottom DNA strand (Fig. 4a).

Across 12-bp to 18-bp spacing regions, DddA11 resulted in the highest editing efficiencies and widest editing window compared to wild-type DddA and DddA6 (Fig. 4b–h). Within 12-bp to 15-bp spacing regions, the canonical and evolved DdCBEs preferentially edited cytosines positioned 4–6 nucleotides upstream of the 3′ end of the bottom strand and 3–6 nucleotides upstream of the 3′ end of the top strand (Fig. 4b–e,i).

At 16-bp to 18-bp spacing regions, canonical and DddA6-containing DdCBEs maintained editing of target cytosines positioned six nucleotides upstream of the 3′ end of the bottom strand but failed to efficiently edit cytosines in the top strand (Fig. 4f–h). In contrast, DddA11 retained activity for top-strand cytosines positioned 6–7 nucleotides upstream of the 3′ end, but efficiencies were substantially lower compared to shorter spacing lengths (compare Fig. 4b–e to 4f–h). These results indicate that the editing windows of evolved variants split at G1397 were generally similar to those of canonical DdCBE, with DddA11 exhibiting a larger editing window for spacing lengths more than 15 bp (Fig. 4i).

### Evolved DdCBE edits nuclear DNA

**Fig. 5:**
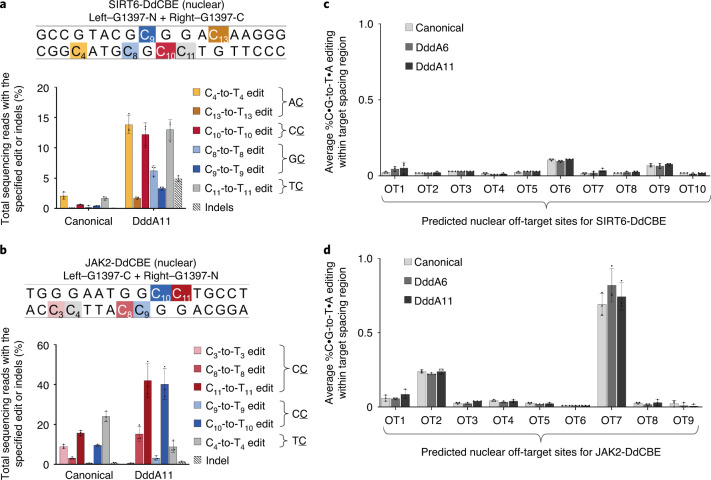
DddA11 expands targeting scope for nuclear DNA editing. **a**, **b**, Nuclear DNA editing efficiencies of HEK293T cells treated with the canonical or DddA11 variant of SIRT6-DdCBE (**a**) or JAK2-DdCBE (**b**). Target spacing regions and split DddA orientations are shown for each base editor. Cytosines highlighted in yellow, red or blue are in AC, CC or GC contexts, respectively. The architecture of each nuclear DdCBE half is bpNLS–2xUGI–4-amino-acid linker–TALE–[DddA half]. bpNLS, bipartite nuclear localization signal. **c**, **d**, Average frequencies of all possible C•G-to-T•A conversions within a predicted off-target spacing region associated with SIRT6-DdCBE (**a**) and JAK2-DdCBE (**b**). See Supplementary Table 6 for ranking of predicted off-target sites and Supplementary Table 8 for off-target site amplicons. For **a**–**d**, values and error bars reflect the mean ± s.d. of *n* = 3 independent biological replicates.

We previously showed that nuclear-localized DdCBE can mediate base editing at nuclear TC targets, which may provide useful alternatives to CRISPR CBEs when guide RNA or PAM requirements are limiting^18^ or when targeting heterochromatin sites^33^ (Supplementary Note 3). To test if DddA11 also expands the targeting scope of nuclear base editing, we transfected HEK293T cells with DdCBEs that targeted nuclear *SIRT6* or *JAK2* (ref. ^34^). When localized to the nucleus in the G1397 split orientation, DddA11 substantially improved AC, CC and GC editing from a typical range of 0–14% to 17–35% (Fig. 5a,b; see Extended Data Fig. 4c,d for frequencies of edited alleles). These results collectively show that DddA11 enhances non-TC editing efficiencies for all-protein base editing of both mitochondrial and nuclear DNA.

To assess for nuclear off-target editing, we used the off-target prediction tool PROGNOS^35^ to rank human nuclear DNA sequences that were predicted to be targeted by the TALE repeats in SIRT6- and JAK2-DdCBE. We treated HEK293T cells with the canonical or evolved DdCBEs and performed amplicon sequencing of the top 9–10 predicted off-target sites for each base editor (Supplementary Table 6). The average frequencies in which C•G base pairs within the predicted off-target spacing region were converted to T•A base pairs were very similar between the canonical and evolved DdCBEs (Fig. 5c,d). These results suggest that DddA6 and DddA11 did not increase nuclear off-target editing within a subset of computationally predicted off-target sites for a given pair of TALE repeats.

### Attempts to further increase activity at GC sequences

**Extended Data Fig. 7:**
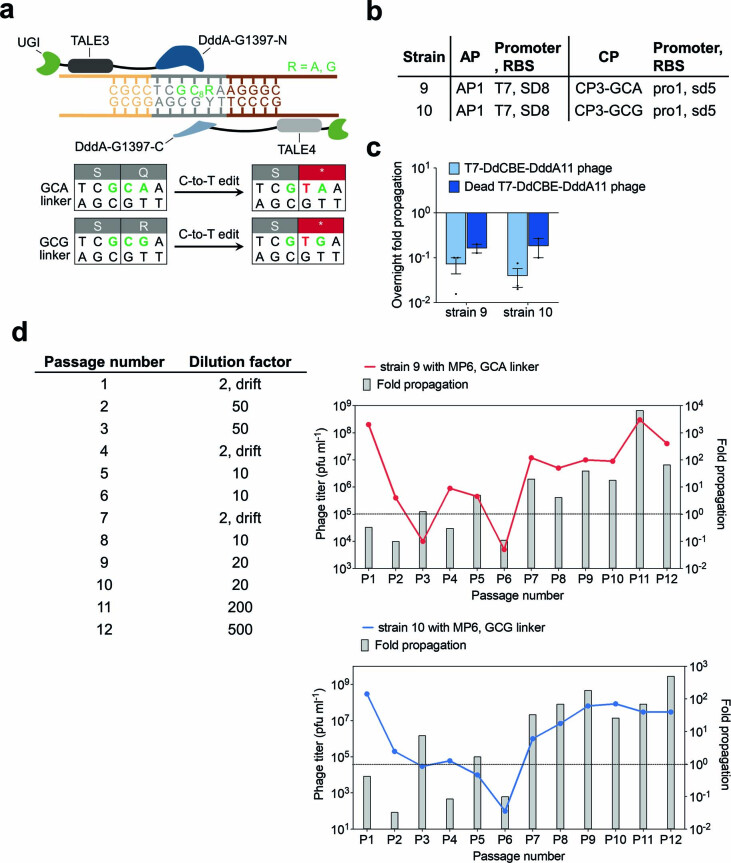
Evolution of T7-DdCBE-DddA11 using PANCE for improved GC activity. **a**, The sequence encoding the T7 RNAP–degron linker was modified to contain GCA or GCG in an effort to evolve for higher activity on GC targets. T7-DdCBE must convert GC_8_ to GT_8_ to install a stop codon in the linker sequence and restore T7 RNAP activity. **b**, Strains for overnight phage propagation assays on GCA or GCG linkers. **c**, Overnight fold propagation of indicated SP in host strains encoding GCA or GCG linkers. Strains correspond to Extended Data Fig. 7b. T7-DdCBE-DddA11 phage contains the mutations S1330I, A1341V, N1342S, E1370K, T1380I and T1413I in DddA. Dead T7-DdCBE-DddA11 phage contains an additional inactivating E1347A mutation in DddA. Values and error bars reflect the mean±s.d of n = 3 independent biological replicates**. d**, Phage passage schedule for T7-DdCBE-DddA11 evolution in PANCE using strain 9 transformed with MP6 (red) or strain 10 transformed with MP6 (blue). The table indicates the dilution factor for the input phage population (see Methods for drifting procedure). Output phage titer and fold propagation are shown for a single replicate. Fold propagations above the dotted line in each graph represent propagation >1-fold.

We noted that DddA11 was active mostly at GC_7_C_6_ and not GC_7_C_6_ (Fig. 3b). Given that a single C_6_-to-T_6_ conversion in a CC context is sufficient to generate a stop codon (Fig. 1b), the selection pressure to evolve acceptance of GC substrates was likely attenuated. To increase selection stringency, we modified the linker to encode either GC_8_A or GC_8_G such that only DddA variants that show activity at GC were able to restore active T7 RNAP (Extended Data Fig. 7a). We generated host strains 9 and 10 to contain the GCA and GCG linker, respectively (Extended Data Fig. 7b). Consistent with the weak GC activity of DddA11, we observed a drop in overnight phage titers in strains 9 and 10, suggesting that the activity of T7-DdCBE-DddA11 against GCA and GCG was too low to support PACE (Extended Data Fig. 7c and Supplementary Discussion).

**Extended Data Fig. 8:**
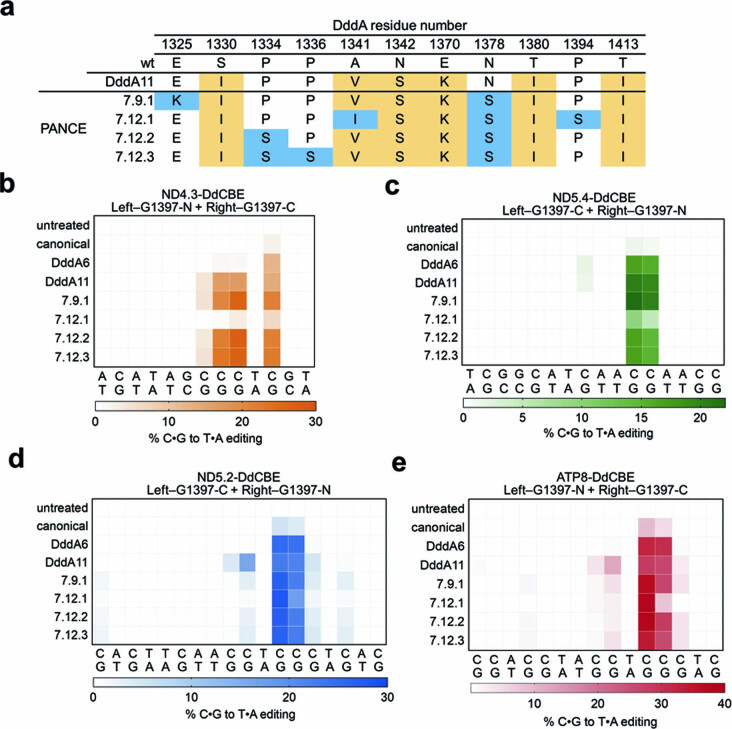
Mitochondrial editing efficiencies of DdCBE variants evolved from GC-specific PANCE. **a**, Enriched mutations within the DddA gene of T7-DdCBE after PANCE against a GCA or GCG linker. T7-DdCBE-DddA11 was used as the input SP for PANCE. DddA mutations in the input SP are shown in beige. Mutations enriched after 9 or 12 PANCE passages are shown in blue. **b-e**, Heat maps of mitochondrial base editing efficiencies of HEK293T cells treated with canonical and evolved variants of (**b**) ND4.3-DdCBE, (**c**) ND5.4-DdCBE (**d**) ND5.2-DdCBE and (**e**) ATP8-DdCBE. Target spacing regions and split DddA orientations are shown for each base editor. For b-e, colors reflect the mean of n = 3 independent biological replicates.

We initiated PANCE of T7-DdCBE-DddA11 in MP6-transformed host strains 9 and 10. After 12 passages, overnight phage propagation increased to approximately 100-fold to 1,000-fold (Extended Data Fig. 7d and Supplementary Discussion). We sequenced the surviving phage isolates from round 9 and round 12 to derive four consensus DddA genotypes (Extended Data Fig. 8a and Supplementary Table 7). The evolved variants did not improve editing efficiencies or targeting scope consistently across four mtDNA sites when compared with DddA11, although we noted that variant 7.9.1 showed higher editing efficiencies at TC targets compared to DddA6 and DddA11 (Extended Data Fig. 8b–e and Supplementary Discussion). These results suggest that DddA11 variants that can process GC substrates with improved efficiency are very rare.

### Installing previously inaccessible pathogenic mutations in mtDNA

**Fig. 6:**
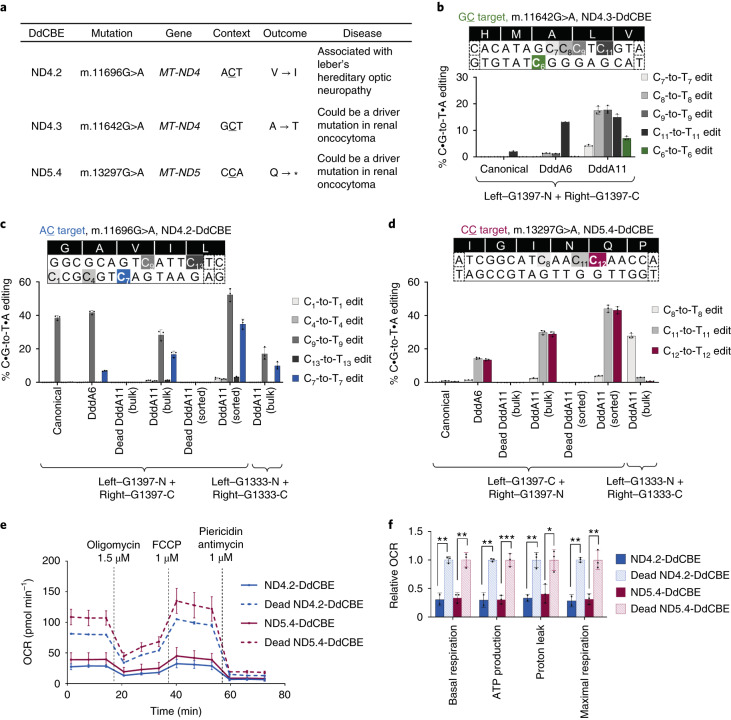
Application of DddA11 variant to install pathogenic mutations at non-TC targets in HEK293T cells. **a**, Use of DdCBEs to install disease-associated target mutations in human mtDNA. (V, valine; I, isoleucine; A, alanine; T, threonine; Q, glutamine; ∗, stop). **b**–**d**, Mitochondrial base editing efficiencies of HEK293T cells treated with canonical or evolved ND4.3-DdCBE (**b**), ND4.2-DdCBE (**c**) and ND5.4-DdCBE (**d**). On-target cytosines are colored green, blue or red, respectively. Cells expressing the DddA11 variant of DdCBE were isolated by FACS for high-throughput sequencing. The split orientation, target spacing region and corresponding encoded amino acids are shown. Nucleotide sequences boxed in dotted lines are part of the TALE binding site. **e**, **f**, Oxygen consumption rate (OCR) (**e**) and relative values of respiratory parameters (**f**) in sorted HEK293T cells treated with the DddA11 variant of ND4.2-DdCBE or ND5.4-DdCBE. For **b**–**f**, values and error bars reflect the mean ± s.d. of *n* = 3 independent biological replicates, except that ND4.2-DdCBE in **e** and **f** reflects *n* = 2 independent biological replicates. *P < 0.05, **P < 0.01 and ***P < 0.001 by Student’s unpaired two-tailed *t*-test.

To demonstrate the utility of evolved DddA variants with broadened sequence context compatibility, we designed three DdCBEs to install disease-associated C•G-to-T•A mutations at non-TC positions in human mtDNA. ND4.2-DdCBE installs the missense m.11696 G > A mutation in an ACT context. This mutation is associated with Leber’s hereditary optic neuropath^36^. ND4.3-DdCBE installs the missense m.11642 G > A mutation in a GCT context, and ND5.4-DdCBE installs the nonsense m.13297 G > A mutation in a CCA context. Both mutations were previously implicated in renal oncocytoma^37^ (Fig. 6a).

**Extended Data Fig. 9:**
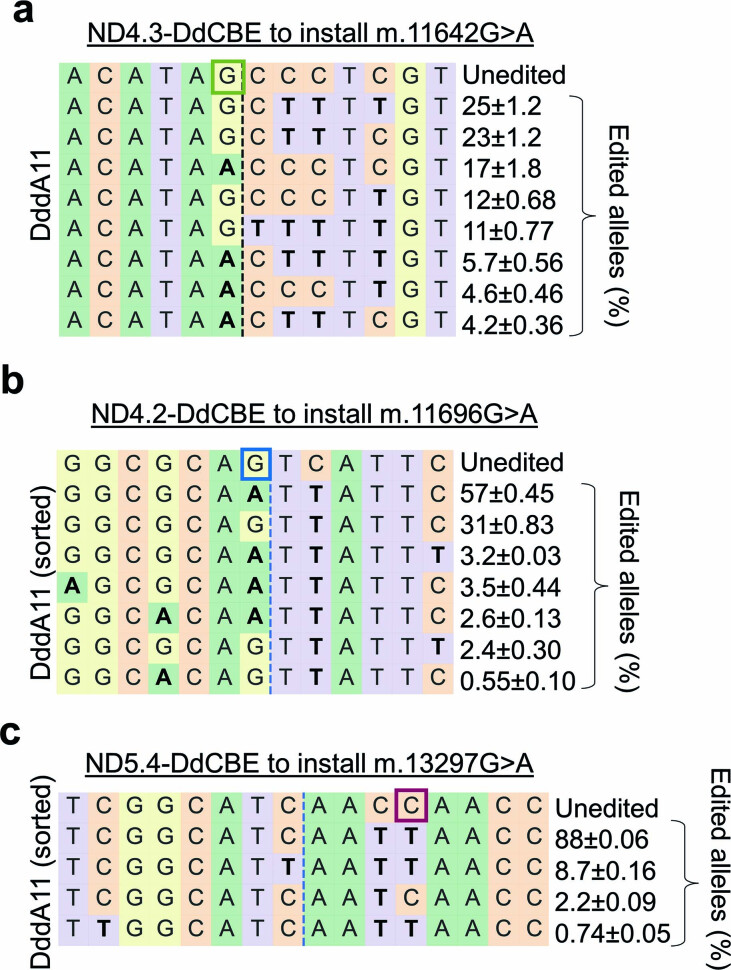
Allele compositions at disease-relevant mtDNA sites in HEK293T cells following base editing by DddA11-containing DdCBE variants. **a-c**, Allele frequency table of HEK293T cells treated with DddA11-containing (**a**) ND4.3-DdCBE, (**b**) ND4.2-DdCBE and (**c**) ND5.4-DdCBE to install the non-TC mutations m.11642 G > A, m.11696 > A and m.13297 G > A, respectively. On-target cytosines are boxed. Each table is representative of n = 3 independent biological replicates. Values and errors reflect the mean±s.d of n = 3 independent biological replicates.

We compared the editing efficiencies among DdCBEs containing wild-type DddA, DddA6 or DddA11 split at G1397. Although canonical DdCBEs resulted in negligible editing at non-TC sites, DddA11 edited the on-target cytosines at efficiencies ranging from 7.1% to 29% in bulk HEK293T cell populations (Fig. 6b–d). Despite the very low levels of bacterial GC editing mediated by DddA11 (Fig. 3b), DddA11 yielded 7.1 ± 0.69% on-target GC_6_ editing when tested in ND4.3-DdCBE (Fig. 6b). Most of the alleles containing the on-target edit, however, also harbored bystander edits that resulted in unintended changes to the protein-coding sequence (Extended Data Fig. 9a).

Unlike ND4.3-DdCBE, ND4.2-DdCBE and ND5.4-DdCBE resulted in higher bulk editing efficiencies ranging from 17% to 29% (Fig. 6c,d). More than 57% of the edited alleles contained the desired C•G-to-T•A on-target edit and a silent bystander edit (Extended Data Fig. 9b,c). DddA11 tested in the G1333 orientation resulted in lower on-target editing compared to the G1397 orientation (Fig. 6c). No on-target editing was detected with DddA11 when the target cytosine falls outside the preferred editing window of the G1333 split^18^ (Fig. 6d). These results collectively indicate that DddA11 tested in the G1397 split orientation enables much higher levels of base editing at AC, CC and GC targets than wild-type DddA, even though absolute editing levels at GC targets are modest.

Next, we assessed the phenotypic consequences of installing the missense m.11696 G > A mutation and the nonsense m.13297 G > A mutation in human mtDNA. We sorted for eGFP^+^mCherry^+^ cells to enrich those that expressed both halves of DdCBE. This enrichment increased on-target editing from 17% to 29% in unsorted cells to 35–43% in sorted cells (Fig. 6c,d). Compared to sorted cells containing dead DddA, sorted cells treated with DddA11-containing ND4.2-DdCBE and ND5.4-DdCBE exhibited reduced rates of basal and uncoupled respiration (Fig. 6e,f). These results establish that DddA11 can install candidate pathogenic mutations that canonical DdCBEs are unable to access and that these edits can occur at levels sufficient to result in altered mitochondrial function. These capabilities could broaden disease-modeling efforts using mitochondrial base editing.

## Discussion

Our recently reported DdCBEs enable installation of precise mutations within mtDNA for the first time, but target cytosines are primarily limited to 5′-TC contexts, and some target sites are edited with low efficiencies (≤5%)^18^. To address these challenges, we applied PACE to rapidly evolve for DddA variants, DddA6 and DddA11, that function in DdCBEs to mediate mitochondrial and nuclear base editing. These variants improve editing activity at TC and previously inaccessible non-TC targets while maintaining similarly high on-target:off-target editing ratios.

To edit TC targets, we recommend starting with canonical DdCBE. If editing efficiency is low, DddA6 can be used to increase editing while minimizing bystander editing of any cytosines in the editing window that are not preceded by a T.

For non-TC targets, DddA11 enables editing of AC and CC targets much more efficiently than canonical DdCBE. DddA11 typically supports higher C•G-to-T•A conversion in the G1397 split orientation compared to the G1333 split orientation. It is possible that initiating PACE with an SP encoding a G1333 split may result in mutations distinct from those found in DddA11. In addition, encoding the UGI on the C-terminus of T7-DdCBE during PACE may enrich additional mutations that favor the reassembly of DdCBEs containing this architecture.

Given that DddA11 is active at TC, AC and CC contexts, bystander editing is more likely with DddA11 than with canonical DdCBE. To minimize bystander editing, users may design different TALE binding sites that reduce the number of non-target cytosines within the editing window (Fig. 4i). Future efforts to develop context-specific cytidine deaminases will further minimize bystander base editing and off-target activity^38^.

**Extended Data Fig. 10:**
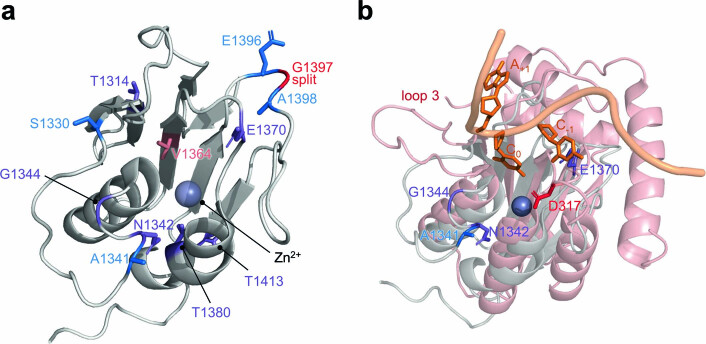
Structural alignment of DddA with ssDNA-bound APOBEC3G. **a**, Crystal structure of DddA (grey, PDB 6U08) complexed with DddI immunity protein (not shown). Positions of mutations common to the CCC- and GCC-specific evolutions are colored in purple. Additional mutations are colored according to Fig. 3c. DddA was split at G1397 (red) to generate T7-DdCBE for PANCE and PACE. **b**, DddA (PDB 6UO8, grey) was aligned to the catalytic domain of APOBEC3G (PDB 2KBO, red) complexed to its ssDNA 5’-CCA substrate (orange) using Pymol. The target C undergoing deamination by APOBEC3G is indicated as C_0_. Reversion analysis on the DddA11 mutant indicated that A1341V, N1342S and E1370K are critical for expanding the targeting scope of DddA (see Fig. 3f). D317 (red) confers 5’-CC specificity in APOBEC3G and loop 3 controls the catalytic activity of the APOBEC3G.

Additional protein evolution or engineering could further improve the editing efficiency of DddA variants, especially at GC targets. Although the structure of DddA bound to its dsDNA target is currently unavailable, structural alignment of DddA to existing deaminases with altered sequence specificities could offer insights for further DddA engineering efforts (Extended Data Fig. 10 and Supplementary Discussion).

## Methods

### General methods and molecular cloning

Antibiotics (Gold Biotechnology) were used at the following working concentrations: carbenicillin 100 μg ml^−1^, spectinomycin 50 μg ml^−1^, chloramphenicol 25 μgml^−1^, kanamycin 50 μg ml^−1^, tetracycline 10 μg ml^−1^ and streptomycin 50 μg ml^−1^. Nuclease-free water (Qiagen) was used for PCR reactions and cloning. For all other experiments, water was purified using a MilliQ purification system (Millipore). PCR was performed using Phusion U Green Multiplex PCR Master Mix (Thermo Fisher Scientific), Phusion U Green Hot Start DNA Polymerase (Thermo Fisher Scientific) or Phusion Hot Start II DNA polymerase (Thermo Fisher Scientific). All plasmids were constructed using USER cloning (New England Biolabs) and cloned into Mach1 chemically competent *E. coli* cells (Thermo Fisher Scientific). Unless otherwise noted, plasmid or SP DNA was amplified using the Illustra Templiphi 100 Amplification Kit (GE Healthcare Life Sciences) before Sanger sequencing. Plasmids for bacterial transformation were purified using the Qiagen Miniprep Kit according to the manufacturer’s instructions. Plasmids for mammalian transfection were purified using the Qiagen Midiprep Kit according to the manufacturer’s instructions but with 1.5 ml of RNAse A (1,000 µg ml^−1^) added to resuspension buffer. Codon-optimized sequences for human cell expression were obtained from GenScript. The amino acid sequences of all DdCBEs and DddA variants are provided in Supplementary Sequences 1–3. A full list of bacterial plasmids used in this work is given in Supplementary Table 9. All graphs were plotted used Prism 8 (GraphPad).

### Preparation and transformation of chemically competent cells

Strain S2060 (ref. ^39^) was used in all phage propagation, plaque assays and PACE experiments. To prepare competent cells, an overnight culture was diluted 100-fold into 50 ml of 2×YT media (United States Biological) supplemented with tetracycline and streptomycin and grown at 37 °C with shaking at 230 r.p.m. to OD_600_ ~0.4–0.6. Cells were pelleted by centrifugation at 4,000*g* for 10 minutes at 4 °C. The cell pellet was then resuspended by gentle stirring in 2.5 ml of ice-cold LB media (United States Biologicals), and then 2.5 ml of 2× TSS (LB media supplemented with 10% v/v DMSO, 20% w/v polyethylene glycol 3350 and 40 mM MgCl_2_) was added. The cell suspension was stirred to mix completely, aliquoted into 100-µl volumes and frozen on dry ice and stored at −80 °C until use.

To transform cells, 100 μl of competent cells thawed on ice was added to a pre-chilled mixture of plasmid (1–2 μl each; up to three plasmids per transformation) in 20 μl of 5× KCM solution (500 mM KCl, 150 mM CaCl_2_ and 250 mM MgCl_2_ in water) and 80 μl of water and stirred gently with a pipette tip. The mixture was incubated on ice for 20 minutes and heat-shocked at 42 °C for 75 seconds before 600 μl of SOC media (New England BioLabs) was added. Cells were allowed to recover at 37 °C with shaking at 230 r.p.m. for 1.5 hours, streaked on 2×YT media + 1.5% agar (United States Biological) plates containing the appropriate antibiotics and incubated at 37 °C for 16–18 hours.

### Bacteriophage cloning

For USER assembly of phage, 0.25 pmol of each PCR fragment was added to a make up a final volume of 25 µl. After USER assembly, the 25-µl USER reaction was transformed into 100 µl of chemicompetent S2060 *E. coli* host cells containing plasmid pJC175e^27^ that was modified to include constitutive DddI expression to minimize potential toxicity arising from split DddA expression in bacteria. This plasmid is referred to as pJC175e-DddI. Cells transformed with pJC175e-DddI enables activity-independent phage propagation and were grown overnight at 37 °C with shaking in antibiotic-free 2xYT media. Bacteria were then centrifuged for 2 minutes at 9,000*g* and were plaqued as described below. Individual phage plaques were grown in DRM media (prepared from United States Biological CS050H-001/CS050H-003) until the bacteria reached the late growth phase (~8 hours). Bacteria were centrifuged for 2 minutes at 9,000*g*, and the supernatants containing phage were filtered through a 0.22-μm PVDF Ultrafree centrifugal filter (Millipore) to remove residual bacteria and stored at 4 °C.

### Plaque assays for phage titer quantification and phage cloning

Phages were plaqued on S2060 (ref. ^39^) *E. coli* host cells containing plasmid pJC175e-DddI (for activity-independent propagation)^27^ or host cells transformed with AP and CP for activity-dependent propagation (see Supplementary Table 9 for the list of plasmids used in this study). To prepare a cell stock for plaquing, an overnight culture of host cells (fresh or stored at 4 °C for up to ~1 week) was diluted 50-fold in 2×YT medium containing appropriate antibiotics, and cells were grown at 37 °C to an OD_600_ of 0.8–1.0. Serial dilutions of phage (ten-fold) were made in 2×YT media. To prepare plates, molten 2×YT medium agar (1.5% agar, 55 °C) was mixed with Bluo-gal (4% w/v in DMF) to a final concentration of 0.08% Bluo-gal. The molten agar mixture was pipetted into quadrants of quartered Petri dishes (1.5 ml per quadrant) or wells of a 12-well plate (~1 milliliter per well) and was allowed to set. To prepare top agar, a 3:2 mixture of 2×YT medium and molten 2×YT medium agar (1.5%, resulting in a 0.6% agar final concentration) was prepared. To plaque, cell stock (100 µl or 150 µl for a 12-well plate or Petri dish, respectively), and phages (10 µl) were mixed in 2-ml library tubes (VWR International), and 55 °C top agar was added (400 µl or 1,000 µl for a 12-well plate or a Petri dish, respectively) and mixed one time by pipetting up and down, and then the mixture was immediately pipetted onto the solid agar medium in one well of a 12-well plate or one quadrant of a quartered Petri dish. Top agar was allowed to set undisturbed for 2 minutes at 25 °C, and then plates or dishes were incubated, without inverting, at 37 °C overnight. Phage titers were determined by quantifying blue plaques.

### Phage propagation assays

S2060 cells transformed with AP and CP plasmids of interest were prepared as described above and were inoculated in DRM. Host cells from an overnight culture in DRM were diluted 50-fold into fresh DRM and were grown at 37 °C to an OD_600_ of 0.3–0.4. Previously titered phage stocks were added to 1 ml of bacterial culture at a final concentration of ~10^5^ pfu ml^−1^. The cultures were grown overnight with shaking at 37 °C and were then centrifuged at 4,000*g* for 10 minutes to remove cells. The supernatants were titered by plaquing as described above. Fold enrichment was calculated by dividing the titer of phage propagated on host cells by the titer of phage at the same input concentration shaken overnight in DRM without host cells.

### Phage-assisted non-continuous evolution experiments

Host cells transformed with AP and CP were made chemically competent as described above. Chemically competent host cells were transformed with mutagenesis plasmid MP6 (ref. ^28^) and plated on 2× YT agar containing 10 mM glucose along with appropriate concentrations of antibiotics. Four colonies were picked into 1 ml of DRM each in a 96-well deep well plate, and this was diluted five-fold eight times serially into DRM. The plate was sealed with a porous sealing film and grown at 37 °C with shaking at 230 r.p.m. for 16–18 hour. Dilutions with OD_600_ ~0.3–0.4 were then treated with 10 mM arabinose to induce mutagenesis. Treated cultures were split into the desired number of 1-ml cultures in a 96-well plate and inoculated with selection phage at the indicated dilution (Extended Data Figs. 1c, 3c–e and 7d). Infected cultures were grown for 16–18 hours at 37 °C and harvested the next day via centrifugation at 4,000*g* for 10 minutes. Supernatant containing evolved phage was isolated and stored at 4 °C. Isolated phage were then used to infect the next passage, and the process was repeated for the duration of the selection. Phage titers were determined by plaque assay.

To initiate drift, phages from the previous passage were diluted two-fold by mixing with log-phase cells containing pJC175e-DddI and MP6. Phages were isolated after drifting for ~8 hours and mixed with the respective selection host strain for activity-dependent overnight phage propagation.

### Phage-assisted continuous evolution

Unless otherwise noted, PACE apparatus, including host cell strains, lagoons, chemostats and media, were all used as previously described^40^. Host cells were prepared as described for PANCE above. Four colonies were picked into 1 ml of DRM each in a 96-well deep-well plate, and this was diluted five-fold eight times serially into DRM. The plate was sealed with a porous sealing film and grown at 37 °C with shaking at 230 r.p.m. for 16–18 hours. Dilutions with OD_600_ ~0.4–0.8 were then used to inoculate a chemostat containing 80 ml of DRM. The chemostat was grown to OD_600_ ~0.6–0.8 and then continuously diluted with fresh DRM at a rate of 1–1.5 chemostat volumes per hour to keep the cell density roughly constant. The chemostat was maintained at a volume of 60–80 ml.

Before SP infection, lagoons were continuously diluted with culture from the chemostat at 1 lagoon volume per hour and pre-induced with 10 mM arabinose for at least 2 hours. Lagoons were infected with SP at a starting titer of 10^7^ pfu ml^−1^ and maintained at a volume of 15 ml. Samples (500 μl) of the SP population were taken at indicated times from lagoon waste lines. These were centrifuged at 9,000*g* for 2 minutes, and the supernatant was stored at 4 °C. Lagoon titers were determined by plaque assays using S2060 cells transformed with pJC175e-DddI. For Sanger sequencing of lagoons, single plaques were PCR amplified using primers AB1793 (5′-TAATGGAAACTTCCTCATGAAAAAGTCTTTAG) and AB1396 (5′- ACAGAGAGAATAACATAAAAACAGGGAAGC) to amplify UGI–TALE3-DddA-G1397-N; primers AR163 (5′-CCAGCAAGGCCGATAGTTTG) and AR611 (5′-CTAGCTGATAAATTCATGCCAG) amplified UGI–TALE3-DddA-G1397-C. Both sets of primers anneal to regions of the phage backbone flanking the evolving gene of interest. Generally, eight plaques were picked and sequenced per lagoon. Mutation analyses were performed using Mutato. Mutato is available as a Docker image at https://hub.docker.com/r/araguram/mutato.

See Supplementary Sequences 2 for sequences of all evolved DddA variants.

#### Evolution of canonical T7-DdCBE for improved TC activity

Host cells transformed with AP2, CP2-TCC and MP6 were maintained in an 80-ml chemostat. Four lagoons were each infected with SPBM13a (Supplementary Table 9). Upon infection, lagoon dilution rates were increased to 1.5 volumes per hour. Lagoon dilution rates were increased to 2 volumes per hour at 20 hours and 3 volumes per hour at 67 hours. The experiment ended at 139 hours.

#### Evolution of T7-DdCBE-CCC-B for broadened targeting scope

Host cells transformed with AP1, CP2-CCC and MP6 were maintained in a 50-ml chemostat. Two lagoons were each infected with phage pool CCC-B derived from PANCE. Upon infection, lagoon dilution rates were increased to 1.5 volumes per hour. Lagoon dilution rates were increased to 2.5 volumes per hour at 19 hours, 3 volumes per hour at 66 hours and 3.5 volumes per hour at 114 hours. The experiment ended at 138 hours.

#### Evolution of T7-DdCBE-GCC-A and T7-DdCBE-GCC-D for broadened targeting scope

Host cells transformed with AP1, CP2-GCC and MP6 were maintained in a 50-ml chemostat. One lagoon was infected with phage pool GCC-A, and a separate lagoon was infected with phage pool GCC-D. Both phage pools were derived from PANCE. Upon infection, lagoon dilution rates were increased to 1.5 volumes per hour. Lagoon dilution rates were increased to 2 volumes per hour at 66 hour, 2.5 volumes per hour at 90 hour and 3 volumes per hour at 114 hours. The experiment ended at 138 hours.

### Bacterial plasmid profiling assay for context preference and editing window profiling

To generate NCN target library, 16 µl of plasmids pBM10a to pBM10p were pooled (~100–200 ng µl^−1^, 1 µl each, Supplementary Table 9) and added to 100 μl of NEB 10-beta electrocompetent *E. coli*. To generate TC repeat library for profiling the editing window, 14 µl of plasmids pBM22a-g and pBM23a-g were pooled (~100–200 ng µl^−1^, 1 µl each, Supplementary Table 9) and added to 100 μl of NEB 10-beta electrocompetent *E. coli*. The resulting mixture was incubated on ice for 15 minutes before transferring into 4 × 25-µl aliquots in a pre-chilled 16-well Nucleocuvette strip. *E. coli* cells were electroporated with a Lonza 4D-Nucleofector System using bacterial program X-13. Freshly electroporated *E. coli* was immediately recovered in 1.4 ml of pre-warmed NEB Outgrowth media and incubated with shaking at 200 r.p.m. for 1 hour. After recovery, the 1.5 ml of culture was divided into two 750-µl aliquots for plating on two 245-mm square dishes (Corning) containing 2×YT medium agar (1.5% agar) mixed with 100 µg ml^−1^ of carbenicillin for plasmid maintenance. The dishes were incubated, without inverting, at 37 °C overnight. Colonies were scrapped from the plate the next day and resuspended in 50 ml of 2×YT media. The plasmid library was isolated with a Qiagen Midiprep Kit according to manufacturer’s instructions and was eluted in 100 μl of water.

To generate the T7-DdCBE-expressing host cells, ~20–50 µl of NEB 10-beta chemically competent *E. coli* was transformed with a plasmid from the pBM13 series to express the left-side TALE and a plasmid from the pBM14 series to express the right-side TALE (Supplementary Table 9), according to the manufacturer’s instructions. Cells were plated on 2×YT medium agar (1.5% agar) mixed with 50 µg ml^−1^ of spectinomycin, 50 µg ml^−1^ of kanamycin and 25 mM glucose. Glucose was added to minimize leaky expression of DdCBE. To make electrocompetent host cells, a single colony of DdCBE-expressing host cells was inoculated in 5–10 ml of DRM media and grown at 37 °C with shaking at 200 r.p.m. Cells were grown to OD_600_ ~0.4 and chilled on ice for ~10 minutes before centrifuging at 4,000*g* for 10 minutes. Supernatant was discarded, and the cell pellet was resuspended with 500–1000 µl of ice-cold 10% glycerol. The process was repeated for four glycerol washes. On the last wash, cells were resuspended in 50 µl of 10% glycerol, mixed with 2 µl of NCN target library (20 ng total) and incubated on ice for 5 minutes. Cells were transferred into a pre-chilled 16-well Nucleocuvette strip (50 µl per well) and electroporated with a Lonza 4D-Nucleofector System using bacterial program X-5. Freshly electroporated *E. coli* was immediately recovered in 750 µl of pre-warmed NEB Outgrowth media and recovered by shaking at 200 r.p.m. for 10 minutes. After recovery, 20–40 ml of DRM was added with 100 µg ml^−1^ of carbenicillin, 50 µg ml^−1^ of spectinomycin, 50 µg ml^−1^ of kanamycin and 10 mM arabinose (to induce DdCBE expression). Cells were incubated with shaking at 200 r.p.m. overnight. Library and base editor plasmids were isolated with a Qiagen Midiprep Kit according to the manufacturer’s instructions and was eluted in 100 μl of water.

### General mammalian cell culture condition

HEK293T (American Type Culture Collection (ATCC) CRL-3216), U2OS (ATTC HTB-96), K562 (CCL-243) and HeLa (CCL-2) cells were purchased from ATCC and cultured and passaged in DMEM plus GlutaMAX (Thermo Fisher Scientific), McCoy’s 5A medium (Gibco), RPMI medium 1640 plus GlutaMAX (Gibco) or DMEM plus GlutaMAX (Thermo Fisher Scientific), respectively, each supplemented with 10% (v/v) FBS (Gibco, qualified). Cells were incubated, maintained and cultured at 37 °C with 5% CO_2_. Cell lines were authenticated by their respective suppliers and tested negative for mycoplasma.

### HEK293T human cell lipofection

Cells were seeded on 48-well collagen-coated plates (Corning) at a density of 1.6–2 × 10^5^ cells per milliliter 18–24 hours before lipofection in a volume of 250 µl per well. Lipofection was performed at a cell density of approximately 70%. For DdCBE experiments, cells were transfected with 500 ng of each mitoTALE monomer to make up 1,000 ng of total plasmid DNA. Lipofectamine 2000 (1.2 μl; Thermo Fisher Scientific) was used per well. Cells were harvested 72 hours after lipofection for genomic DNA extraction. Unless stated otherwise, the architecture of each DdCBE half is MTS–TALE–[DddA half]–2-amino-acid linker–UGI (see Supplementary Table 10 for a list of TALE binding sites and Supplementary Sequences 1 for DdCBE sequences).

### FACS

Cells treated with the DddA11 variant of ND4.2-DdCBE or ND5.4-DdCBE were seeded on six-well plates (Corning) at a density of 1.8 × 10^5^ cells per milliliter 18–24 hours before lipofection in a volume of 2 ml per well. Cells were transfected with 1.25 µg of each mitoTALE monomer to make up 2.5 µg of total plasmid DNA. Lipofectamine 2000 (10 μl; Thermo Fisher Scientific) was used per well. Medium was removed 72 hours after lipofection, and cells were washed once with 1× Dulbecco’s PBS (Thermo Fisher Scientific). Cells were trypsinzed (400 µl; Gibco) and quenched with DMEM media (2 ml). Cells were centrifuged at 200*g* for 4 minutes, media was aspirated, and cells were resuspended in 1× PBS, filtered through a cell strainer (BD Biosciences) and sorted using a LE-MA900 cell sorter (Sony). See Supplementary Note 2 for representative FACS examples and Supplementary Sequences 3 for sequences of P2A linker, eGFP and mCherry.

### U2OS, K562 and HeLa human cell nucleofection

Nucleofection was used for transfection in all experiments using K562, HeLa and U2OS cells. Next, 125 ng of each DdCBE expression plasmid (total 250 ng plasmid) was nucleofected in a final volume of 20 μl in a 16-well Nucleocuvette strip (Lonza). K562 cells were nucleofected using the SF Cell Line 4D-Nucleofector X Kit (Lonza) with 5 × 10^5^ cells per sample (program FF-120), according to the manufacturer’s protocol. U2OS cells were nucleofected using the SE Cell Line 4D-Nucleofector X Kit (Lonza) with 4 × 10^5^ cells per sample (program DN-100), according to the manufacturer’s protocol. HeLa cells were nucleofected using the SE Cell Line 4D-Nucleofector X Kit (Lonza) with 2 × 10^5^ cells per sample (program CN-114), according to the manufacturer’s protocol. Cells were harvested 72 hours after nucleofection for genomic DNA extraction.

### Genomic DNA isolation from mammalian cell culture

Medium was removed, and cells were washed once with 1× Dulbecco’s PBS (Thermo Fisher Scientific). Genomic DNA extraction was performed by the addition of 40–50 µl of freshly prepared lysis buffer (10 mM Tris-HCl (pH 8.0), 0.05% SDS and proteinase K (20 μg ml^−1^; Thermo Fisher Scientific)) directly into the 48-well culture plate. The extraction solution was incubated at 37 °C for 60 minutes and then 80 °C for 20 minutes. Resulting genomic DNA was subjected to bead cleanup with AMPure DNAdvance beads according to the manufacturer’s instructions (Beckman Coulter, A48705).

### High-throughput DNA sequencing of genomic DNA samples

Genomic sites of interest were amplified from genomic DNA samples and sequenced on an Illumina MiSeq as previously described^18^. Amplification primers containing Illumina forward and reverse adapters (Supplementary Table 11) were used for a first round of PCR (PCR1) to amplify the genomic region of interest. In brief, 1 µl of purified genomic DNA was used as input into PCR1. For PCR1, DNA was amplified to the top of the linear range using Phusion Hot Start II High-Fidelity DNA Polymerase (Thermo Fisher Scientific) according to the manufacturer’s instructions but with the addition of 0.5× SYBR Green Nucleic Acid Gel Stain (Lonza) in each 25-µl reaction. For all amplicons, the PCR1 protocol used was an initial heating step of 2 minutes at 98 °C, followed by an optimized number of amplification cycles (10 seconds at 98 °C, 20 seconds at 62 °C and 30 seconds at 72 °C). Quantitative PCR was performed to determine the optimal cycle number for each amplicon. The number of cycles needed to reach the top of the linear range of amplification is ~17–19 cycles for mtDNA amplicons and ~27–28 cycles for nuclear DNA amplicons. Barcoding PCR2 reactions (25 µl) were performed with 1 µl of unpurified PCR1 product and amplified with Phusion Hot Start II High-Fidelity DNA Polymerase (Thermo Fisher Scientific) using the following protocol: 98 °C for 2 minutes and then ten cycles of (98 °C for 10 seconds, 61 °C for 20 seconds and 72 °C for 30 seconds), followed by a final 72 °C extension for 2 minutes. PCR products were evaluated analytically by electrophoresis in a 1.5% agarose gel. After PCR2, up to 300 samples with different barcode combinations were combined and purified by gel extraction using the QIAquick Gel Extraction Kit (Qiagen). DNA concentration was quantified using the Qubit ssDNA HS Assay Kit (Thermo Fisher Scientific) to make up a 4 nM library. The library concentration was further verified by qPCR (KAPA Library Quantification Kit Illumina, KAPA Biosystems) and sequenced using an Illumina MiSeq with 210-bp to 300-bp single-end reads. Sequencing results were computed with a minimum sequencing depth of approximately 10,000 reads per sample.

#### High-throughput sequencing of NCN and TC repeat library plasmids

Primers T7-DdCBE Fwd and T7-DdCBE Rev were used to amplify the region containing the NCN target spacing region (Supplementary Table 11). In brief, 100 ng of purified plasmids was used as input into PCR1 in a total reaction volume of 50 µl. For PCR1, quantitative PCR was used to amplify DNA to the top of the linear range as described above (~14 cycles). PCR1 products were purified with QIAquick PCR Purification Kit according to the manufacturer’s instructions and eluted in 20 µl. Barcoding PCR2 reactions (50 µl) were performed with 10 µl of purified PCR1 product and amplified for eight cycles. Subsequent steps after PCR2 are as described above.

### Analysis of HTS data for DNA sequencing and targeted amplicon sequencing

Sequencing reads were demultiplexed using MiSeq Reporter (Illumina). Batch analysis with CRISPResso2 (version 2.0.34)^41^ was used for targeted amplicon and DNA sequencing analysis (see Supplementary Table 8 for a list of amplicon sequences used for alignment). A 10-bp window was used to quantify indels centered around the middle of the dsDNA spacing. To set the cleavage offset, a hypothetical 15-bp or 16-bp spacing region has a cleavage offset of −8. Otherwise, the default parameters were used for analysis. The output file ‘Reference.NUCLEOTIDE_PERCENTAGE_SUMMARY.txt’ was imported into Microsoft Excel (version 2201) for quantification of editing frequencies. Reads containing indels within the 10-bp window are excluded for calculation of editing frequencies. The output file ‘CRISPRessoBatch_quantification_of_editing_frequency.txt’ was imported into Microsoft Excel (version 2201) for quantification of indel frequencies. Indel frequencies were computed by dividing the sum of insertions and deletions over the total number of aligned reads.

#### Analysis of demultiplexed reads obtained from high-throughput sequencing of NCN and TC repeat target plasmids

A unique molecular identifier (UMI) was included within each target plasmid. The UMI served to distinguish reads that contained the unedited target sequence in the starting library from edited reads produced as a result of base editing (Supplementary Table 12). SeqKit package version 0.16.1 (grep)^42^ was used to assign FASTQ files containing a given UMI to its starting NCN target plasmid. Batch analysis with CRISPResso2 was performed as described above for quantification of editing frequencies.

### Bulk ATAC-seq for whole mitochondrial genome sequencing

ATAC-seq was performed as previously described^18^. In brief, 5,000–10,000 cells were trypsinzed, washed with PBS, pelleted by centrifugation and lysed in 50 µl of lysis buffer (0.1% Igepal CA-360 (v/v %), 10 mM Tris-HCl, 10 mM NaCl and 3 mM MgCl_2_ in nuclease-free water). Lysates were incubated on ice for 3 minutes, pelleted at 500 r.c.f. for 10 minutes at 4 °C and tagmented with 2.5 µl of Tn5 transposase (Illumina, 15027865) in a total volume of 10 µl containing 1×TD buffer (Illumina, 15027866), 0.1% NP-40 (Sigma-Aldrich) and 0.3× PBS. Samples were incubated at 37 °C for 30 minutes on a thermomixer at 300 r.p.m. DNA was purified using the MinElute PCR Kit (Qiagen) and eluted in 10 µl of elution buffer. All 10 µl of the eluate was amplified using indexed primers (1.25 μM each; sequences available as previously reported^18^) and NEBNext High-Fidelity 2× PCR Master Mix (New England Biolabs) in a total volume of 50 μl using the following protocol: 72 °C for 5 minutes, 98 °C for 30 seconds and then five cycles of (98 °C for 10 seconds, 63 °C for 30 seconds and 72 °C for 60 seconds), followed by a final 72 °C extension for 1 minute. After the initial five cycles of pre-amplification, 5 µl of partially amplified library was used as input DNA in a total volume of 15 µl for quantitative PCR using SYBR Green to determine the number of additional cycles needed to reach one-third of the maximum fluorescence intensity. Typically, 3–8 cycles were conducted on the remaining 45 µl of partially amplified library. The final library was purified using a MinElute PCR Kit (Qiagen) and quantified using a Qubit dsDNA HS Assay Kit (Invitrogen) and a High Sensitivity DNA chip run on a Bioanalyzer 2100 system (Agilent). All libraries were sequenced using NextSeq High Output Cartridge Kits on an Illumina NextSeq 500 sequencer. Libraries were sequenced using paired-end 2 × 75 cycles and demultiplexed using the bcl2fastq2 (version 2.20) program. A sequencing depth of ~3,000–8,000× was obtained per sample (Supplementary Table 5).

### Genome sequencing and SNP identification in mitochondria

Analysis was performed as previously described^18^. Genome mapping was performed with BWA (version 0.7.17) using the NC_012920 genome as a reference. Duplicates were marked using Picard tools (version 2.20.7). Pileup data from alignments were generated with SAMtools (version 1.9), and variant calling was performed with VarScan2 (version 2.4.3). Variants that were present at a frequency greater than 0.1% and a *P* value less than 0.05 (Fisher’s exact test) were called as high-confidence SNPs independently in each biological replicate. Only reads with *Q* > 30 at a given position were taken into account when calling SNPs at that particular position. For Extended Data Fig. 6a–f, all SNPs that were called in untreated samples were excluded from the analyses of treated samples. Each SNP was called in treated samples if it appeared in at least one biological replicate, and the average frequency was calculated by taking the average of all replicate(s) in which the SNP was present.

### Calculation of average off-target C•G-to-T•A editing frequency

Analysis was performed as previously described^18,43^. To calculate the mitochondrial genome-wide average off-target editing frequency for each DdCBE in Fig. 3g, REDItools was used (version 1.2.1)^44^. All nucleobases except cytosines and guanines were removed, and the number of reads covering each C•G base pair with a PHRED quality score greater than 30 (*Q* > 30) was calculated. The on-target C•G base pairs (depending on the DdCBE used in each treatment) were excluded to consider only off-target effects. C•G-to-T•A SNVs present at high frequencies (>50%) in both treated and untreated samples (that, therefore, did not arise from DdCBE treatment) were also excluded. The average off-target editing frequency was then calculated independently for each biological replicate of each treatment condition as: (number of reads in which a given C•G base pair was called as a T•A base pair, summed over all non-target C•G base pairs) / (total number of reads that covered all non-target C•G base pair).

### Oxygen consumption rate analyses by Seahorse XF analyzer

A Seahorse plate was coated with 0.01% (w/v) poly-ʟ-lysine (Sigma-Aldrich). Next, 1.6 × 10^4^ cells were seeded on the coated Seahorse plate 16 hours before the analysis in the Seahorse XFe96 Analyzer (Agilent). Analysis was performed in the Seahorse XF DMEM Medium pH 7.4 (Agilent) supplemented with 10 mM glucose (Agilent), 2 mM L-glutamine (Gibco) and 1 mM sodium pyruvate (Gibco). Mito stress protocol was applied with the use of 1.5 µM oligomycin, 1 µM FCCP and 1 µM piericidin + 1 µM antimycin.

### Reporting Summary

Further information on research design is available in the Nature Research Reporting Summary linked to this article.

## Online content

Any methods, additional references, Nature Research reporting summaries, source data, extended data, supplementary information, acknowledgements, peer review information; details of author contributions and competing interests; and statements of data and code availability are available at 10.1038/s41587-022-01256-8.

## Supplementary information

Supplementary Information Supplementary Discussion, Supplementary Tables 1–12, Supplementary Sequences 1–3, Supplementary Notes 1–3 and Supplementary References

Reporting Summary

Supplementary Table 1 Sequencing coverage for ATAC-seq samples

Supplementary Table 2 List of predicted off-target nuclear DNA sites for SIRT6-DdCBE and JAK2-DdCBE

## Data Availability

High-throughput sequencing and whole mitochondria sequencing data have been deposited in the National Center of Biotechnology Information Sequence Read Archive under accession code PRJNA753136. SNP identification for whole mitochondrial genome sequencing used the NC_012920 genome as a reference. Plasmids are available from Addgene. Amino acid sequences of all base editors in this study are provided in the Supplementary Information as Supplementary Sequences 1–3. TALE sequences for SIRT6-DdCBE and JAK2-DdCBE are from Addgene plasmids TAL2406, TAL2407, TAL2454 and TAL2455.
