# Supplementary material for: CRISPR-free base editors with enhanced activity and expanded targeting scope in mitochondrial and nuclear DNA
Source: Nat Biotechnol. 2022 Apr 4;40(9):1378–87. doi: 10.1038/s41587-022-01256-8 (PMC9463067; doi:10.1038/s41587-022-01256-8)
Supplement: Supplementary file 1 — Supplementary Discussion, Supplementary Tables 1–12, Supplementary Sequences 1–3, Supplementary Notes 1–3 and Supplementary References [file 41587_2022_1256_MOESM1_ESM.pdf]

nature portfolio

https://doi.org/10.1038/s41587-022-01256-8

Supplementary information

CRISPR-free base editors with enhanced activity and expanded targeting scope in mitochondrial and nuclear DNA

In the format provided by the authors and unedited

Supplementary Information

Continuous evolution of mitochondria base editors with improved activity and expanded targeting scope

Beverly Y. Mok[1,2,3], Anna V. Kotrys[4,5], Aditya Raguram[1,2,3], Tony P. Huang[1,2,3], Vamsi K. Mootha[4,5] and David R. Liu[1,2,3*]

	Page #
**Supplementary Discussion**	3-4
**Supplementary Table 1**. Mutation table of variants from PANCE of canonical T7-DdCBE for improved TC activity using PANCE	5
**Supplementary Table 2**. Mutation table of variants from PACE of T7-DdCBE-DddA1 for improved TC activity	6
**Supplementary Table 3**. Mutation table of variants from PANCE of T7-DdCBE-DddA1 for expanded targeting scope	7
**Supplementary Table 4**. Mutation table of variants from the PACE evolution to expand targeting scope	8
**Supplementary Table 5**. Sequencing coverage for ATAC-seq samples	Excel
**Supplementary Table 6**. List of predicted off-target nuclear DNA sites for SIRT6-DdCBE and JAK2-DdCBE Mutation table of variants from the GC-specific PANCE	Excel
**Supplementary Table 7**. Mutation table of variants from the GC-specific PANCE	11
**Supplementary Table 8**. Amplicons for high-throughput sequencing analyses	12-15
**Supplementary Table 9**. List of bacterial plasmids used in this work	16-17
**Supplementary Table 10**. List of DNA sequences recognized by TALE proteins	18
**Supplementary Table 11**. Primers used for mammalian and bacteria cell genomic DNA amplification at sites targeted by DdCBEs	19-20

Supplementary Table 12. Sequences of unique molecular identifiers associated with each target plasmid 21

Supplementary Sequences 1. TALE sequences used in DdCBEs 22-27

Supplementary Sequences 2. Sequences of full-length DddA variants 28-29

Supplementary Sequences 3. Sequences used for fluorescence-activated cell sorting of DdCBE-expressing cells 30

Supplementary Note 1. Mitochondrial editing efficiencies of DdCBEs containing a mismatched or non-mismatched terminal TALE repeat 31

Supplementary Note 2. Representative FACS gating plots for eGFP$^+$/mCherry$^+$ cells 32-33

Supplementary Note 3. Nuclear editing efficiencies of DdCBEs containing N-terminal UGI fusions or C-terminal UGI fusions 34

Supplementary References 35

Further evolution of T7-DdCBE-DddA11 for improved GC activity

We previously performed a PANCE of canonical T7-DdCBE using a strains 9 and 10 transformed with MP6. These strains expressed the GCA or GCG linker, respectively (**Extended Data Fig. 7a**). After fifteen passages, the overnight fold phage propagation increased to 1,000, representing >1,000-fold improvement. Clonal sequencing of phage isolated at the end of PANCE, however, revealed a stochastic frameshift mutation within the open reading frame encoding either the left- or right-half of T7-DdCBE (data not shown, available upon request). We speculated that the premature stop codons helped improve phage fitness by reducing the translational burden on the phage, thus increasing phage propagation in a manner that was independent of the deamination activity of DddA. Given that DddA11 already exhibited a broadened targeting scope and non-zero GC activity (**Fig. 6b**), we hypothesized that DddA11 could be a promising evolutionary stepping-stone to serve as a starting target for evolving DddA variants towards higher GC activity.

We initiated PANCE of T7-DdCBE containing DddA11 in duplicates using the same MP6-transformed strains 9 and 10. One replicate in PANCE-GCA and one replicate PANCE-GCG evolved 'cheaters' in which g**III** was recombined into the phage genome. The PANCE schedules shown in **Extended Data Fig. 7d** are for the other replicates that do not contain g**III** within the SP genome. We isolated six to eight plaques from each replicate after round 9 and round 12 for clonal sequencing. The mutation N1378S was strongly enriched in PANCE-GCA and PANCE-GCG. One replicate of PANCE-GCA also showed strong consensus for the additional mutations A1341I and P1394S (**Supplementary Table 7**).

We selected two strongly enriched genotypes (7.9.1 and 7.12.1) and two moderately enriched genotypes (7.12.2 and 7.12.3) for validation of mtDNA base editing activity in human cells (**Extended Data Fig. 8a**). Variant 7.12.1 generally resulted in a 2.2- to 27-fold decline in AC and CC editing compared to DddA11 (**Extended Data Fig. 8b-e**). Variants 7.9.1, 7.12.2, and 7.12.3 improved TC and non-TC editing by 1.4- to 1.6-fold when tested as ND4.3-DdCBE, but did not enhance GC editing compared to DddA11 (**Extended Data Fig. 8b**). ND5.4-DdCBE containing variant 7.9.1 resulted in comparable editing to DddA11 at AC and CC targets (**Extended Data Fig. 8c**). When

tested at other sites, variants 7.9.1, 7.12.2, and 7.12.3 improved TC editing by an average of 1.2-fold compared to DddA11. These variants, however, generally resulted in lower non-TC compared to DddA11 when tested as ND5.2-DdCBE and ATP8-DdCBE (**Extended Data Fig. 8d and 8e**).

Structure alignment of DddA to APOBEC3G

Our previous work identified ssDNA-specific APOBEC3G cytidine deaminase, which has an intrinsic 5'-CC preference[45], as the closest structural relative to DddA. We aligned the catalytic domain of human APOBEC3G complexed with its ssDNA 5'-CCA substrate[46] with DNA-free DddA. The PACE-derived DddA variants DddA8 and DddA11 expanded the putative TC sequence preference to include AC and CC (**Fig. 3b**). These variants contained mutations A1341V, N1342S, G1344R and G1344S that are positioned within a loop that aligns most closely to loop 3 of APOBEC3G (**Extended Data Fig. 10a and 10b**). Previous studies identified DNA-binding loop 3 to be critical for enhancing the catalytic activity of APOBEC3G at 5'CC[47]. In this study, the N1342S nucleotide substitution in DddA11e increased TC editing by 1.3-fold and yielded low but detectable AC and CC editing (**Fig. 3f**). These results suggest that the DddA loop containing N1342 could be engineered to improve the catalytic activity of DddA and support deamination at non-TC contexts.

In APOBEC3G, residue D317 in loop 7 is critical for selectivity towards C_{-1}[47] (**Extended Data Fig. 10b**). Context-specific PANCE of DddA strongly enriched for E1370K across all tested linkers of ACC, CCC and GCC (**Supplementary Table 3**). Given that loop 7 of APOBEC3G spatially aligns with the DddA loop containing E1370K, E1370K could also be involved in altering the substrate selectivity of DddA (**Extended Data Fig. 10b**).

Supplementary Table 1 | Mutation table of variants from PANCE of canonical T7-DdCBE for improved TC activity. Strain 4 transformed with MP6 was infected with input SP encoding the canonical T7-DdCBE (see Extended Data Fig. 1a). Four plaques from each replicate (A, B, C and D) were sequenced after 7 passages. Mutations are highlighted in blue. Genotypes in red were tested for mitochondrial base editing in human cells (see Extended Data Fig. 1d).

	DddA-1397-N											DddA-G1397-C			
Position	1307	1320	1321	1326	1330	1364	1372	1379	1380	1394	1396	1368	1399	1409	1418
wt	Y	D	A	S	S	V	T	M	T	P	E	L	I	T	S
A.1	Y	D	A	S	S	V	I	M	T	P	E	L	I	T	S
A.2	Y	D	A	S	S	V	T	M	I	P	E	L	I	T	S
A.3	Y	D	A	S	I	V	I	M	T	P	E	L	I	T	S
A.4	Y	D	A	S	S	V	I	M	T	P	E	L	I	T	S
B.1	Y	D	A	I	S	V	T	M	T	S	E	L	I	I	R
B.2	Y	N	A	I	S	V	T	M	T	S	E	F	I	I	S
B.3	Y	N	A	S	S	V	T	I	T	P	E	L	I	T	S
B.4	Y	D	T	I	S	V	T	M	T	S	E	L	I	I	S
C.1	Y	D	A	S	S	V	T	M	I	P	E	L	I	T	S
C.2	Y	D	A	S	S	V	T	M	I	P	E	L	I	T	S
C.3	H	D	A	S	S	L	T	M	T	P	E	L	I	T	S
C.4	Y	D	A	S	S	V	T	M	I	P	E	L	S	T	S
D.1	Y	D	A	S	S	V	T	I	T	P	E	L	I	T	S
D.2	Y	D	A	S	S	V	I	M	T	P	E	L	I	T	S
D.3	Y	D	A	S	S	V	T	I	T	P	E	L	I	T	S
D.4	Y	D	A	S	S	V	T	M	I	P	K	L	I	T	S

Supplementary Table 2 | Mutation table of variants from PACE of T7-DdCBE-DddA1 for improved T<u>C</u> activity. Strain 4 transformed with MP6 was infected with SP encoding T7-DdCBE-DddA1 **(**see Extended Data Fig. 1a). Individual plaques were isolated at the end of PACE and sequenced for their DddA genes. Genotypes in red were tested for mitochondrial base editing in human cells (see Fig. 2a).

	DddA -G1397-N									DddA-G1397-C	
**Position**	**1310**	**1314**	**1320**	**1326**	**1330**	**1370**	**1380**	**1389**	**1396**	**1413**	**1417**
**Wildtype**	**Q**	**T**	**D**	**S**	**S**	**E**	**T**	**K**	**E**	**T**	**N**
**T7-DdCBE-DddA1**	**Q**	**T**	**D**	**S**	**S**	**E**	I	**K**	**E**	**T**	**N**
**A.1**	**Q**	**T**	**D**	I	**S**	**E**	I	**K**	K	**T**	**N**
**A.2**	**Q**	**T**	**D**	**S**	**S**	**E**	I	**K**	K	**T**	**N**
**A.3**	**Q**	A	**D**	**S**	**S**	**E**	I	**K**	K	**T**	**N**
**A.4**	**Q**	A	**D**	**S**	**S**	G	I	**K**	K	**T**	**N**
**A.5**	**Q**	A	**D**	**S**	**S**	**E**	I	**K**	K	**T**	**N**
**A.6**	**Q**	A	**D**	**S**	**S**	**E**	I	**K**	K	**T**	**N**
**A.7**	**Q**	A	**D**	**S**	**S**	**E**	I	**K**	K	**T**	**N**
**B.1**	**Q**	**T**	**D**	**S**	**S**	**E**	I	**K**	**E**	**T**	**N**
**B.2**	**Q**	**T**	**D**	**S**	**S**	**E**	I	**K**	**E**	**T**	**N**
**B.3**	**Q**	A	**D**	**S**	**S**	**E**	I	**K**	**E**	**T**	**N**
**B.4**	**Q**	A	**D**	**S**	**S**	**E**	I	**K**	**E**	**T**	**N**
**B.5**	**Q**	**T**	**D**	**S**	**S**	**E**	I	**K**	**E**	**T**	**N**
**B.6**	**Q**	**T**	**D**	**S**	**S**	**E**	I	**K**	**E**	**T**	**N**
**B.7**	**Q**	**T**	**D**	**S**	**S**	**E**	I	**K**	**E**	**T**	**N**
**B.8**	**Q**	**T**	**D**	**S**	**S**	**E**	I	**K**	**E**	**T**	**N**
**C.1**	**Q**	**T**	**D**	**S**	**S**	**E**	I	**K**	**E**	I	**N**
**C.2**	**Q**	**T**	**D**	**S**	**S**	**E**	I	Q	**E**	I	**N**
**C.3**	**Q**	**T**	**D**	**S**	**S**	**E**	I	**K**	**E**	I	**N**
**C.4**	**Q**	**T**	**D**	**S**	**S**	**E**	I	**K**	**E**	**T**	**N**
**C.5**	**Q**	**T**	**D**	**S**	**S**	**E**	I	**K**	**E**	**T**	**N**
**C.6**	**Q**	**T**	**D**	**S**	I	**E**	I	**K**	G	**T**	**N**
**C.7**	**Q**	**T**	**D**	**S**	**S**	**E**	I	**K**	**E**	I	**N**
**D.2**	R	**T**	**D**	**S**	I	**E**	I	**K**	**E**	**T**	**N**
**D.3**	R	**T**	**D**	**S**	I	**E**	I	**K**	**E**	**T**	**N**
**D.5**	R	**T**	**D**	**S**	I	**E**	I	**K**	**E**	**T**	K
**D.6**	**Q**	**T**	N	**S**	I	**E**	I	**K**	**E**	**T**	**N**
**D.7**	**Q**	A	**D**	**S**	**S**	**E**	I	**K**	**E**	**T**	**N**
**D.8**	R	**T**	**D**	**S**	I	**E**	I	**K**	**E**	**T**	**N**

Supplementary Table 3 | Mutation table of variants from PANCE of T7-DdCBE-DddA1 for expanded targeting scope. Strains 5, 6 or 7, which were each transformed with MP6, were used for PANCE-ACC, PANCE-CCC or PANCE-GCC, respectively (see Extended Data Fig. 3a for strain identities). Each host strain was infected with input SP encoding T7-DdCBE-DddA1. Plaques from each replicate (A, B, C and D) were sequenced after 9 passages. Mutations are highlighted in blue. Phage lagoons highlighted in red were used as inputs for PACE.

	DddA-G1397-N																		DddA-G1397-C					
Position	1290	1299	1300	1301	1310	1314	1320	1321	1326	1328	1330	1334	1341	1342	1344	1364	1370	1380	1398	1406	1413	1421	1425	
Wildtype	G	I	S	A	Q	T	D	A	S	V	S	P	A	N	G	V	E	T	A	T	T	S	G	
T7-DdCBE-DddA1	G	I	S	A	Q	T	D	A	S	V	S	P	A	N	G	V	E	I	A	T	T	S	G	
ACC-A.8	G	I	S	A	Q	A	D	A	S	V	S	P	A	N	G	V	K	I	A	T	T	S	G	PANCE-ACC
ACC-B.1	G	I	S	A	Q	T	D	A	S	V	S	P	A	S	G	V	K	I	A	T	T	S	G	
ACC-B.2	G	I	S	A	Q	T	D	A	S	V	S	P	A	S	G	V	K	I	A	T	T	S	G	
ACC-B.3	G	I	S	A	Q	T	D	A	S	V	S	P	A	N	G	V	K	I	A	T	T	S	G	
ACC-B.4	G	I	S	A	Q	T	D	A	S	V	S	P	A	S	G	V	K	I	A	T	T	S	G	
ACC-B.6	G	I	S	A	Q	T	D	A	S	V	S	P	A	S	G	V	K	I	A	T	T	S	G	
ACC-B.7	G	I	S	A	Q	I	D	A	S	V	S	P	A	S	G	V	K	I	A	T	T	S	G	
ACC-C.1	G	I	S	A	Q	T	D	A	S	V	S	P	A	N	G	V	K	I	A	T	T	S	G	
ACC-C.2	G	I	S	A	Q	A	D	A	S	V	S	P	A	N	G	V	K	I	A	T	T	S	G	
ACC-C.3	G	I	S	A	Q	A	D	A	S	V	S	P	A	N	G	V	K	I	A	T	T	S	G	
ACC-C.4	G	I	S	A	Q	A	D	A	S	V	S	P	A	N	G	V	K	I	A	T	T	S	G	
ACC-C.5	G	V	S	A	Q	A	D	A	S	V	S	P	A	N	G	V	K	I	A	T	T	S	G	
ACC-C.6	G	I	S	A	Q	A	D	A	S	V	S	P	A	N	G	V	K	I	A	T	T	S	G	
ACC-C.8	G	I	S	A	Q	A	D	A	S	V	S	P	A	N	G	V	K	I	A	T	T	S	G	
ACC-D.1	G	I	S	A	Q	T	D	A	S	V	I	P	A	N	G	V	K	I	A	T	T	S	G	
ACC-D.2	G	I	S	A	Q	T	D	A	S	V	I	P	A	N	G	V	K	I	A	T	T	S	G	
ACC-D.3	G	I	S	A	Q	T	D	A	S	V	S	P	A	N	G	V	K	I	A	T	T	S	G	
ACC-D.4	G	I	S	A	Q	T	D	A	S	V	S	P	A	N	G	V	K	I	A	T	T	S	G	
ACC-D.5	G	I	S	A	Q	T	D	A	S	V	S	P	A	N	G	V	K	I	A	T	T	S	G	
ACC-D.6	G	I	S	A	Q	T	D	A	S	V	S	P	A	N	G	V	K	I	A	T	T	S	G	
ACC-D.7	G	I	S	A	Q	T	D	A	S	V	S	P	A	N	G	V	K	I	A	T	T	S	G	
ACC-D.8	G	I	S	A	Q	T	D	A	S	V	S	P	A	N	G	V	K	I	A	T	T	S	G	
CCC-A.1	G	I	S	A	Q	T	D	A	S	V	S	P	A	S	G	V	K	I	A	T	T	S	G	PANCE-CCC
CCC-A.2	G	I	S	A	Q	T	D	A	S	V	S	P	A	S	G	V	K	I	A	T	T	I	G	
CCC-A.3	G	I	S	A	Q	T	D	A	S	V	S	P	A	S	G	V	K	I	A	T	T	S	G	
CCC-A.4	G	I	S	A	Q	T	D	A	S	V	S	S	A	S	G	V	K	I	A	T	T	S	G	
CCC-A.5	G	I	S	A	Q	T	D	A	S	V	S	P	A	S	G	V	K	I	A	T	T	S	G	
CCC-A.6	G	I	S	A	Q	T	D	A	S	V	S	P	A	S	G	V	K	I	A	T	T	S	G	
CCC-A.7	G	I	S	A	Q	T	D	A	S	V	S	P	A	S	G	V	K	I	A	T	T	S	G	
CCC-A.8	G	I	S	A	Q	T	D	A	S	V	S	P	A	S	G	V	K	I	A	T	T	S	G	
CCC-B.1	G	I	S	A	Q	T	D	S	S	V	S	P	A	S	G	V	K	I	A	A	T	S	G	
CCC-B.2	G	I	S	A	Q	T	D	A	S	V	S	P	A	N	R	M	K	I	A	T	T	S	G	
CCC-B.3	G	I	S	A	Q	T	D	A	S	V	S	P	A	N	R	M	K	I	A	T	T	S	G	
CCC-B.4	G	I	S	A	Q	T	D	A	S	V	S	P	A	N	R	M	K	I	A	T	T	S	G	
CCC-B.5	G	I	S	A	Q	T	D	A	S	V	S	P	A	N	G	V	K	I	A	T	T	S	G	
CCC-B.6	G	I	S	A	Q	T	D	A	S	V	S	P	A	N	R	M	K	I	A	T	T	S	G	
CCC-B.7	G	I	S	A	Q	T	D	A	S	V	S	P	A	N	R	M	K	I	A	T	T	S	G	
CCC-B.8	G	I	S	A	Q	T	D	A	S	V	S	P	A	N	G	V	K	I	A	T	T	S	G	
CCC-C.1	G	I	S	A	Q	T	D	A	S	V	S	P	T	S	G	V	K	I	A	T	T	S	G	
CCC-C.2	G	I	S	A	Q	T	D	A	S	V	S	P	T	S	G	V	K	I	A	T	T	S	G	
CCC-C.3	G	I	S	A	Q	T	D	A	S	V	S	P	T	S	G	V	K	I	A	T	T	S	G	
CCC-C.4	G	I	S	A	K	T	D	A	S	V	I	P	A	N	G	V	K	I	A	T	T	S	G	
CCC-C.5	G	I	S	A	Q	T	D	A	S	V	S	P	T	S	G	V	K	I	A	T	T	S	G	
CCC-C.6	G	I	S	A	Q	T	D	A	S	V	S	P	T	S	G	V	K	I	A	T	T	S	G	
CCC-C.7	G	I	S	A	Q	T	D	A	S	V	S	P	T	S	G	V	K	I	A	T	T	S	G	
CCC-C.8	G	I	S	A	Q	T	D	A	S	V	S	P	T	S	G	V	K	I	A	T	T	S	G	
CCC-D.1	G	I	S	A	Q	T	D	A	S	V	S	P	V	N	G	V	K	I	A	T	T	S	G	
CCC-D.3	G	I	S	A	Q	T	D	A	S	M	S	P	V	S	G	V	K	I	A	T	T	S	G	
CCC-D.4	G	I	S	A	Q	T	D	A	S	V	S	P	V	S	G	V	K	I	A	T	T	S	V	
CCC-D.5	G	I	S	A	Q	T	D	A	S	V	S	P	V	S	G	V	E	I	A	T	T	S	G	
CCC-D.6	G	I	S	A	Q	T	D	A	S	V	S	P	V	S	G	V	K	I	A	T	T	S	G	
CCC-D.7	G	I	S	A	Q	T	D	A	S	V	S	P	V	S	G	V	K	I	A	T	T	S	G	
CCC-D.8	G	I	S	A	Q	T	D	A	S	V	S	P	V	S	G	V	K	I	A	T	T	S	G	
GCC-A.1	G	I	S	A	Q	T	D	A	S	V	I	P	V	N	G	V	K	I	A	T	T	S	G	PANCE-GCC
GCC-A.2	G	I	S	A	Q	T	D	A	S	V	I	P	V	S	G	V	K	I	A	T	T	S	G	
GCC-A.3	G	I	S	A	Q	T	D	A	S	V	I	P	V	S	G	V	K	I	A	T	T	S	G	
GCC-A.4	G	I	S	A	Q	T	D	A	S	V	S	P	V	S	G	V	K	I	A	T	T	S	G	
GCC-A.5	G	I	S	A	Q	T	D	A	S	V	I	P	V	S	G	V	K	I	A	T	T	S	G	
GCC-A.6	G	I	S	A	Q	T	D	A	S	V	S	P	V	S	G	V	K	I	A	T	T	S	G	
GCC-A.7	G	I	S	A	Q	T	D	A	S	V	S	P	V	N	G	V	K	I	A	T	T	S	G	
GCC-A.8	G	I	S	A	Q	T	N	A	S	V	I	P	V	S	G	V	K	I	A	T	T	S	G	
GCC-B.1	G	I	S	T	Q	T	D	A	N	V	S	P	A	N	S	V	K	I	A	T	T	S	G	
GCC-B.2	G	I	S	A	Q	T	D	A	S	V	S	P	A	N	S	V	K	I	A	T	T	S	G	
GCC-B.3	G	I	S	A	Q	T	D	A	S	V	S	P	A	N	S	V	K	I	A	T	T	S	G	
GCC-B.4	G	I	S	A	Q	T	D	A	S	V	S	P	A	N	S	V	K	I	A	T	I	S	G	
GCC-B.5	G	I	S	A	Q	V	D	A	S	V	S	P	A	N	S	V	K	I	A	T	T	S	G	
GCC-B.6	G	I	S	A	Q	T	N	A	S	V	S	P	A	N	S	V	K	I	A	T	T	S	G	
GCC-B.7	G	I	S	A	Q	A	D	A	S	V	S	P	A	N	S	V	K	I	A	T	T	S	G	
GCC-B.8	G	I	S	A	Q	T	D	A	S	V	S	P	A	N	S	V	K	I	A	T	T	S	G	
GCC-C.1	G	I	S	A	Q	T	D	S	S	V	S	P	A	N	G	V	K	I	A	A	T	S	G	
GCC-C.2	G	I	S	A	Q	T	D	A	S	V	S	P	A	N	S	V	K	I	A	T	T	S	G	
GCC-C.3	G	I	S	A	Q	T	D	A	S	V	S	P	A	N	S	V	K	I	A	T	T	S	G	
GCC-C.4	G	I	S	A	Q	T	D	A	S	V	S	P	A	N	S	V	K	I	A	T	T	S	G	
GCC-C.5	G	I	S	A	Q	T	D	A	S	V	S	P	A	N	S	V	K	I	A	T	T	S	G	
GCC-C.6	G	I	S	A	Q	T	D	A	S	V	S	P	A	N	S	V	K	I	A	T	T	S	G	
GCC-C.7	G	I	S	A	Q	T	D	A	S	V	S	P	A	N	S	V	K	I	A	T	T	S	G	
GCC-C.8	G	I	R	A	Q	T	D	A	S	V	S	P	A	N	S	V	K	I	A	T	T	S	G	
GCC-D.1	G	I	S	A	Q	T	D	A	S	V	S	P	T	S	G	V	K	I	A	T	T	S	G	
GCC-D.2	G	I	S	A	Q	T	D	T	S	V	S	P	A	N	S	V	K	I	T	T	T	S	G	
GCC-D.3	G	I	S	A	Q	T	D	A	S	V	S	P	A	N	S	V	K	I	T	T	T	S	G	
GCC-D.4	G	I	S	A	Q	T	D	A	S	V	S	P	A	N	S	V	K	I	A	T	T	S	G	
GCC-D.5	V	I	S	A	Q	T	N	A	S	V	S	P	A	N	S	V	K	I	A	T	T	S	G	
GCC-D.6	G	I	S	A	Q	T	D	A	S	V	S	P	A	N	S	V	K	I	T	T	T	S	G	
GCC-D.7	G	I	S	A	Q	T	D	A	S	V	S	P	A	N	S	V	K	I	A	T	T	S	G	
GCC-D.8	G	I	S	A	Q	T	D	A	S	V	S	P	A	N	S	V	K	I	A	T	T	S	G	

Supplementary Table 4 | Mutation table of variants from the PACE evolution to expand targeting scope. Host strain 6 transformed with MP6 was infected with the phage population CCC-B from PANCE. Host strain 7 transformed with MP6 was infected either phage population GCC-A or GCC-D from, both of which were derived from PANCE (see Extended Data Fig. 3a for strain identities). The consensus genotypes of input phage populations from PANCE are shown. Data was obtained by sequencing individual plaques isolated at the end of PACE. Genotypes in red were tested for base editing in mammalian cells (see Fig. 3c). †T1413I was included in this genotype.

		DddA-G1397N																DddA-G1397-C					
	Position	1301	1314	1321	1323	1325	1326	1329	1330	1341	1342	1344	1364	1370	1377	1380	1396	1398	1404	1407	1410	1413	1417
	Wild-type	A	T	A	G	E	S	F	S	A	N	G	V	E	V	T	E	A	G	G	K	T	N
PANCE	CCC-B	A	T	A	G	E	S	F	S	A	N	R	M	K	V	I	E	A	G	G	K	T	N
PACE	CCC-B.1	A	A	A	G	E	S	F	S	A	N	R	M	K	V	I	E	A	G	G	K	I	N
	CCC-B.2	A	T	A	G	A	S	F	S	A	S	R	M	K	V	I	E	A	G	G	K	I	N
	CCC-B.3	A	A	E	S	E	S	F	S	A	N	R	M	K	V	I	E	A	G	G	K	T	N
	CCC-B.4	A	T	A	G	E	S	F	S	A	S	R	M	K	V	I	E	A	G	G	K	I	N
	CCC-B.5	A	T	A	G	E	S	F	S	A	N	G	V		V		E	A	G	D	K	I	N
	CCC-B.6	S	T	A	G	E	S	F	S	A	N	R	M	K	V	I	E	A	G	G	K	I	S
PANCE	GCC-A	A	T	A	G	E	S	F	I	V	S	G	V	K	V	I	E	A	G	G	K	T	N
PACE	GCC-A.1†	A	T	A	G	E	S	F	I	V	S	G	V	K	V	I	E	A	G	G	K	T	N
	GCC-A.2	A	T	E	G	E	S	F	I	V	S	G	V	K	V	I	E	A	G	G	K	T	N
	GCC-A.3	A	T	A	G	E	S	F	I	V	S	G	V	K	V	I	E	A	G	G	K	T	N
	GCC-A.4	A	T	A	G	E	S	F	I	V	S	G	V	K	V	I	E	A	G	G	K	T	N
	GCC-A.5	A	T	A	G	E	S	F	I	V	S	G	V	K	V	I	E	A	G	G	K	T	N
	GCC-A.6	A	T	A	G	E	S	F	I	V	S	G	V	K	V	I	E	A	G	G	K	T	N
	GCC-A.7	A	T	A	G	E	S	F	I	V	S	G	V	K	V	I	E	A	G	G	K	T	N
	GCC-A.8	A	T	A	G	E	S	F	I	V	S	G	V	K	M	I	E	A	G	G	K	T	N
PANCE	GCC-D	A	T	A	G	E	S	F	S	A	N	S	V	K	V	I	E	T	G	G	K	T	N
PACE	GCC-D.1	A	A	A	G	E	S	F	S	A	N	S	V	K	V	I	G	T	G	G	R	T	N
	GCC-D.2	A	A	A	G	E	S	F	S	A	N	S	V	K	V	I	E	A	G	G	K	T	N
	GCC-D.3	A	A	A	G	E	S	F	S	A	N	S	V	K	V	I	E	A	G	G	K	T	N
	GCC-D.4	A	A	A	G	E	S	F	S	A	N	S	V	K	V	I	E	T	G	G	K	T	N
	GCC-D.5	A	A	A	G	E	S	F	S	A	N	S	V	K	V	I	K	T	G	G	K	I	N
	GCC-D.6	A	A	A	G	E	S	F	S	A	N	S	V	K	V	I	E	T	G	G	K	I	N
	GCC-D.7	A	T	A	G	E	S	F	S	A	N	S	V	K	V	I	E	T	G	G	K	I	N
	GCC-D.8	A	T	A	G	E	V	L	S	A	N	S	V	K	V	I	K	T	G	G	K	I	N

(See the separately provided Excel file.)

Supplementary Table 5 | Sequencing coverage for ATAC-seq samples. Sequencing coverages for all replicates in Fig. 3g are provided.

(See the separately provided Excel file.)

Supplementary Table 6 | List of predicted off-target nuclear DNA sites for SIRT6-DdCBE and JAK2-DdCBE. TALE off-targets were predicted using the online PROGNOS tool. Off-target sites were ranked based on the RVD nucleotide preferences observed in natural TAL effectors.

Supplementary Table 7 | Mutation table of variants from the GC-specific PANCE. Strain 9 transformed with MP6 was used for PANCE-GCA. Strain 10 transformed with MP6 was used for PANCE-GCG (see Extended Data Fig. 7b for strain identities). Each host strain was infected with input SP encoding the DddA11 variant of T7-DdCBE. Plaques were sequenced after nine and 12 passages. Mutations are highlighted in blue.

		G1397-N																	DddA-C			
		1290	1296	T1311	1320	1325	1326	1330	1334	P1336	1341	1342	1349	1351	1370	1378	1380	1394	1413	1414	1421	
	Wild-type	G	P	T	D	E	S	S	P	P	A	N	Q	A	E	N	T	P	T	G	S	
	DddA11	G	P	T	D	E	S	I	P	P	V	S	Q	A	K	N	I	P	I	G	S	
Round 9	GCA.1	G	P	T	D	E	S	I	P	P	V	S	Q	A	K	N	I	P	I	G	S	PANCE-GCA
	GCA.2	G	P	T	D	E	S	I	P	P	V	S	Q	A	K	N	I	P	I	G	S	
	GCA.3	G	P	T	D	E	S	I	P	P	A	S	Q	T	K	N	I	P	I	G	S	
	GCA.4	G	P	T	D	E	S	I	P	P	V	S	Q	A	K	N	I	P	I	S	S	
	GCA.5	G	T	T	D	E	S	I	P	P	V	S	Q	A	K	N	I	P	I	G	S	
	GCA.6	V	P	T	D	E	S	I	P	P	V	S	Q	A	K	N	I	P	I	G	S	
	GCA.7	G	P	T	D	E	S	I	P	P	V	S	Q	A	K	N	I	P	I	G	S	
	GCA.8	G	P	T	D	E	S	I	P	P	V	S	Q	A	K	N	I	P	I	G	S	
	GCG.1	G	P	A	D	K	S	I	P	P	V	S	Q	A	K	S	I	P	I	G	S	PANCE-GCG
	GCG.2	G	P	T	D	K	S	I	P	P	V	S	Q	A	K	S	I	P	I	G	S	
	GCG.3	G	P	T	D	E	I	I	P	P	V	S	Q	A	K	S	I	P	I	G	S	
	GCG.4	G	P	T	D	E	S	I	P	P	V	S	Q	A	K	S	I	P	I	G	S	
	GCG.5	G	P	T	D	E	S	I	P	P	V	S	Q	A	K	S	I	P	I	G	S	
	GCG.6	G	P	T	D	E	S	I	P	P	V	S	Q	A	K	S	I	P	I	G	S	
	GCG.7	G	P	T	D	K	S	I	P	P	V	S	Q	A	K	S	I	P	I	G	S	
	GCG.8	G	P	T	D	E	S	I	S	P	V	S	Q	A	K	S	I	P	I	G	S	
Round 12	GCA.1	G	P	T	D	K	S	I	P	P	V	S	Q	A	K	N	I	P	I	G	S	PANCE-GCA
	GCA.2	G	P	T	N	E	S	I	P	P	I	S	Q	A	K	S	I	S	I	G	S	
	GCA.3	G	P	T	D	E	S	I	P	P	I	S	Q	A	K	S	I	S	I	G	S	
	GCA.4	G	P	T	D	E	S	I	P	P	I	S	Q	A	K	S	I	S	I	G	S	
	GCA.5	G	P	T	D	E	S	I	P	P	I	S	Q	A	K	S	I	S	I	G	S	
	GCA.6	G	P	T	D	E	S	I	P	P	I	S	H	A	K	S	I	S	I	G	S	
	GCG.1	G	P	T	D	K	S	I	P	P	V	S	Q	A	K	S	I	P	I	G	S	PANCE-GCG
	GCG.2	G	P	T	D	E	S	I	S	S	V	S	Q	A	K	S	I	P	I	G	S	
	GCG.3	G	P	T	D	E	S	I	P	P	V	S	Q	A	K	S	I	P	I	G	S	
	GCG.4	G	P	T	D	E	S	I	P	P	V	S	Q	A	K	S	I	P	I	G	S	
	GCG.5	G	P	T	D	K	S	I	P	P	V	S	Q	A	K	S	I	P	I	G	R	
	GCG.6	G	P	T	D	E	S	I	S	P	V	S	Q	A	K	S	I	P	I	G	S	

Supplementary Table 8 | Amplicons for high-throughput sequencing analyses

Site	Amplicon
ND1	CTCACCATCGCTCTTCTACTATGAACCCCCCTCCCCATACCCAACCCCCTGGTC<br>AACCTCAACCTAGGCCTCCTATTTATTCTAGCCACCTCTAGCCTAGCCGTTTACT<br>CAATCCTCTGATCAGGGTGAGCATCAAACTCAAACTACGCCCTGATCGGCGCAC<br>TGCGAGCAGTAGCCCAAACAATCTCATATGAAGTCACCCTAGCC
ND1.2	GCCAACCTCCTACTCCTCATTGTACCCATTCTAATCGCAATGGCATTCCTAATGC<br>TTACCGAACGAAAAATTCTAGGCTATATACAACTACGCAAAGGCCCCAACGTTGT<br>AGGCCCCTACGGGCTACTACAACCCTTCGCTGACGCCATAAAACTCTTCACCAA<br>AGAGCCCCTAAAACCCGCCACATCTACCATCACCCTCTACATCACCGCCC
ND2	CGTAAGCCTTCTCCTCACTCTCTCAATCTTATCCATCATAGCAGGCAGTTGAGGT<br>GGATTAAACCAAACCCAGCTACGCAAAATCTTAGCATACTCCTCAATTACCCACA<br>TAGGATGAATAATAGCAGTTCTACCGTACAACCCTAACATAACCATTCTTAATTTA<br>ACTATTTATATTATCCTAACTACTACCGCATTCCTACTACTCAAC
ND4	GACTTCAAACTCTACTCCCACTAATAGCTTTTTGATGACTTCTAGCAAGCCTCGC<br>TAACCTCGCCTTACCCCCCACTATTAACCTACTGGGAGAACTCTCTGTGCTAGTA<br>ACCACGTTCTCCTGATCAAATATCACTCTCCTACTTACAGGACTCAACATACTAG<br>TCACAGCCCTATACTCCCTCTACATATTTACCACAAC
ND4.2<br>ND4.3	CTGCCTACGACAAACAGACCTAAAATCGCTCATTGCATACTCTTCAATCAGCCAC<br>ATAGCCCTCGTAGTAACAGCCATTCTCATCCAAACCCCCTGAAGCTTCACCGGC<br>GCAGTCATTCTCATAATCGCCCACGGACTTACATCCTCATTACTATTCTGCCTAG<br>CAAACTCAAACTACGAACGCACTCACAGTCGCATCATAATCCTCTCTCAAGGACT<br>TCAAACTCTACTCCCA
ND5.2	CGGGTCCATCATCCACAACCTTAACAATGAACAAGATATTCGAAAAATAGGAGG<br>ACTACTCAAAACCATACCTCTCACTTCAACCTCCCTCACCATTGGCAGCCTAGCA<br>TTAGCAGGAATACCTTTCCTCACAGGTTTCTACTCCAAAGACCACATCATCGAAA<br>CCGCAAACATATCATACACAAACGCCTGAGCCCTATCTATTACTCT
ND5.4	GCAGTCTGCGCCCTTACACAAAATGACATCAAAAAAATCGTAGCCTTCTCCACTT<br>CAAGTCAACTAGGACTCATAATAGTTACAATCGGCATCAACCAACCACACCTAGC<br>ATTCCTGCACATCTGTACCCACGCCTTCTTCAAAGCCATACTATTTATGTGCTCC<br>GGGTCCATCATCCACAACCTTAACAATGAACAAGATATTCG
ATP8	CTTTACAGTGAAATGCCCCAACTAAATACTACCGTATGGCCCACCATAATTACCC<br>CCATACTCCTTACACTATTCCTCATCACCCAACTAAAAATATTAAACACAAACTAC<br>CACCTACCTCCCTCACCAAAGCCCATAAAAATAAAAAATTATAACAAACCCTGAG<br>AACCAAAATGAACGAAAATCTGTTCGCTTCATTCATTGCCCCC
SIRT6 (on-target)	GGAAGCGGCCTCAACAAGGGAAACTTTATTGTTCCCGTGGGGCAGTCGAGGAT<br>GTCGGTGAATTACGCGGCGGGGCTGTCGCCGTACGCGGACAAGGGCAAGTGC<br>GGCCTCCCGGAGGTGAGCGCGTCTGAGGGTCCCGAGCATCGCGCCCCAGGGC<br>CGCGGGCTGGGAGCGGCCGGATGGCAGGGGGGCATTGTGGGAATATCAGGG<br>GGC
JAK2 (on-target)	GCTAGGATTACAGGTGTGAGACACTGCGCCCAGCCCATTTGTAACTTTATTGTTT<br>TCTCTTACAGGCAAATGTTCTGAAAAAGACTCTGCATGGGAATGGCCTGCCTTA<br>CGATGACAGAAATGGAGGGAACATCCACCTCTTCTATATATCAGAATGGTGATAT<br>TTCTGGAAATGCCAATTCTATGAAGCAAATAGATCCAGTTCTTCAGGTG
pBM10 plasmid series for NCN library	CGTTTTAGACTGAGCACGTCAATACGCAAACCGCCTCTCCCCGCGCGTTGGCC<br>GATTCATTAATGCAGCTGGCACGACAGGTTTCCCGACTGGAGTTTGTAGTCTAG<br>TCTGT**CCGACTTCGCGTT**TTTTT<u>TGT</u>TTATTTC**AGCAAACG**ACGAACCCGCCAAA<br>CGCGCCCTGACGGGATTGTCTGCTCCCGGCATCCG<br>Blue = UMI; Bold = TALE binding sites; Underlined = NCN target

SIRT6-OT1	TGGCCAAGCAGGGATCACGTCCCTCTGCCTTGGCCACTCCAGGCCACCTGCAG AGGGGGCAGCCCTGTCAGTGATGGAGGGGGAGACAGCCTGTGTTATTCACGCC GGAAGTCCATGGCATTCTCAGAGAGCTCTTCCCCGTACCTCCCCAGAACACTGA GAATGGATGAATCAGTCCCGGCTCATCAGAGCCTTTCATGCTAAACCAGCCCAA ATGACTGATTTAGAAACTGCGTGGTCTTGTTCTTTGAGTGGATTTCTGAGGCACA CTCCAGAGCAGGCCCAGGTCTTCATTATCTCCATGCACACCCTTTGATTAAGCAT CTGTGACCACACAGCACTGTGTGCTGTACCCAAGGAGGCAACAG
SIRT6-OT2	GGGGGAGTCGGACTTAGAAGGTTGCCTCTGCTGCTCTCCCACTAAGAACAATG CAGCTGCCCAGGAAACACACCCGAGCTATGAATAGGCTCAAGCACACAGGGCG AAGCTGTCGCATTTCTGCCGTTTAGAAAGGATCTCTAGTGTGAAGTTGCTTGGA GGCTCTGATTTTGCAAACCCAATTAAATATAGATTTCTTTGGGGTGACGGTTGGG AAGGGTGTGGGTGGAATGTATGTCTGTGCTGCAATGCAATTTAATCCTGAAAGT TTCAGCAAACCTATTCACACACTCCATAAACATTTCTGAGCACCTGCTGTGTGCC AAG
SIRT6-OT3	TGCTAGGCCCCGATTTGTGGACACTGAGCTAGGCAAAAGGATATATAAAGAGAT GCAAGCTAGAGGAGGGGAAAGTTCTGGATGGGGACCTCATTGGCCTCCTCACC TCTGTAGACCCACGTCTTGGGTTGACCCAGGGTAGAACCCTATAAGGATTTGTT CAATAAACGAATGCATAGAGACTGATCAACAACTGACAAAGTGGTATTTTACAGA TGCCGTGAAAGCAGCATACAAGCCGGAGAGCCTACTGGAAGCAAGAGGGGAAA ACCCAGCCGGGTATGGGGGCTGCGGGGAGGATGCCAGGTGGCGGAGACTGGA TTCAGGATTTGATGCTTGAACTGAACCTTCTTTTAAGAGGTGAGTGGGCTGGGA GGTTGTGATGCCCAGAGGGTGAGGAAGGAAGTGGGG
SIRT6-OT4	GACCCTTCCATTAGATCTGAGGGCGGGGTGGGAGGGCAGAGGAGGGGAGGAC AGAGAAGTGGGAGGAAGCAGGAGAGGGGGTGGAGGGGTAGGGGCACAGTGTC ATGCCTCTCGGGCTTCCCCCACAGGGTGAATGACTGTGTGCTGCGGGTGAATG AGGTGGACGTGTCGGAGGTGGTACACAGCCGGGCGGTGGAGGCGCTGAAGGA GGCAGGCCCTGTGGTGCGATTGGTGGTGCGGAGGCGACAGCCTCCACCCGAG ACCATCATGGAGGTCAACCTGCTCAAAGGGCCCAAAGGTGCGGCCCTCCAGGT TCCTGTGCTCCAGCCAGAGC
SIRT6-OT5	GGATCCAAGGTGCCTTCACCCTGCGGCCTGCTCTATGAGGGCCTGCGTCGCAC ATCAGCTGTTGGTATCCTCACCTCGCTGAGCCGTTCCTCCGAGTGTGCTCTGTG GCTTGCACCAGGCCTAGCTGGGCCTTGTGTCTGGTCTCATCCAGCGTCTTCTGA TTGTGGGGAATGAGGCAGAGGTCTCCCCACCTGGGCTGTCCGTGTGGCGAAAG CCTTCCGGGGGGAGGGAGAGGAAGTGCCTCTACAAGGGCCTAGAGTCGTCCT GTGGCACTGAGGGCTCTGGCAGCTGCACACAGGTTGATAATGTCACTGGTGGG TGAGCTCTGTGGT
SIRT6-OT6	GCTGGATTCGATCTGAGGTCAGCTTGACTTTTCCTTCAGGATGATGCGCTGCTT CCTCTGATGAAGGATCAGGGATGCAGAATGGGACTGATGTATCTGTCCAATCAT CCCACTCATTCTCCTCACTTTGCATGCTCTTCTCGGGGCCATCTACAGACAGGT GAGGGGCCCCAGGCAGACAGCTCCTTGGGACACTGAGCTCCACATTGAAATGT TACTCAAATGAATGCGCAGAGGAGCTGTGAGTGAGAAAGTAAGCCCTCCAGCGT GAAGAGCCCCGGAAAAGCCACCCTTGCTCTGGCACCTGTCATGCAGCCCTCAC AGCAGTACTCCCATGGGGTATGGGAGGACAGGGGCACTTCTGTTGGTCTTTCCT TCCTCATGTTCGTCCCAGTAGGGCCTG
SIRT6-OT7	CCCAGGACAAAGTTGCTTTGGGGCGCTTACAGGTTGAATCACCCATAGCTATGT TTTGTTTGGCTTGTACTGTGTTGTTTACATTATTTTATTAGTTGGCAATAGCTTTAA ATCAAAATTTACATTAAGGTCCAGATTTGGGGCTCCTCTTAAAAAAATAAATCAG CACATCTACAACAGTAGGATTTTCACGCCCAATGGCAAAATCTGTTAAAGCTGTG

	TGACAGCTTCTCCTTTTAATGGAGATGACCTCTTCAGACCCTGTACTCCAGGTGC CACAGGAACCAGCTGCCCGGTTCATCCTTGTGTGTTACCTGCATGAGGTGGCAA CCTTGTGCAAGAG
SIRT6-OT8	GCCTCGCCAAGAAACGCCCATTTATTTCCTTCCTATTTATTTATTTTGCAAAAGCC GCCTTTTGAAAGCCGGAGTGCAGTGCGGACTGGCTGAGGCCGGGGCCCAGGC GCGCGCCTCCTTCCTGCAGGCGGCCCGGGGCATCGATCGCGCGGGGCGTAAT GAACCCTAATAACAGCTCTATCACCGCCCGCCCGCCAATTGGCCCGGGTGCCC TCCAAATTAGCCACAAAGAAGCCAGTCTGTCAATATTAATTCCGCCGCGGAGATT ACTTGTTGTGGGAGAACAAAGGTGTTCTGGCTTGAGCTGAACAACTTAACTCCTT GTCTCATAAAATTTAAGCTGCTATTGATCGCCTCCTTGCATTGTGTGAGGATTAA ACACGTGCGCGTGCACATCCAGGCACACGCGCGCACACACTCGGGGCGCCCG GCCCCGCTCAGGCAGCCGCCCCACCTCCTCGCCTACCCGGAAT
SIRT6-OT9	CCAGCACACCCTGATAAGCAGGATTCAGATTGGGCATGGGACAGGACAAAGGC TCTGAGGAGGCATGAGTGGAGTAGTAGAGAGAACACAGGACTCTGGGTTCTGA GCCAAGCTCTGCCATCCTGCAGCCATCTGACCCCTGGCCAGGCCTTCCCCCAA CTTCCTCAGCTGTGAAACGCGGCAGGGCTGCAGATGACGTAGGTGGGCAGAGG CCCTCTGGTGCCTACTGCTAGACACCCTTCTACTCTGGTGGGAGACCTTGTGCT CAAACGCTCTCAGAAGTCAGGGCAGTTGGGAGTCCAGGTCACACACACTCACT GGGGCCCT
SIRT6-OT10	CCAGGGCAACAGGTTTAAGACCCATTAGGAGACAATATTGTCGTCATGAATATAA ATGAGGCCTAGGCAGGTTGGGAGTAGCTAGTGTCTTGGCTCTGGCACAACCAG GCCCTGTTCCTCCACAGGGCTGGCCAGGGCAGAGGTGAAAGGGGCTGGGGTG TGACCGAGTGAGCTGGGGTAGAGGTAGAAAAAGGGACACAGAACATCCCAGGC CTAAGCTACATGGAGTATGAGAGAACAGCTGGAATGTGCTCTAGAAGCCCTGGG AGAAGGCCTCAGTTGAGGCTGAATTCAGATATGCCTGGGACCCGGTCTCTGTTA AGAGACCCCGGAGAGCTGGTGCAGAGCTGATGTGGGC
JAK2-OT1	GTGTGGTCAGGACAAACTGTTCCCTGAACTTAAAAGGTGAAGGACAAGACCCCA TATTATTATCCTGTATTAAAAAAGGAAATATACATATATGTACACAGACACCCCAT ATCACAGACAAGAAACTTCCCATAATTCAAAGGGAGACCATTTCCTTATTAGCAA AGGTGCGCATTACAGTATTTCATGACAGTTAAAAATTACACACCTACCACCTGTT TTGGTCAATCTTGCTAAAAAAGACACTGAAATAGACAATTTTCTTCTTTAAGGTAA AGACAATGTCTAATTTAGAAAATCTTCCTCTTGAGATTGCACAGTGAAAGGTATC AGTTAATTAAATATAAAAGCTTACCGTTTTTGTTTTTTTTTGAGACAGAGTTTTATT CAGTCGCCCAGGCTGGAG
JAK2-OT2	GGGGCTGGGAGAAGAGAGGAATGGGAGTTATTGATAATGGGTACAGTTTCAGTT TGAAAAGATGAAAAATTCTGAAGATGGATGGTGGTAGTGATTATACAACAATGTG AATATACTTAATGCTGCTGAACTATACAGTTTAAAATGGTTAAATGGTACATTTTA TGTATACGTTACCATAAAAAAAAAAGTCTGGACTATAATGTTAGAAGTAAAGATA GTAGTAATCTTATAGGAGAAAGAAGAGGATGGTGTTCAGAAAGAGCGTGGTGAG GGTGGGGAACTTCAAGGGTGCTGGCAATATTCTATTCCTGGGCATAGGTTATCA GTGG
JAK2-OT3	GTCCACTCTGTGTTACCCAGATCTTGTACAGGAAATGGAGTGTATTTAATATTGA TGACTCTGTATGTGTCATTTAATTGTCTCTATTGATTGGATTTCATCAGAGTGTGG GGCTTGGACAGAGCTTTGATTAGACTGTAAGGATTCTTTGCCGTTTTCTTTTTTC CGACACCATATCAATGTACCTTCTGGTGTGATATCACTTTCACAATCAATATCTG AAAAAAGCTCTGCACACAGGCCTGCAGGAAAAGAGAGAAGGCACTGCCTCTAA GTCCCCTTATACACATAGATTTAACCTCTCCAATTGAAAAAGGTTTATTGCATGTT TTAGAGCAGGATGGAGTGGGAAAAAAGACCTGAAGGAGTCTCCATAG
JAK2-OT4	GCCGGTTTAGGTATGGGAACCACACAACCTCTAAAGTGAAGGAGGCTTCTCCAG CAAAACCGAACGGCATATTCAGATGCTGGGTGAGGATTCATAATATTTAGCTATT

	TTGTTAATTAATATAACAGTAATTTCTTTATTCAGTCACAGAAACATCAATGTATCT ATGCTGAAGCACCATAATTTTATAAGCACAGTTATTGGGGGAAAATGTTACCTTT TTGTCCTAAAAAGACTCTCCACGCATGTAGCTAGGGAAAGCTAAGGTCAGTGGC AGAGTTATATGCACACCTCCACCACCAACATCACCACCACCACCTCCTCCTTCAT CTCTCATCCAGCTCTGCCTTAGGCCCTTTTTTTTCTTTTCTTTTCTTTTCTTTTTTT TTTCCCCAAGACAGGGTCTCACTC
JAK2-OT5	GGAACAGTGATCAGTTCTGTTACCTGACCATAATGGCTAAACAAAACAACCAAAT TATAAATTAAAAGGGGTTTGAATATATAGAACATTCATTTTTCAGCTACTAGCCAA AAAATACTATCTCTACCTGCTATCCCATGTGTTGTGTTCTTTTTAGACTGTCTAAT TTTGAGCTGAACTGTTTCTTAGACTGACAGACAAGTCATAAATCAAGTGATTGCT ATAAATGCTTTTTATTTATATAGAGAGTGTCTCAGATTTTTACCTTTCTTTCATAAA CTTGTCATAAATTTCTAATTCACCAATAGATGGTTGGTGTCCATTTTTCTTGAGTC TAAGACACTTTTAAAAATTTACTTGACTTGGTCTTTTACGTACTTTCTTCAGAGCC
JAK2-OT6	TCCGGATTCTCCTGCTGAGGCAGGAGAATCGCTTGAAGCTGGGAGGCAGAGAT TGCAGTGAGCTAAGATCAGGCCATTGCACTCCAGCCTGGGTGACAGAGCGAGA CTCTGTCTCAAAAAAACAAAACAAAACAAAAAAAAAGAGAAAGTCAGTAGCATGT AGATCAGGAGTGTCCAATCTTTTGGCTCCTCTGGAAGAAGAATTGTCTTGGGCC ACACATAAAATACACCATCACCTAACGATAGCTGATGAGCTTAAAAAAAATCACA GAAAAATATCATAATGTTTTAAGAATGTTTATGAATTTTTTACAAATTTCTGTTGGG CCACATTCAAAGCTGTCCTGGG
JAK2-OT7	CCGACTGCTCTGCCTTCTGAATCATATGTAACCAAATCAAGTCAAACAGGTTAGA AGACAACTCACACTTCAGTGTCATCTGTACTCTTATCTTCATGAGTGTGGGAATG TACAATCCACTTTCGCTCACTATATTAATTCATTCTGGTTCCTCATGTACTTAACC TATTTTTATTTTTTCAGTTTGGATACAACCCAAATCCTCTCAAGCCTTTTAAATGC AAAAAAAAAAAATAAATTTAAAGTATATGTAGTTAAAAATACTCATGTCTTTACCCA TTCCTTTGAGAATTTCTGTAGAGGCTTTCTCAAAATGCAGAGAGTGGAGGCAGT CATAACATATGATGCCTGCAGATTGGGGTATCTGTCATTTAATCAAGAGAAGAAA TAAACATTTTATGTCATTGATTTATCCATTTTAGTTGCTATCATCTTTATAGGTTAT GCCCCAATACATGGGC
JAK2-OT8	GTCCATGAATGACAGAGCACATTCACACTCATTCATTTACATATTTTCTATGCCTG TTTTTTATGCTAAATCTGTAGAGTTTAGTAATGGCAAAAGATACCGTATGGTCCA CAAGGCCTAAAATAGCTACTATCTGTACCTTACAGAAAAAAAAAAAGAGCTGACT CTTTTTCTGACCTCTGGTCTAAGTCAAGTCTGTCCCACAAGACACAAGGTACAAT GCTTTTTCCACATGAGGTCAGGAAGAATCTCAAAACTACAGAGTCAGGCCTCAC ATTATCAATAGGACCATCCTAGCCTTCTGCAAGCTTTCAGGCTTTAGTCAAAGCT GGCACATTTTCAGTGAATCCTTAGTCCACTGGTGGTCTGCAAGAGCCAGAGC
JAK2-OT9	GTAAGGGTGAAAATTCATTTGATGGAAATACTTGTGTATATTTAAAGACCCAATT GCTCCTCTGGAGCTTGTACTTTCAAGAATGATTAATCTGTGTAATAAACTGGTTA CTACAGTCATTACATATAATTTTGTGTGAATAGGCTTTTTCATTTTTAAGAAGTTT GTCTAGCTGAGATTAGTGGTGGATTTTCTCCCACTTCTGAAATGTTCATTTATACT GGTTGCATTTTAAGATCATGAAACAATTCCAGTTACATTGTAAAAAGGATATCTTA CGAGTAATTTTATTGAACAAGTTAGAGGCATAAGCTTAAGAGCATTTCCATGAAA CAACACATGCAGCATTCCAGGAACTTG

Supplementary Table 9 | List of bacterial plasmids used in this work

			ORF1		ORF2	
Name	Class (res)	Origin	Promoter	[RBS] Genes	Promoter	[RBS] Genes
pJC175e-DddI	AP ($carb^R$)	SC101	P_{psp}	[SD8] gIII	P_{ProD}	[SD8] DddI–VSV-G
MP6	MP ($chlor^R$)	cloDF13	P_{BAD}	dnaQ926, dam, seqA, emrR, ugi, cda1	P_c	araC
AP1	AP ($carb^R$)	SC101	P_{T7}	[SD8] gIII		
AP2	AP ($carb^R$)	SC101	P_{T7}	[sd8] gIII		
CP1-TCC	CP ($spec^R$)	ColE1	P_{Pro1}	[sd2] T7 RNAP–TCC linker–degron		
CP2-TCC	CP ($spec^R$)	ColE1	P_{Pro1}	[sd4U] T7 RNAP–TCC linker–degron		
CP2-ACC	CP ($spec^R$)	ColE1	P_{Pro1}	[sd4U] T7 RNAP–ACC linker–degron		
CP2-CCC	CP ($spec^R$)	ColE1	P_{Pro1}	[sd4U] T7 RNAP–CCC linker–degron		
CP2-GCC	CP ($spec^R$)	ColE1	P_{Pro1}	[sd4U] T7 RNAP–GCC linker–degron		
CP3-GCA	CP ($spec^R$)	ColE1	P_{Pro1}	[sd5] T7 RNAP–GCA linker–degron		
CP3-GCG	CP ($spec^R$)	ColE1	P_{Pro1}	[sd5] T7 RNAP–GCG linker–degron		
pBM10a	NCN target ($carb^R$)	SC101	N.A.	ACA target		
pBM10b	NCN target ($carb^R$)	SC101	N.A.	ACT target		
pBM10c	NCN target ($carb^R$)	SC101	N.A.	ACC target		
pBM10d	NCN target ($carb^R$)	SC101	N.A.	ACG target		
pBM10e	NCN target ($carb^R$)	SC101	N.A.	TCA target		
pBM10f	NCN target ($carb^R$)	SC101	N.A.	TCT target		
pBM10g	NCN target ($carb^R$)	SC101	N.A.	TCC target		
pBM10h	NCN target ($carb^R$)	SC101	N.A.	TCG target		
pBM10i	NCN target ($carb^R$)	SC101	N.A.	CCA target		
pBM10j	NCN target ($carb^R$)	SC101	N.A.	CCT target		
pBM10k	NCN target ($carb^R$)	SC101	N.A.	CCC target		
pBM10l	NCN target ($carb^R$)	SC101	N.A.	CCG target		
pBM10m	NCN target ($carb^R$)	SC101	N.A.	GCA target		
pBM10n	NCN target ($carb^R$)	SC101	N.A.	GCT target		
pBM10o	NCN target ($carb^R$)	SC101	N.A.	GCC target		
pBM10p	NCN target ($carb^R$)	SC101	N.A.	GCG target		
pBM22a	Window profiling ($carb^R$)	SC101	N.A.	$(TC)_{12}$ target, top strand		
pBM22b	Window profiling ($carb^R$)	SC101	N.A.	$(TC)_{13}$ target, top strand		
pBM22c	Window profiling ($carb^R$)	SC101	N.A.	$(TC)_{14}$ target, top strand		
pBM22d	Window profiling ($carb^R$)	SC101	N.A.	$(TC)_{15}$ target, top strand		
pBM22e	Window profiling ($carb^R$)	SC101	N.A.	$(TC)_{16}$ target, top strand		
pBM22f	Window profiling ($carb^R$)	SC101	N.A.	$(TC)_{17}$ target, top strand		
pBM22g	Window profiling ($carb^R$)	SC101	N.A.	$(TC)_{18}$ target, top strand		
pBM23a	Window profiling ($carb^R$)	SC101	N.A.	$(TC)_{12}$ target, bottom strand		
pBM23b	Window profiling ($carb^R$)	SC101	N.A.	$(TC)_{13}$ target, bottom strand		
pBM23c	Window profiling ($carb^R$)	SC101	N.A.	$(TC)_{14}$ target, bottom strand		
pBM23d	Window profiling ($carb^R$)	SC101	N.A.	$(TC)_{15}$ target, bottom strand		
pBM23e	Window profiling ($carb^R$)	SC101	N.A.	$(TC)_{16}$ target, botttom strand		
pBM23f	Window profiling ($carb^R$)	SC101	N.A.	$(TC)_{17}$ target, bottom strand		
pBM23g	Window profiling ($carb^R$)	SC101	N.A.	$(TC)_{18}$ target, bottom strand		

pBM13h	NCN assay ($spec^R$)	ColE1	P_{BAD}	[sd2] UGI–TALE3–DddA-G1397-N wildtype	P_c	araC
pBM13h-sd4U	window profiling ($spec^R$)	ColE1	P_{BAD}	[sd4U] UGI–TALE3–DddA-G1397-N wildtype		
pBM13	NCN assay ($spec^R$)	ColE1	P_{BAD}	[sd2] UGI–TALE3–DddA-G1397-N (Q1310R + S1330I + T1380I)	P_c	araC
pBM13-sd4U	window profiling ($spec^R$)	ColE1	P_{BAD}	[sd4U] UGI–TALE3–DddA-G1397-N (Q1310R + S1330I + T1380I)	P_c	araC
pBM13a	NCN assay ($spec^R$)	ColE1	P_{BAD}	[sd2] UGI–TALE3–DddA-G1397-N (T1314A + G1344R + V1364M + E1370K + T1380I)	P_c	araC
pBM13b	NCN assay ($spec^R$)	ColE1	P_{BAD}	[sd2] UGI–TALE3–DddA-G1397-N (N1342S + G1344R + V1364M + E1370K + T1380I)	P_c	araC
pBM13c	NCN assay ($spec^R$)	ColE1	P_{BAD}	[sd2] UGI–TALE3–DddA-G1397-N (A1341T + N1342S + E1370K + T1380I)	P_c	araC
pBM13e	NCN assay ($spec^R$)	ColE1	P_{BAD}	[sd2] UGI–TALE3–DddA-G1397-N (T1314A + G1344S + E1370K + T1380I)	P_c	araC
pBM13f	NCN assay ($spec^R$)	ColE1	P_{BAD}	[sd2] UGI–TALE3–DddA-G1397-N (T1314A + G1344S + E1370K + T1380I + E1396K)	P_c	araC
pBM13g	NCN assay ($spec^R$)	ColE1	P_{BAD}	[sd2] UGI–TALE3–DddA-G1397-N (S1330I + A1341V + N1342S + E1370K + T1380I)	P_c	araC
pBM13g-sd4U	window profiling ($spec^R$)	ColE1	P_{BAD}	[sd4U] UGI–TALE3–DddA-G1397-N (S1330I + A1341V + N1342S + E1370K + T1380I)	P_c	araC
pBM14d	NCN assay (kan^R)	p15A	P_{BAD}	[sd2] UGI–TALE4–DddA-G1397-C wildtype	P_c	araC
pBM14	NCN assay (kan^R)	p15A	P_{BAD}	[sd2] UGI–TALE4–DddA-G1397-C (T1413I)	P_c	araC
pBM14c	NCN assay (kan^R)	p15A	P_{BAD}	[sd2] UGI–TALE4–DddA-G1397-C (A1398T + T1413I)	P_c	araC
pBM19	window profiling ($spec^R$)	p15A	P_{BAD}	[sd4U] UGI–TALE4–DddA-G1397-C (T1413I)	P_c	araC
SPBM13	SP (none)	M13 f1	P_{gIII}	[sd8] UGI–TALE3–DddA-G1397-N wildtype	P_{BBa_J23101}	[sd8] UGI–TALE3–DddA-G1397-C wildtype
SPBM13a	SP (none)	M13 f1	P_{gIII}	[sd8] UGI–TALE3–DddA-G1397-N (T1380I)	P_{BBa_J23101}	[sd8] UGI–TALE3–DddA-G1397-C wildtype
SPBM14	SP (none)	M13 f1	P_{gIII}	[sd8] UGI–TALE3–DddA-G1397-N (E1347A)	P_{BBa_J23101}	[sd8] UGI–TALE3–DddA-G1397-C wildtype
SPBM29	SP (none)	M13 f1	P_{gIII}	[sd8] UGI–TALE3–DddA-G1397-N (S1330I + A1341V + N1342S + E1370K + T1380I)	P_{BBa_J23101}	[sd8] UGI–TALE3–DddA-G1397-C (T1413I)

Supplementary Table 10 | List of DNA sequences recognized by TALE proteins

DdCBE	Left-TALE target sequence	Right-TALE target sequence
T7	5'-T CCGACTTCGCGTT	5'-T CGTCGTTTGCT
ND1.1	5'-T CTAGCCTAGCCGTTT-3'	5'-T GAGTTTGATGCTCACCCT-3'
ND1.2	5'-T CCTAATGCTT-3'	5'-T ATATAGCCTAGA-3'
ND2	5'-T CTTAGCATACTCCTCAAT-3'	5'-T AGAACTGCTATTATT-3'
ND4	5'-T GCTAGTAACCACGTTCT-3'	5'-T CCTGTAAGTAGGAGAGT-3'
ND4.2	5'-T GAAGCTTCACC-3'	5'-T GGGCGATTATGA-3'
ND4.3	5'-T CTTCAATCAGCC-3'	5'-T GGCTGTTACT-3'
ND5.2 (mismatched)	5'-T CAAAACCATACCTCT-3'	5'-T GCTAGGCTGCCAATGT-3'
ND5.2 (no mismatch)	5'-T CAAAACCATACCTCT-3'	5'-T GCTAGGCTGCCAATGG-3'
ND5.4	5'-T CATAATAGTTACAA-3'	5'-T GCTAGGTGT-3'
ATP8 (mismatched)	5'-T ATTAAACACAAACTAT-3'	5'-T ATGGGCTTTGGT-3'
ATP8 (no mismatch)	5'-T ATTAAACACAAACTAC-3'	5'-T ATGGGCTTTGGT-3'
ND5.4	5'-T CATAATAGTTACAA-3'	5'-T GCTAGGTGT-3'
SIRT6	5'-T TACGCGGCGGGGCTGTC-3'	5'-T CCGGGAGGCCGCACTTG-3'
JAK2	5'-T CTGAAAAAGACTCTGCA-3'	5'-T CCATTTCTGTCATCGTA-3'

Supplementary Table 11 | Primers used for mammalian and bacteria cell genomic DNA amplification at sites targeted by DdCBEs

Site	HTS forward primer	HTS reverse primer
ND1.1	ACACTCTTTCCCTACACGACGCTCTTCCGATCTNNNNCTCACCATCGCTCTTCTACTATG	TGGAGTTCAGACGTGTGCTCTTCCGATCTGGCTAGGGTGACTTCATATGAG
ND1.2	ACACTCTTTCCCTACACGACGCTCTTCCGATCTNNNNGCCAACCTCCTACTCCTCAT	TGGAGTTCAGACGTGTGCTCTTCCGATCTGGGCGGTGATGTAGAGGG
ND2	ACACTCTTTCCCTACACGACGCTCTTCCGATCTNNNNCGTAAGCCTTCTCCTCACT	TGGAGTTCAGACGTGTGCTCTTCCGATCTGTTGAGTAGTAGGAATGCGGTAG
ND4	ACACTCTTTCCCTACACGACGCTCTTCCGATCTNNNNGACTTCAAACTCTACTCCCACTAATAG	TGGAGTTCAGACGTGTGCTCTTCCGATCTGTTGTGGTAAATATGTAGAGGGAG
ND4.2<br>ND4.3	ACACTCTTTCCCTACACGACGCTCTTCCGATCTNNNNCTGCCTACGACAAACAGACC	TGGAGTTCAGACGTGTGCTCTTCCGATCTTGGGAGTAGAGTTTGAAGTCC
ND5.2	ACACTCTTTCCCTACACGACGCTCTTCCGATCTNNNNCGGGTCCATCATCCACAAC	TGGAGTTCAGACGTGTGCTCTTCCGATCTAGAGTAATAGATAGGGCTCAGGC
ND5.4	ACACTCTTTCCCTACACGACGCTCTTCCGATCTNNNNGCAGTCTGCGCCCTTAC	TGGAGTTCAGACGTGTGCTCTTCCGATCTCGAATATCTTGTTCATTGTTAAGGTTG
ATP8	ACACTCTTTCCCTACACGACGCTCTTCCGATCTNNNNCTTTACAGTGAAATGCCCCAAC	TGGAGTTCAGACGTGTGCTCTTCCGATCTGGGGGCAATGAATGAAGCG
SIRT6 (on-target)	ACACTCTTTCCCTACACGACGCTCTTCCGATCTNNNNGGAAGCGGCCTCAACAAG	TGGAGTTCAGACGTGTGCTCTTCCGATCTGCCCCCTGATATTCCCAC
JAK2 (on-target)	ACACTCTTTCCCTACACGACGCTCTTCCGATCTNNNNGCTAGGATTACAGGTGTGAGAC	TGGAGTTCAGACGTGTGCTCTTCCGATCTCACCTGAAGAACTGGATCTATTTG
T7-DdCBE (for targeting NCN library plasmid	ACACTCTTTCCCTACACGACGCTCTTCCGATCTNNNNCGTTTTAGACTGAGCACGTCAATAC	TGGAGTTCAGACGTGTGCTCTTCCGATCTCGGATGCCGGGAGC
SIRT6-OT1	ACACTCTTTCCCTACACGACGCTCTTCCGATCTNNNNTGGCCAAGCAGGGATC	TGGAGTTCAGACGTGTGCTCTTCCGATCTCTGTTGCCTCCTTGGGTAC
SIRT6-OT2	ACACTCTTTCCCTACACGACGCTCTTCCGATCTNNNNGGGGGAGTCGGACTTAGAAGG	TGGAGTTCAGACGTGTGCTCTTCCGATCTCTTGGCACACAGCAGGTGCTC
SIRT6-OT3	ACACTCTTTCCCTACACGACGCTCTTCCGATCTNNNNTGCTAGGCCCCGATTTGTGG	TGGAGTTCAGACGTGTGCTCTTCCGATCTCCCCACTTCCTTCCTCACCCT
SIRT6-OT4	ACACTCTTTCCCTACACGACGCTCTTCCGATCTNNNNGACCCTTCCATTAGATCTGAGGGC	TGGAGTTCAGACGTGTGCTCTTCCGATCTGCTCTGGCTGGAGCACAGGAA

SIRT6-OT5	ACACTCTTTCCCTACACGACGCTCTTCCGATCTNNNNGGATCCAAGGTGCCTTCACCC	TGGAGTTCAGACGTGTGCTCTTCCGATCTACCACAGAGCCTCACCCACCA
SIRT6-OT6	ACACTCTTTCCCTACACGACGCTCTTCCGATCTNNNNGCTGGATTCGATCTGAGGTCAGCT	TGGAGTTCAGACGTGTGCTCTTCCGATCTCAGGCCCTACTGGGACGAACA
SIRT6-OT7	ACACTCTTTCCCTACACGACGCTCTTCCGATCTNNNNCCCAGGACAAAGTTGCTTTGGGGC	TGGAGTTCAGACGTGTGCTCTTCCGATCTCTCTTGCACAAGGTTGCCACCTC
SIRT6-OT8	ACACTCTTTCCCTACACGACGCTCTTCCGATCTNNNNGCCTCGCCAAGAAACGCCCAT	TGGAGTTCAGACGTGTGCTCTTCCGATCTATTCCGGGTAGGCGAGGAGGT
SIRT6-OT9	ACACTCTTTCCCTACACGACGCTCTTCCGATCTNNNNCCAGCACACCCTGATAAGC	TGGAGTTCAGACGTGTGCTCTTCCGATCTAGGGCCCCAGTGAGTG
SIRT6-OT10	ACACTCTTTCCCTACACGACGCTCTTCCGATCTNNNNCCAGGGCAACAGGTTTAAG	TGGAGTTCAGACGTGTGCTCTTCCGATCTGCCCACATCAGCTCTGC
JAK2-OT1	ACACTCTTTCCCTACACGACGCTCTTCCGATCTNNNNGTGTGGTCAGGACAAACTGTTCCC	TGGAGTTCAGACGTGTGCTCTTCCGATCTCTCCAGCCTGGGCGACTGAAT
JAK2-OT2	ACACTCTTTCCCTACACGACGCTCTTCCGATCTNNNNGGGGCTGGGAGAAGAGAGGAA	TGGAGTTCAGACGTGTGCTCTTCCGATCTCCACTGATAACCTATGCCCAGGA
JAK2-OT3	ACACTCTTTCCCTACACGACGCTCTTCCGATCTNNNNGTCCACTCTGTGTTACCCAGATC	TGGAGTTCAGACGTGTGCTCTTCCGATCTCTATGGAGACTCCTTCAGGTCTTTTTTCCC
JAK2-OT4	ACACTCTTTCCCTACACGACGCTCTTCCGATCTNNNNGCCGGTTTAGGTATGGGAACCAC	TGGAGTTCAGACGTGTGCTCTTCCGATCTGAGTGAGACCCTGTCTTGGGGA
JAK2-OT5	ACACTCTTTCCCTACACGACGCTCTTCCGATCTNNNNGGAACAGTGATCAGTTCTGTTACCTGAC	TGGAGTTCAGACGTGTGCTCTTCCGATCTGGCTCTGAAGAAAGTACGTAAAAGACCAAG
JAK2-OT6	ACACTCTTTCCCTACACGACGCTCTTCCGATCTNNNNTCCGGATTCTCCTGCTGAGGC	TGGAGTTCAGACGTGTGCTCTTCCGATCTCCCAGGACAGCTTTGAATGTGGC
JAK2-OT7	ACACTCTTTCCCTACACGACGCTCTTCCGATCTNNNNCCGACTGCTCTGCCTTCTGA	TGGAGTTCAGACGTGTGCTCTTCCGATCTGCCCATGTATTGGGGCATAACC
JAK2-OT8	ACACTCTTTCCCTACACGACGCTCTTCCGATCTNNNNGTCCATGAATGACAGAGCAC	TGGAGTTCAGACGTGTGCTCTTCCGATCTGCTCTGGCTCTTGCAGAC
JAK2-OT9	ACACTCTTTCCCTACACGACGCTCTTCCGATCTNNNNGTAAGGGTGAAAATTCATTTGATGG	TGGAGTTCAGACGTGTGCTCTTCCGATCTCAAGTTCCTGGAATGCTGCATG

Supplementary Table 12 | Sequences of unique molecular identifiers associated with each target plasmid for NCN context profiling and editing window profiling . For sequence context profiling, each target plasmid contains a target cytosine flanked by two nucleotides of either A, T, C or G.

Plasmid	UMI	Sequence context	No. of TC repeats (top or bottom)
pBM10a	TTTGTAGTCTAGTCT	ACA	
pBM10b	CATTATGATCGTACG	ACT	
pBM10c	CTATTCAGGGATTGA	ACC	
pBM10d	CTGATACCGGAAGAC	ACG	
pBM10e	ATCTCAGTTGAAGTG	TCA	
pBM10f	GTGTATACGACAGAG	TCT	
pBM10g	ACCGTGCACCTACCA	TCC	
pBM10h	AACCTCCTTAGTCTA	TCG	
pBM10i	AGTTCAGACCAATTG	CCA	
pBM10j	GTAGTTTGTCCAGAA	CCT	
pBM10k	CTCAGATTTTATCAC	CCC	
pBM10L	CAGAGGACGCACGCT	CCG	
pBM10m	CTACCTTTATGATCC	GCA	
pBM10n	TCCTTGGTCCTCGAG	GCT	
pBM10o	AAGAGGAGACGTCAG	GCC	
pBM10p	TCCAGATATCTTTAA	GCG	
pBM22a	TTTGTAGTCTAGTCT		12, top
pBM22b	CATTATGATCGTACG		13, top
pBM22c	CTATTCAGGGATTGA		14, top
pBM22d	CTGATACCGGAAGAC		15, top
pBM22e	ATCTCAGTTGAAGTG		16, top
pBM22f	GTGTATACGACAGAG		17, top
pBM22g	ACCGTGCACCTACCA		18, top
pBM23a	AACCTCCTTAGTCTA		12, bottom
pBM23b	AGTTCAGACCAATTG		13, bottom
pBM23c	GTAGTTTGTCCAGAA		14, bottom
pBM23d	CTCAGATTTTATCAC		15, bottom
pBM23e	CAGAGGACGCACGCT		16, bottom
pBM23f	CTACCTTTATGATCC		17, bottom
pBM23g	TCCTTGGTCCTCGAG		18, bottom

Supplementary Sequences 1 | TALE sequences used in DdCBEs

All right-side halves of DdCBEs have the general architecture of (from N- to C-terminus): *COX8A* MTS–3xFLAG–mitoTALE–2aa linker–DddA$_{tox}$ half–4aa linker–1x-UGI– *ATP5B* 3'UTR

All left-side halves of DdCBEs have the general architecture of (from N- to C-terminus): *SOD2* MTS–3xHA–mitoTALE–2aa linker–DddA$_{tox}$ half–4aa linker–1x-UGI– *SOD2* 3'UTR

mitoTALE domains are annotated as: bold for N-terminal domain, underlined for RVD and bolded italics for C-terminal domain.

ND1-DdCBE Right mitoTALE repeat
DIADLRTLGYSQQQQEKIKPKVRSTVAQHHEALVGHGFTHAHIVALSQHPAALGTVA
VKYQDMIAALPEATHEAIVGVGKQWSGARALEALLTVAGELRGPPLQLDTGQLLKIA
KRGGVTAVEAVHAWRNALTGAPLNLTPEQVVAIASNNGGKQALETVQALLPVLCQAH
GLTPQQVVAIASNIGGKQALETVQRLLPVLCQAHGLTPQQVVAIASNNGGKQALETVQ
RLLPVLCQAHGLTPEQVVAIASNGGGKQALETVQALLPVLCQAHGLTPEQVVAIASNG
GGKQALETVQALLPVLCQAHGLTPEQVVAIASNGGGKQALETVQALLPVLCQAHGLTP
EQVVAIASNNGGKQALETVQRLLPVLCQAHGLTPEQVVAIASNIGGKQALETVQALLPV
LCQAHGLTPEQVVAIASNGGGKQALETVQRLLPVLCQAHGLTPEQVVAIASNNGGKQA
LETVQALLPVLCQAHGLTPEQVVAIASHDGGKQALETVQALLPVLCQAHGLTPEQVVAI
ASNGGGKQALETVQALLPVLCQAHGLTPEQVVAIASHDGGKQALETVQRLLPVLCQA
HGLTPQQVVAIASNIGGKQALETVQRLLPVLCQAHGLTPEQVVAIASHDGGKQALETV
QALLPVLCQAHGLTPQQVVAIASHDGGKQALETVQRLLPVLCQAHGLTPQQVVAIASH
DGGKQALETVQRLLPVLCQAHGLTPQQVVAIASNGGGRPALE***SIVAQLSRPDPALAAL***
TNDHLVALACLGGRPALDAVKKGLG

ND1-DdCBE Left mitoTALE repeat
DIADLRTLGYSQQQQEKIKPKVRSTVAQHHEALVGHGFTHAHIVALSQHPAALGTVA
VKYQDMIAALPEATHEAIVGVGKQWSGARALEALLTVAGELRGPPLQLDTGQLLKIA
KRGGVTAVEAVHAWRNALTGAPLNLTPEQVVAIASHDGGKQALETVQALLPVLCQAH
GLTPQQVVAIASNGGGKQALETVQRLLPVLCQAHGLTPQQVVAIASNIGGKQALETVQ
RLLPVLCQAHGLTPEQVVAIASNNGGKQALETVQALLPVLCQAHGLTPEQVVAIASHD
GGKQALETVQALLPVLCQAHGLTPEQVVAIASHDGGKQALETVQALLPVLCQAHGLTP
EQVVAIASNGGGKQALETVQRLLPVLCQAHGLTPEQVVAIASNIGGKQALETVQALLPV
LCQAHGLTPEQVVAIASNNGGKQALETVQRLLPVLCQAHGLTPEQVVAIASHDGGKQA
LETVQALLPVLCQAHGLTPEQVVAIASHDGGKQALETVQALLPVLCQAHGLTPEQVVAI
ASNNGGKQALETVQALLPVLCQAHGLTPEQVVAIASNGGGKQALETVQRLLPVLCQA
HGLTPQQVVAIASNGGGKQALETVQRLLPVLCQAHGLTPQQVVAIASNGGGRPALE***SI***
VAQLSRPDPALAALTNDHLVALACLGGRPALDAVKKGLG

ND1.2-DdCBE Left mitoTALE repeat
DIADLRTLGYSQQQQEKIKPKVRSTVAQHHEALVGHGFTHAHIVALSQHPAALGTVA
VKYQDMIAALPEATHEAIVGVGKQWSGARALEALLTVAGELRGPPLQLDTGQLLKIA

KRGGVTAVEAVHAWRNALTGAPLNLTPEQVVAIASNIGGKQALETVQALLPVLCQAH
GLTPQQVVAIASNGGGKQALETVQRLLPVLCQAHGLTPQQVVAIASNIGGKQALETVQ
RLLPVLCQAHGLTPEQVVAIASNGGGKQALETVQALLPVLCQAHGLTPEQVVAIASNIG
GKQALETVQALLPVLCQAHGLTPEQVVAIASNNGGKQALETVQALLPVLCQAHGLTPE
QVVAIASHDGGKQALETVQRLLPVLCQAHGLTPQQVVAIASHDGGKQALETVQALLPV
LCQAHGLTPEQVVAIASNGGGKQALETVQRLLPVLCQAHGLTPEQVVAIASNIGGKQA
LETVQRLLPVLCQAHGLTPEQVVAIASNNGGKQALETVQALLPVLCQAHGLTPQQVVAI
ASNIGGRPALE***SIVAQLSRPDPALAALTNDHLVALACLGGRPALDAVKKGLG***

ND1.2-DdCBE Left mitoTALE repeat
**DIADLRTLGYSQQQQEKIKPKVRSTVAQHHEALVGHGFTHAHIVALSQHPAALGTVA
VKYQDMIAALPEATHEAIVGVGKQWSGARALEALLTVAGELRGPPLQLDTGQLLKIA
KRGGVTAVEAVHAWRNALTGAPLN**LTPEQVVAIASHDGGKQALETVQALLPVLCQAH
GLTPQQVVAIASHDGGKQALETVQRLLPVLCQAHGLTPQQVVAIASNGGGKQALETV
QRLLPVLCQAHGLTPEQVVAIASNIGGKQALETVQALLPVLCQAHGLTPEQVVAIASNI
GGKQALETVQALLPVLCQAHGLTPEQVVAIASNGGGKQALETVQALLPVLCQAHGLTP
EQVVAIASNNGGKQALETVQRLLPVLCQAHGLTPQQVVAIASHDGGKQALETVQALLP
VLCQAHGLTPEQVVAIASNGGGKQALETVQRLLPVLCQAHGLTPQQVVAIASNGGGR
PALE***SIVAQLSRPDPALAALTNDHLVALACLGGRPALDAVKKGLG***

ND2-DdCBE Right mitoTALE repeat
**DIADLRTLGYSQQQQEKIKPKVRSTVAQHHEALVGHGFTHAHIVALSQHPAALGTVA
VKYQDMIAALPEATHEAIVGVGKQWSGARALEALLTVAGELRGPPLQLDTGQLLKIA
KRGGVTAVEAVHAWRNALTGAPLN**LTPEQVVAIASNIGGKQALETVQALLPVLCQAH
GLTPQQVVAIASNNGGKQALETVQRLLPVLCQAHGLTPQQVVAIASNIGGKQALETVQ
RLLPVLCQAHGLTPEQVVAIASNIGGKQALETVQALLPVLCQAHGLTPEQVVAIASHDG
GKQALETVQALLPVLCQAHGLTPEQVVAIASNGGGKQALETVQALLPVLCQAHGLTPE
QVVAIASNNGGKQALETVQRLLPVLCQAHGLTPEQVVAIASHDGGKQALETVQALLPV
LCQAHGLTPEQVVAIASNGGGKQALETVQRLLPVLCQAHGLTPEQVVAIASNIGGKQA
LETVQALLPVLCQAHGLTPEQVVAIASNGGGKQALETVQALLPVLCQAHGLTPEQVVAI
ASNGGGKQALETVQALLPVLCQAHGLTPEQVVAIASNIGGKQALETVQRLLPVLCQAH
GLTPQQVVAIASNGGGKQALETVQRLLPVLCQAHGLTPQQVVAIASNGGGRPALE***SIV
AQLSRPDPALAALTNDHLVALACLGGRPALDAVKKGLG***

ND2-DdCBE Left mitoTALE repeat
**DIADLRTLGYSQQQQEKIKPKVRSTVAQHHEALVGHGFTHAHIVALSQHPAALGTVA
VKYQDMIAALPEATHEAIVGVGKQWSGARALEALLTVAGELRGPPLQLDTGQLLKIA
KRGGVTAVEAVHAWRNALTGAPLN**LTPEQVVAIASHDGGKQALETVQALLPVLCQAH
GLTPQQVVAIASNGGGKQALETVQRLLPVLCQAHGLTPQQVVAIASNGGGKQALETV
QRLLPVLCQAHGLTPEQVVAIASNIGGKQALETVQALLPVLCQAHGLTPEQVVAIASNN
GGKQALETVQALLPVLCQAHGLTPEQVVAIASHDGGKQALETVQALLPVLCQAHGLTP
EQVVAIASNIGGKQALETVQRLLPVLCQAHGLTPEQVVAIASNGGGKQALETVQALLPV
LCQAHGLTPEQVVAIASNIGGKQALETVQRLLPVLCQAHGLTPEQVVAIASHDGGKQA
LETVQALLPVLCQAHGLTPEQVVAIASNGGGKQALETVQALLPVLCQAHGLTPEQVVAI
ASHDGGKQALETVQALLPVLCQAHGLTPEQVVAIASHDGGKQALETVQRLLPVLCQAH
GLTPQQVVAIASNGGGKQALETVQRLLPVLCQAHGLTPEQVVAIASHDGGKQALETVQ

ALLPVLCQAHGLTPEQVVAIASNIGGKQALETVQALLPVLCQAHGLTPEQVVAIASNIGG
KQALETVQALLPVLCQAHGLTPQQVVAIASNGGGRPALE***SIVAQLSRPDPALAALTND***
HLVALACLGGRPALDAVKKGLG

ND4-DdCBE Right mitoTALE repeat
DIADLRTLGYSQQQQEKIKPKVRSTVAQHHEALVGHGFTHAHIVALSQHPAALGTVA
VKYQDMIAALPEATHEAIVGVGKQWSGARALEALLTVAGELRGPPLQLDTGQLLKIA
KRGGVTAVEAVHAWRNALTGAPLNLTPEQVVAIASHDGGKQALETVQALLPVLCQAH
GLTPQQVVAIASHDGGKQALETVQRLLPVLCQAHGLTPQQVVAIASNGGGKQALETV
QRLLPVLCQAHGLTPEQVVAIASNNGGKQALETVQALLPVLCQAHGLTPEQVVAIASN
GGGKQALETVQALLPVLCQAHGLTPEQVVAIASNIGGKQALETVQALLPVLCQAHGLT
PEQVVAIASNIGGKQALETVQRLLPVLCQAHGLTPEQVVAIASNNGGKQALETVQALLP
VLCQAHGLTPEQVVAIASNGGGKQALETVQRLLPVLCQAHGLTPEQVVAIASNIGGKQ
ALETVQRLLPVLCQAHGLTPEQVVAIASNNGGKQALETVQALLPVLCQAHGLTPEQVV
AIASNNGGKQALETVQALLPVLCQAHGLTPEQVVAIASNIGGKQALETVQALLPVLCQA
HGLTPEQVVAIASNNGGKQALETVQRLLPVLCQAHGLTPQQVVAIASNIGGKQALETV
QRLLPVLCQAHGLTPQQVVAIASNNGGKQALETVQRLLPVLCQAHGLTPQQVVAIASN
GGGRPALE***SIVAQLSRPDPALAALTNDHLVALACLGGRPALDAVKKGLG***

ND4-DdCBE Left mitoTALE repeat
DIADLRTLGYSQQQQEKIKPKVRSTVAQHHEALVGHGFTHAHIVALSQHPAALGTVA
VKYQDMIAALPEATHEAIVGVGKQWSGARALEALLTVAGELRGPPLQLDTGQLLKIA
KRGGVTAVEAVHAWRNALTGAPLNLTPEQVVAIASNNGGKQALETVQALLPVLCQAH
GLTPQQVVAIASHDGGKQALETVQRLLPVLCQAHGLTPQQVVAIASNGGGKQALETV
QRLLPVLCQAHGLTPEQVVAIASNIGGKQALETVQALLPVLCQAHGLTPEQVVAIASNN
GGKQALETVQALLPVLCQAHGLTPEQVVAIASNGGGKQALETVQALLPVLCQAHGLTP
EQVVAIASNIGGKQALETVQRLLPVLCQAHGLTPEQVVAIASNIGGKQALETVQALLPVL
CQAHGLTPEQVVAIASHDGGKQALETVQRLLPVLCQAHGLTPEQVVAIASHDGGKQAL
ETVQALLPVLCQAHGLTPEQVVAIASNIGGKQALETVQALLPVLCQAHGLTPEQVVAIA
SHDGGKQALETVQALLPVLCQAHGLTPEQVVAIASNNGGKQALETVQRLLPVLCQAH
GLTPQQVVAIASNGGGKQALETVQRLLPVLCQAHGLTPEQVVAIASNGGGKQALETVQ
ALLPVLCQAHGLTPEQVVAIASHDGGKQALETVQALLPVLCQAHGLTPQQVVAIASNG
GGRPALE***SIVAQLSRPDPALAALTNDHLVALACLGGRPALDAVKKGLG***

ND4.2-DdCBE Right mitoTALE repeat
DIADLRTLGYSQQQQEKIKPKVRSTVAQHHEALVGHGFTHAHIVALSQHPAALGTVA
VKYQDMIAALPEATHEAIVGVGKQWSGARALEALLTVAGELRGPPLQLDTGQLLKIA
KRGGVTAVEAVHAWRNALTGAPLNLTPEQVVAIASNNGGKQALETVQALLPVLCQAH
GLTPQQVVAIASNNGGKQALETVQRLLPVLCQAHGLTPQQVVAIASNNGGKQALETVQ
RLLPVLCQAHGLTPEQVVAIASHDGGKQALETVQALLPVLCQAHGLTPEQVVAIASNN
GGKQALETVQALLPVLCQAHGLTPEQVVAIASNIGGKQALETVQALLPVLCQAHGLTPE
QVVAIASNGGGKQALETVQRLLPVLCQAHGLTPEQVVAIASNGGGKQALETVQALLPV
LCQAHGLTPQQVVAIASNIGGKQALETVQRLLPVLCQAHGLTPEQVVAIASNGGGKQA
LETVQRLLPVLCQAHGLTPQQVVAIASNNGGKQALETVQRLLPVLCQAHGLTPQQVVA
IASNIGGRPALE***SIVAQLSRPDPALAALTNDHLVALACLGGRPALDAVKKGLG***

ND4.2-DdCBE Left mitoTALE repeat
DIADLRTLGYSQQQQEKIKPKVRSTVAQHHEALVGHGFTHAHIVALSQHPAALGTVA
VKYQDMIAALPEATHEAIVGVGKQWSGARALEALLTVAGELRGPPLQLDTGQLLKIA
KRGGVTAVEAVHAWRNALTGAPLNLTPEQVVAIASNNGGKQALETVQALLPVLCQAH
GLTPQQVVAIASNIGGKQALETVQRLLPVLCQAHGLTPQQVVAIASNIGGKQALETVQR
LLPVLCQAHGLTPEQVVAIASNNGGKQALETVQALLPVLCQAHGLTPEQVVAIASHDG
GKQALETVQALLPVLCQAHGLTPEQVVAIASNGGGKQALETVQALLPVLCQAHGLTPE
QVVAIASNGGGKQALETVQRLLPVLCQAHGLTPEQVVAIASHDGGKQALETVQALLPV
LCQAHGLTPQQVVAIASNIGGKQALETVQRLLPVLCQAHGLTPQQVVAIASHDGGKQA
LETVQRLLPVLCQAHGLTPQQVVAIASHDGGRPALE***SIVAQLSRPDPALAALTNDHLV***
ALACLGGRPALDAVKKGLG

ND4.3-DdCBE Right mitoTALE repeat
DIADLRTLGYSQQQQEKIKPKVRSTVAQHHEALVGHGFTHAHIVALSQHPAALGTVA
VKYQDMIAALPEATHEAIVGVGKQWSGARALEALLTVAGELRGPPLQLDTGQLLKIA
KRGGVTAVEAVHAWRNALTGAPLNLTPEQVVAIASNNGGKQALETVQALLPVLCQAH
GLTPQQVVAIASNNGGKQALETVQRLLPVLCQAHGLTPQQVVAIASHDGGKQALETVQ
RLLPVLCQAHGLTPEQVVAIASNGGGKQALETVQALLPVLCQAHGLTPEQVVAIASNN
GGKQALETVQALLPVLCQAHGLTPEQVVAIASNGGGKQALETVQALLPVLCQAHGLTP
EQVVAIASNGGGKQALETVQRLLPVLCQAHGLTPEQVVAIASNIGGKQALETVQALLPV
LCQAHGLTPEQVVAIASHDGGKQALETVQRLLPVLCQAHGLTPQQVVAIASNGGGRP
ALE***SIVAQLSRPDPALAALTNDHLVALACLGGRPALDAVKKGLG***

ND4.3-DdCBE Left mitoTALE repeat
DIADLRTLGYSQQQQEKIKPKVRSTVAQHHEALVGHGFTHAHIVALSQHPAALGTVA
VKYQDMIAALPEATHEAIVGVGKQWSGARALEALLTVAGELRGPPLQLDTGQLLKIA
KRGGVTAVEAVHAWRNALTGAPLNLTPEQVVAIASHDGGKQALETVQALLPVLCQAH
GLTPQQVVAIASNGGGKQALETVQRLLPVLCQAHGLTPQQVVAIASNGGGKQALETV
QRLLPVLCQAHGLTPEQVVAIASHDGGKQALETVQALLPVLCQAHGLTPEQVVAIASNI
GGKQALETVQALLPVLCQAHGLTPEQVVAIASNIGGKQALETVQALLPVLCQAHGLTPE
QVVAIASNGGGKQALETVQRLLPVLCQAHGLTPEQVVAIASHDGGKQALETVQALLPV
LCQAHGLTPQQVVAIASNIGGKQALETVQRLLPVLCQAHGLTPQQVVAIASNNGGKQA
LETVQRLLPVLCQAHGLTPEQVVAIASHDGGKQALETVQRLLPVLCQAHGLTPQQVVA
IASHDGGRPALE***SIVAQLSRPDPALAALTNDHLVALACLGGRPALDAVKKGLG***

ND5.2-DdCBE Right mitoTALE repeat (Note: Terminal NG RVD recognizes a
mismatched T instead of a G in the reference genome)
DIADLRTLGYSQQQQEKIKPKVRSTVAQHHEALVGHGFTHAHIVALSQHPAALGTVA
VKYQDMIAALPEATHEAIVGVGKQWSGARALEALLTVAGELRGPPLQLDTGQLLKIA
KRGGVTAVEAVHAWRNALTGAPLNLTPQQVVAIASNNGGKQALETVQRLLPVLCQA
HGLTPEQVVAIASHDGGKQALETVQRLLPVLCQAHGLTPQQVVAIASNGGGKQALETV
QRLLPVLCQAHGLTPEQVVAIASNIGGKQALETVQALLPVLCQAHGLTPQQVVAIASNN
GGKQALETVQRLLPVLCQAHGLTPQQVVAIASNNGGKQALETVQRLLPVLCQAHGLTP
EQVVAIASHDGGKQALETVQRLLPVLCQAHGLTPQQVVAIASNGGGKQALETVQRLLP
VLCQAHGLTPQQVVAIASNNGGKQALETVQRLLPVLCQAHGLTPEQVVAIASHDGGK
QALETVQRLLPVLCQAHGLTPEQVVAIASHDGGKQALETVQRLLPVLCQAHGLTPEQV

VAIASNIGGKQALETVQALLPVLCQAHGLTPEQVVAIASNIGGKQALETVQALLPVLCQA
HGLTPQQVVAIASNGGGKQALETVQRLLPVLCQAHGLTPQQVVAIASNNGGKQALETV
QRLLPVLCQAHGLTPQQVVAIASNGGGRPALE***SIVAQLSRPDPALAALTNDHLVALAC
LGGRPALDAVKKGLG***

ND5.2-DdCBE Right mitoTALE repeat (non-mismatched)
**DIADLRTLGYSQQQQEKIKPKVRSTVAQHHEALVGHGFTHAHIVALSQHPAALGTVA
VKYQDMIAALPEATHEAIVGVGKQWSGARALEALLTVAGELRGPPLQLDTGQLLKIA
KRGGVTAVEAVHAWRNALTGAPLN**LTPQQVVAIASNNGGKQALETVQRLLPVLCQA
HGLTPEQVVAIASHDGGKQALETVQRLLPVLCQAHGLTPQQVVAIASNGGGKQALETV
QRLLPVLCQAHGLTPEQVVAIASNIGGKQALETVQALLPVLCQAHGLTPQQVVAIASNN
GGKQALETVQRLLPVLCQAHGLTPQQVVAIASNNGGKQALETVQRLLPVLCQAHGLTP
EQVVAIASHDGGKQALETVQRLLPVLCQAHGLTPQQVVAIASNGGGKQALETVQRLLP
VLCQAHGLTPQQVVAIASNNGGKQALETVQRLLPVLCQAHGLTPEQVVAIASHDGGK
QALETVQRLLPVLCQAHGLTPEQVVAIASHDGGKQALETVQRLLPVLCQAHGLTPEQV
VAIASNIGGKQALETVQALLPVLCQAHGLTPEQVVAIASNIGGKQALETVQALLPVLCQA
HGLTPQQVVAIASNGGGKQALETVQRLLPVLCQAHGLTPQQVVAIASNNGGKQALETV
QRLLPVLCQAHGLTPQQVVAIASNNGGRPALE***SIVAQLSRPDPALAALTNDHLVALAC
LGGRPALDAVKKGLG***

ND5.2-DdCBE Left mitoTALE repeat
**DIADLRTLGYSQQQQEKIKPKVRSTVAQHHEALVGHGFTHAHIVALSQHPAALGTVA
VKYQDMIAALPEATHEAIVGVGKQWSGARALEALLTVAGELRGPPLQLDTGQLLKIA
KRGGVTAVEAVHAWRNALTGAPLN**LTPEQVVAIASHDGGKQALETVQALLPVLCQAH
GLTPQQVVAIASNIGGKQALETVQRLLPVLCQAHGLTPQQVVAIASNIGGKQALETVQR
LLPVLCQAHGLTPEQVVAIASNIGGKQALETVQALLPVLCQAHGLTPEQVVAIASNIGGK
QALETVQALLPVLCQAHGLTPEQVVAIASHDGGKQALETVQALLPVLCQAHGLTPEQV
VAIASHDGGKQALETVQRLLPVLCQAHGLTPEQVVAIASNIGGKQALETVQALLPVLCQ
AHGLTPEQVVAIASNGGGKQALETVQRLLPVLCQAHGLTPEQVVAIASNIGGKQALETV
QALLPVLCQAHGLTPEQVVAIASHDGGKQALETVQALLPVLCQAHGLTPEQVVAIASH
DGGKQALETVQALLPVLCQAHGLTPEQVVAIASNGGGKQALETVQRLLPVLCQAHGLT
PQQVVAIASHDGGKQALETVQRLLPVLCQAHGLTPQQVVAIASNGGGRPALE***SIVAQL
SRPDPALAALTNDHLVALACLGGRPALDAVKKGLG***

ND5.4-DdCBE Right mitoTALE repeat
**DIADLRTLGYSQQQQEKIKPKVRSTVAQHHEALVGHGFTHAHIVALSQHPAALGTVA
VKYQDMIAALPEATHEAIVGVGKQWSGARALEALLTVAGELRGPPLQLDTGQLLKIA
KRGGVTAVEAVHAWRNALTGAPLN**LTPEQVVAIASNNGGKQALETVQALLPVLCQAH
GLTPQQVVAIASHDGGKQALETVQRLLPVLCQAHGLTPQQVVAIASNGGGKQALETV
QRLLPVLCQAHGLTPEQVVAIASNIGGKQALETVQALLPVLCQAHGLTPEQVVAIASNN
GGKQALETVQALLPVLCQAHGLTPEQVVAIASNNGGKQALETVQALLPVLCQAHGLTP
EQVVAIASNGGGKQALETVQRLLPVLCQAHGLTPEQVVAIASNNGGKQALETVQALLP
VLCQAHGLTPQQVVAIASNGGGRPALE***SIVAQLSRPDPALAALTNDHLVALACLGGR
PALDAVKKGLG***

ND5.4-DdCBE Left mitoTALE repeat
DIADLRTLGYSQQQQEKIKPKVRSTVAQHHEALVGHGFTHAHIVALSQHPAALGTVA
VKYQDMIAALPEATHEAIVGVGKQWSGARALEALLTVAGELRGPPLQLDTGQLLKIA
KRGGVTAVEAVHAWRNALTGAPLNLTPEQVVAIASHDGGKQALETVQALLPVLCQAH
GLTPQQVVAIASNIGGKQALETVQRLLPVLCQAHGLTPQQVVAIASNGGGKQALETVQ
RLLPVLCQAHGLTPEQVVAIASNIGGKQALETVQALLPVLCQAHGLTPEQVVAIASNIG
GKQALETVQALLPVLCQAHGLTPEQVVAIASNGGGKQALETVQALLPVLCQAHGLTPE
QVVAIASNIGGKQALETVQRLLPVLCQAHGLTPEQVVAIASNNGGKQALETVQALLPVL
CQAHGLTPEQVVAIASNGGGKQALETVQRLLPVLCQAHGLTPEQVVAIASNGGGKQA
LETVQALLPVLCQAHGLTPEQVVAIASNIGGKQALETVQALLPVLCQAHGLTPEQVVAI
ASHDGGKQALETVQRLLPVLCQAHGLTPQQVVAIASNIGGKQALETVQRLLPVLCQAH
GLTPQQVVAIASNIGGRPALE***SIVAQLSRPDPALAALTNDHLVALACLGGRPALDAVK***
KGLG

ATP8-DdCBE Right mitoTALE repeat
DIADLRTLGYSQQQQEKIKPKVRSTVAQHHEALVGHGFTHAHIVALSQHPAALGTVA
VKYQDMIAALPEATHEAIVGVGKQWSGARALEALLTVAGELRGPPLQLDTGQLLKIA
KRGGVTAVEAVHAWRNALTGAPLNLTPQQVVAIASNIGGKQALETVQRLLPVLCQAH
GLTPEQVVAIASNGGGKQALETVQRLLPVLCQAHGLTPQQVVAIASNNGGKQALETVQ
RLLPVLCQAHGLTPEQVVAIASNNGGKQALETVQALLPVLCQAHGLTPQQVVAIASNN
GGKQALETVQRLLPVLCQAHGLTPQQVVAIASHDGGKQALETVQRLLPVLCQAHGLTP
EQVVAIASNGGGKQALETVQRLLPVLCQAHGLTPQQVVAIASNGGGKQALETVQRLLP
VLCQAHGLTPQQVVAIASNGGGKQALETVQRLLPVLCQAHGLTPEQVVAIASNNGGK
QALETVQRLLPVLCQAHGLTPEQVVAIASNNGGKQALETVQRLLPVLCQAHGLTPQQV
VAIASNGGGRPALE***SIVAQLSRPDPALAALTNDHLVALACLGGRPALDAVKKGLG***

ATP8-DdCBE Left mitoTALE repeat (Note: Terminal NG RVD recognizes a mismatched
T instead of a C in the reference genome)
DIADLRTLGYSQQQQEKIKPKVRSTVAQHHEALVGHGFTHAHIVALSQHPAALGTVA
VKYQDMIAALPEATHEAIVGVGKQWSGARALEALLTVAGELRGPPLQLDTGQLLKIA
KRGGVTAVEAVHAWRNALTGAPLNLTPEQVVAIASNIGGKQALETVQALLPVLCQAH
GLTPQQVVAIASNGGGKQALETVQRLLPVLCQAHGLTPQQVVAIASNGGGKQALETV
QRLLPVLCQAHGLTPEQVVAIASNIGGKQALETVQALLPVLCQAHGLTPEQVVAIASNI
GGKQALETVQALLPVLCQAHGLTPEQVVAIASNIGGKQALETVQALLPVLCQAHGLTPE
QVVAIASHDGGKQALETVQRLLPVLCQAHGLTPEQVVAIASNIGGKQALETVQALLPVL
CQAHGLTPEQVVAIASHDGGKQALETVQRLLPVLCQAHGLTPEQVVAIASNIGGKQAL
ETVQALLPVLCQAHGLTPEQVVAIASNIGGKQALETVQALLPVLCQAHGLTPEQVVAIA
SNIGGKQALETVQALLPVLCQAHGLTPEQVVAIASHDGGKQALETVQRLLPVLCQAHG
LTPQQVVAIASNGGGKQALETVQRLLPVLCQAHGLTPEQVVAIASNIGGKQALETVQAL
LPVLCQAHGLTPQQVVAIASNGGGRPALE***SIVAQLSRPDPALAALTNDHLVALACLG***
GRPALDAVKKGLG

ATP8-DdCBE Left mitoTALE repeat (non-mismatched)
DIADLRTLGYSQQQQEKIKPKVRSTVAQHHEALVGHGFTHAHIVALSQHPAALGTVA
VKYQDMIAALPEATHEAIVGVGKQWSGARALEALLTVAGELRGPPLQLDTGQLLKIA

KRGGVTAVEAVHAWRNALTGAPLNLTPEQVVAIASNIGGKQALETVQALLPVLCQAH
GLTPQQVVAIASNGGGKQALETVQRLLPVLCQAHGLTPQQVVAIASNGGGKQALETV
QRLLPVLCQAHGLTPEQVVAIASNIGGKQALETVQALLPVLCQAHGLTPEQVVAIASNI
GGKQALETVQALLPVLCQAHGLTPEQVVAIASNIGGKQALETVQALLPVLCQAHGLTPE
QVVAIASHDGGKQALETVQRLLPVLCQAHGLTPEQVVAIASNIGGKQALETVQALLPVL
CQAHGLTPEQVVAIASHDGGKQALETVQRLLPVLCQAHGLTPEQVVAIASNIGGKQAL
ETVQALLPVLCQAHGLTPEQVVAIASNIGGKQALETVQALLPVLCQAHGLTPEQVVAIA
SNIGGKQALETVQALLPVLCQAHGLTPEQVVAIASHDGGKQALETVQRLLPVLCQAHG
LTPQQVVAIASNGGGKQALETVQRLLPVLCQAHGLTPEQVVAIASNIGGKQALETVQAL
LPVLCQAHGLTPQQVVAIASHDGGRPALE***SIVAQLSRPDPALAALTNDHLVALACLGG***
RPALDAVKKGLG

Supplementary sequences 2 | Sequences of full-length DddA variants

DddA1 (T1380I)
GSYALGPYQISAPQLPAYNGQTVGTFYYVNDAGGLESKVFSSGGPTPYPNYANAGHV
EGQSALFMRDNGISEGLVFHNNPEGTCGFCVNMIETLLPENAKMTVVPPEGAIPVKRG
ATGETKVFTGNSNSPKSPTKGGC

DddA2 (T1314A + T1380I)
GSYALGPYQISAPQLPAYNGQTVGAFYYVNDAGGLESKVFSSGGPTPYPNYANAGHV
EGQSALFMRDNGISEGLVFHNNPEGTCGFCVNMIETLLPENAKMTVVPPEGAIPVKRG
ATGETKVFTGNSNSPKSPTKGGC

DddA3 (T1314A + T1380I + E1396K)
GSYALGPYQISAPQLPAYNGQTVGAFYYVNDAGGLESKVFSSGGPTPYPNYANAGHV
EGQSALFMRDNGISEGLVFHNNPEGTCGFCVNMIETLLPENAKMTVVPPKGAIPVKRG
ATGETKVFTGNSNSPKSPTKGGC

DddA4 (T1380I + T1413I)
GSYALGPYQISAPQLPAYNGQTVGTFYYVNDAGGLESKVFSSGGPTPYPNYANAGHV
EGQSALFMRDNGISEGLVFHNNPEGTCGFCVNMIETLLPENAKMTVVPPEGAIPVKRG
ATGETKVFIGNSNSPKSPTKGGC

DddA5 (Q1310R + S1330I + T1380I)
GSYALGPYQISAPQLPAYNGRTVGTFYYVNDAGGLESKVFISGGPTPYPNYANAGHVE
GQSALFMRDNGISEGLVFHNNPEGTCGFCVNMIETLLPENAKMTVVPPEGAIPVKRGA
TGETKVFTGNSNSPKSPTKGGC

DddA6 (Q1310R + S1330I + T1380I + T1413I)
GSYALGPYQISAPQLPAYNGRTVGTFYYVNDAGGLESKVFISGGPTPYPNYANAGHVE
GQSALFMRDNGISEGLVFHNNPEGTCGFCVNMIETLLPENAKMTVVPPEGAIPVKRGA
TGETKVFIGNSNSPKSPTKGGC

DddA7 (T1314A + G1344R + V1364M + E1370K + T1380I + T1413I)
GSYALGPYQISAPQLPAYNGQTVGAFYYVNDAGGLESKVFSSGGPTPYPNYANARHV
EGQSALFMRDNGISEGLMFHNNPKGTCGFCVNMIETLLPENAKMTVVPPEGAIPVKRG
ATGETKVFIGNSNSPKSPTKGGC

DddA8 (N1342S + G1344R + V1364M + E1370K + T1380I + T1413I)
GSYALGPYQISAPQLPAYNGQTVGTFYYVNDAGGLESKVFSSGGPTPYPNYASARHV
EGQSALFMRDNGISEGLMFHNNPKGTCGFCVNMIETLLPENAKMTVVPPEGAIPVKRG
ATGETKVFIGNSNSPKSPTKGGC

DddA9 (T1314A + G1344S + E1370K + T1380I + A1398T + T1413I)
GSYALGPYQISAPQLPAYNGQTVGAFYYVNDAGGLESKVFSSGGPTPYPNYANASHV
EGQSALFMRDNGISEGLVFHNNPKGTCGFCVNMIETLLPENAKMTVVPPEGTIPVKRG
ATGETKVFIGNSNSPKSPTKGGC

DddA10 (T1314A + G1344S + E1370K + T1380I + E1396K + A1398T + T1413I)
GSYALGPYQISAPQLPAYNGQTVGAFYYVNDAGGLESKVFSSGGPTPYPNYANASHV
EGQSALFMRDNGISEGLVFHNNPKGTCGFCVNMIETLLPENAKMTVVPPKGTIPVKRG
ATGETKVFIGNSNSPKSPTKGGC

DddA11 (S1330I + A1341V + N1342S + E1370K + T1380I + T1413I)
GSYALGPYQISAPQLPAYNGQTVGTFYYVNDAGGLESKVFISGGPTPYPNYVSAGHVE
GQSALFMRDNGISEGLVFHNNPKGTCGFCVNMIETLLPENAKMTVVPPEGAIPVKRGA
TGETKVFIGNSNSPKSPTKGGC

DddA-7.9.1(E1325K + S1330I + A1341V + N1342S + E1370K + N1378S + T1380I + T1413I)
GSYALGPYQISAPQLPAYNGQTVGTFYYVNDAGGLKSKVFISGGPTPYPNYVSAGHVE
GQSALFMRDNGISEGLVFHNNPKGTCGFCVSMIETLLPENAKMTVVPPEGAIPVKRGA
TGETKVFIGNSNSPKSPTKGGC

DddA-7.12.1(S1330I + A1341I + N1342S + E1370K + N1378S + T1380I + P1394S + T1413I)
GSYALGPYQISAPQLPAYNGQTVGTFYYVNDAGGLESKVFISGGPTPYPNYISAGHVE
GQSALFMRDNGISEGLVFHNNPKGTCGFCVSMIETLLPENAKMTVVSPEGAIPVKRGA
TGETKVFIGNSNSPKSPTKGGC

DddA-7.12.2 (S1330I + P1334S + A1341V + N1342S + E1370K + N1378S + T1380I + T1413I)
GSYALGPYQISAPQLPAYNGQTVGTFYYVNDAGGLESKVFISGGSTPYPNYVSAGHVE
GQSALFMRDNGISEGLVFHNNPKGTCGFCVSMIETLLPENAKMTVVPPEGAIPVKRGA
TGETKVFIGNSNSPKSPTKGGC

DddA-7.12.3 (S1330I + P1334S + P1336S + A1341V + N1342S + E1370K + N1378S + T1380I + T1413I)
GSYALGPYQISAPQLPAYNGQTVGTFYYVNDAGGLESKVFISGGSTSYPNYVSAGHVE
GQSALFMRDNGISEGLVFHNNPKGTCGFCVSMIETLLPENAKMTVVPPEGAIPVKRGA
TGETKVFIGNSNSPKSPTKGGC

Supplementary Sequences 3 | Sequences used in fluorescence-activated cell sorting of DdCBE expressing cells

Right-side halves of DdCBE have the general architecture of (from N- to C-terminus): *COX8A* MTS–3xFLAG–mitoTALE–GS linker–DddA half–SGGS linker–1x-UGI–GSG linker–P2A–eGFP–*ATP5B* 3'UTR

Right-side halves of DdCBE have the general architecture of (from N- to C-terminus): *SOD2* MTS–3xHA–mitoTALE–GS linker–DddA half–SGGS linker–1x-UGI–GSG linker–P2A–mCherry–*SOD2* 3'UTR

P2A sequence
ATNFSLLKQAGDVEENPGP

eGFP sequence
MVSKGEELFTGVVPILVELDGDVNGHKFSVSGEGEGDATYGKLTLKFICTTGKLPVPW
PTLVTTLTYGVQCFSRYPDHMKQHDFFKSAMPEGYVQERTIFFKDDGNYKTRAEVKF
EGDTLVNRIELKGIDFKEDGNILGHKLEYNYNSHNVYIMADKQKNGIKVNFKIRHNIEDG
SVQLADHYQQNTPIGDGPVLLPDNHYLSTQSALSKDPNEKRDHMVLLEFVTAAGITLG
MDELYK

mCherry sequence
MVSKGEEDNMAIIKEFMRFKVHMEGSVNGHEFEIEGEGEGRPYEGTQTAKLKVTKGG
PLPFAWDILSPQFMYGSKAYVKHPADIPDYLKLSFPEGFKWERVMNFEDGGVVTVTQ
DSSLQDGEFIYKVKLRGTNFPSDGPVMQKKTMGWEASSERMYPEDGALKGEIKQRLK
LKDGGHYDAEVKTTYKAKKPVQLPGAYNVNIKLDITSHNEDYTIVEQYERAEGRHSTG
GMDELYK

Supplementary Note 1. Mitochondrial editing efficiencies of DdCBEs containing a mismatched or non-mismatched terminal TALE repeat

The original right TALE in ND5.2-DdCBE contained an RVD in the terminal repeat that recognized a mismatched thymine instead of guanine.The original left TALE in ATP8-DdCBE contained an RVD in the terminal repeat that recognized a mismatched thymine instead of cytosine[48]. To clarify the effect of a mismatched RVD on mitochondrial editing efficiencies, we compared the base editing activities between the DdCBE containing the mismatched RVD (labelled as T) and the variant containing the non-mismatched RVD, which is labelled as G for ND5.2-DdCBE (**a**) and C for ATP8-DdCBE (**b**).

The editing efficiencies for mismatched DdCBEs containing DddA variants were generally comparable to or resulted in 2-10% higher average editing than the equivalent non-mismatched DdCBE (see **a** and **b**). Given that DdCBEs containing DddA6 or DddA11 resulted in similar editing efficiencies when tested as a mismatched TALE or non-mismatched TALE, all subsequent figures, except for Figs. 2c, 2d, 3d and 3e, are produced from DdCBEs containing the original mismatched RVD. Values and error bars reflect the mean±s.d of n=3 independent biological replicates

a

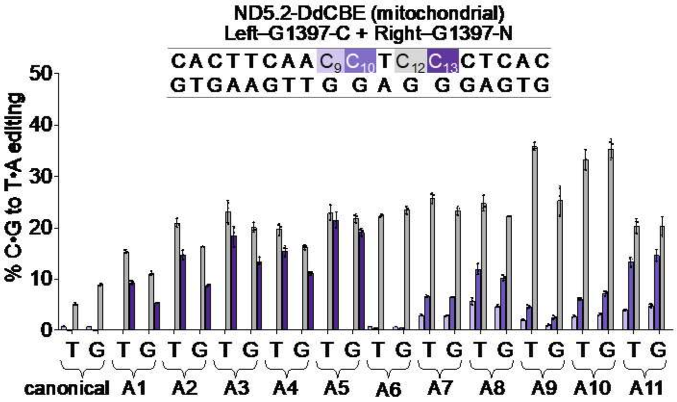

b

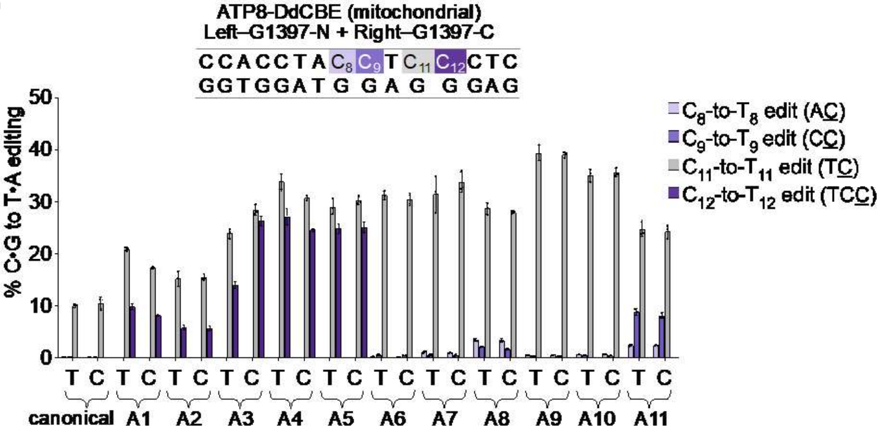

Supplementary Note 2. Representative FACS gating plots for eGFP⁺/mCherry⁺ cells

FACs gating plots for eGFP⁺ and mCherry⁺ cell sorting to isolate HeLa cells (Graphic 1), K562 cells (Graphic 2), U2OS cells (Graphic 3) and HEK293T cells (Graphic 4) expressing both halves of ND5.2-DdCBE. The image data was generated on a Sony LE-MA900 cytometer using Cell Sorter Software v. 3.0.5. Cells were initially gated on population using FSC-A/BSC-A (Gate A), then sorted for singlets using FSC-A/FSC-H (Singlet). Live cells were sorted for by gating mCherry-positive and eGFP-positive cells (Double positive). Single-color eGFP and mCherry controls were used for compensation.

Graphic 1

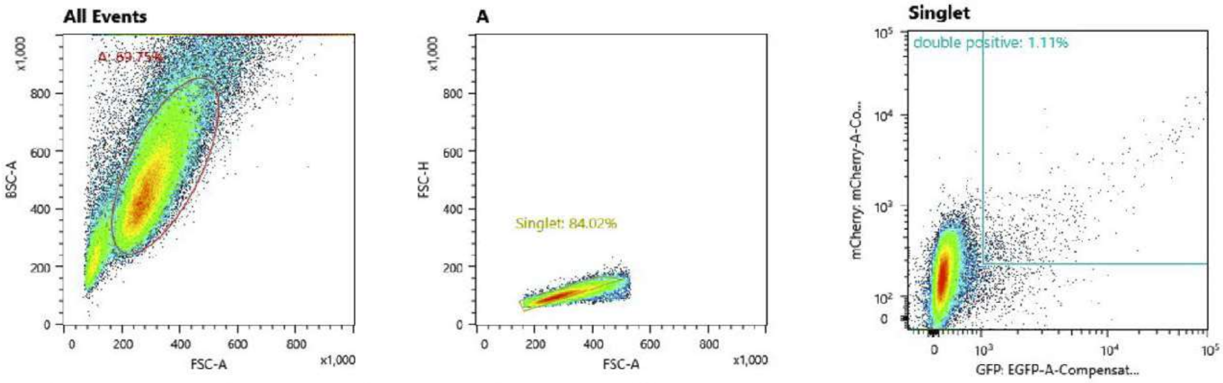

Name	Events	%Parent	%Total
All Events	100,000	0.00%	100.00%
A	69,752	69.75%	69.75%
Singlet	58,609	84.02%	58.61%
double po...	652	1.11%	0.65%

Graphic 2

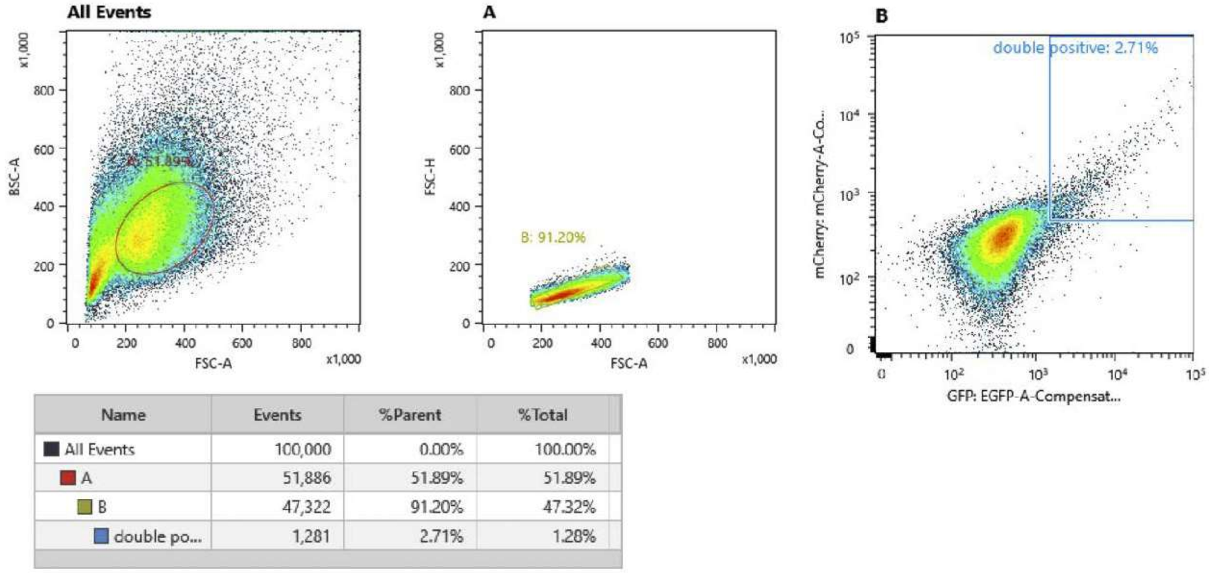

Name	Events	%Parent	%Total
All Events	100,000	0.00%	100.00%
A	51,886	51.89%	51.89%
B	47,322	91.20%	47.32%
double po...	1,281	2.71%	1.28%

Graphic 3

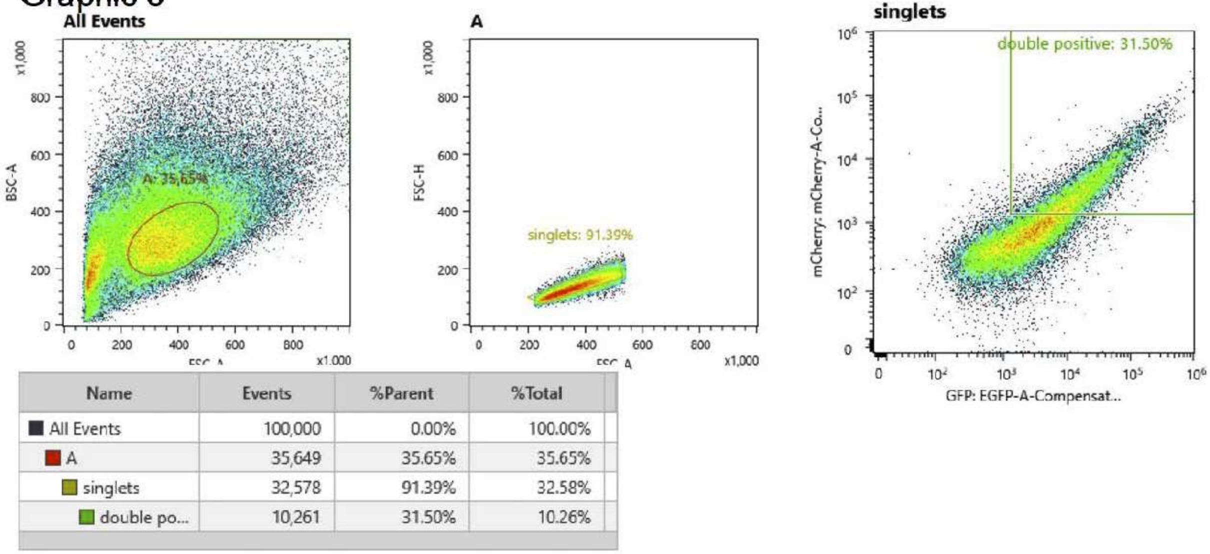

Name	Events	%Parent	%Total
All Events	100,000	0.00%	100.00%
A	35,649	35.65%	35.65%
singlets	32,578	91.39%	32.58%
double po...	10,261	31.50%	10.26%

Graphic 4

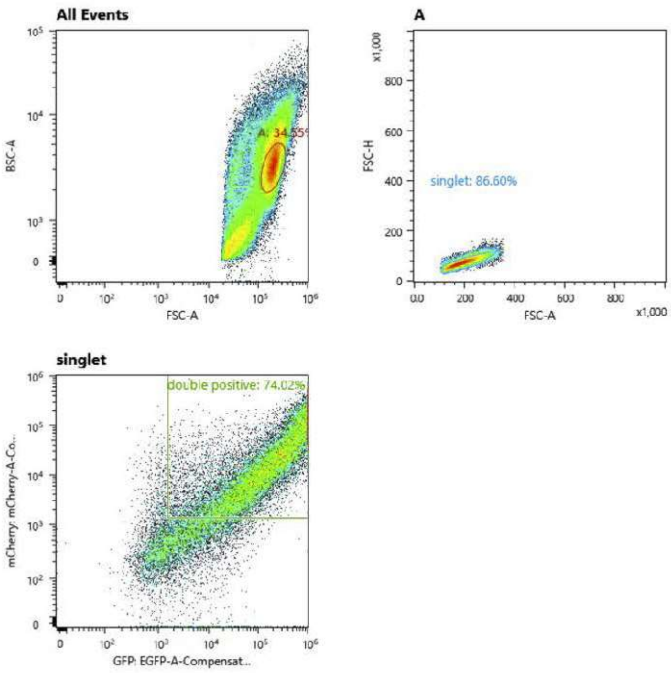

Gates and Statistics

Name	Events	%Parent	%Total
All Events	100,000	0.00%	100.00%
A	34,545	34.55%	34.55%
singlet	29,917	86.60%	29.92%
double po...	22,145	74.02%	22.15%

Supplementary Note 3. Nuclear editing efficiencies of DdCBEs containing N-terminal UGI fusions or C-terminal UGI fusions

We previously reported that the N-terminal 2xUGI fusion of a nuclear-targeting DdCBE resulted in more efficient nuclear base editing compared to a C-terminal 1xUGI fusion[18]. We compared the editing efficiencies of these two architectures in SIRT6-DdCBE (**a**) and JAK2-DdCBE (**b**). Consistent with earlier observations, N-terminal UGI fusions generally yielded higher TC and non-TC editing efficiencies compared to C-terminal fusions, except for canonical JAK2-DdCBE. Values and error bars reflect the mean±s.d of n=3 independent biological replicates

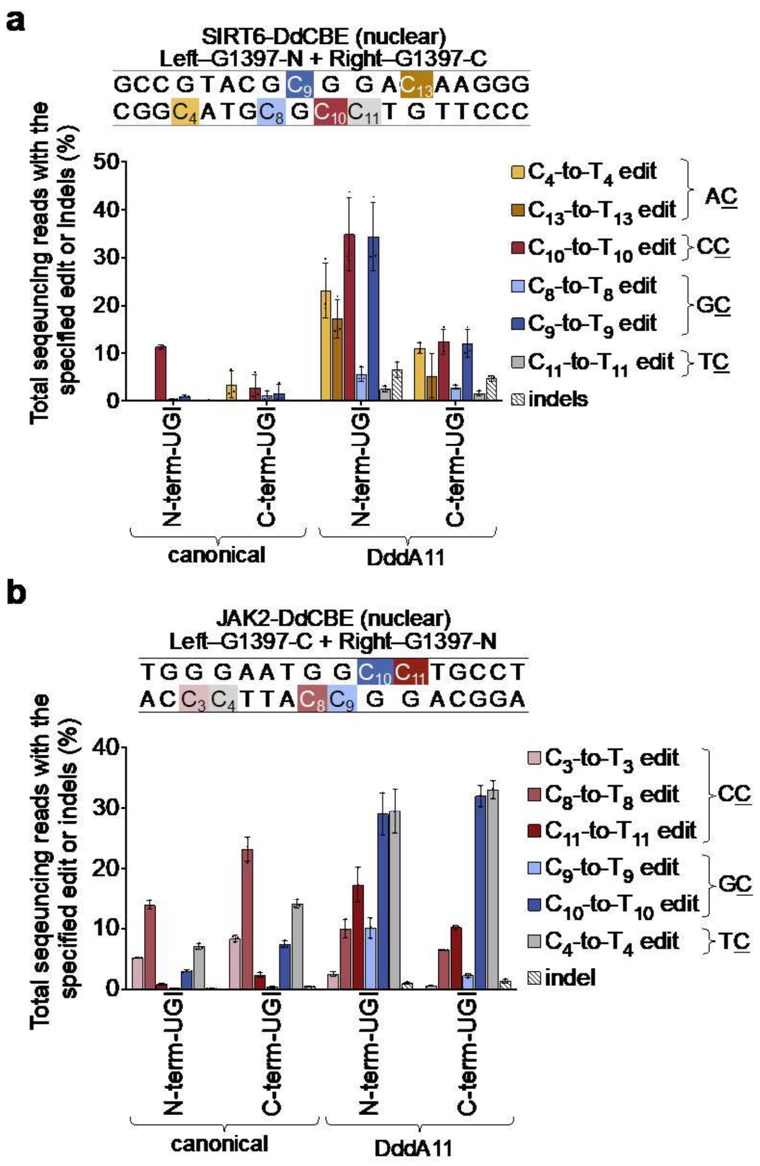

Supplementary References

45 Yu, Q. *et al.* Single-strand specificity of APOBEC3G accounts for minus-strand deamination of the HIV genome. *Nat Struct Mol Biol* **11**, 435-442, doi:10.1038/nsmb758 (2004).

46 Maiti, A. *et al.* Crystal structure of the catalytic domain of HIV-1 restriction factor APOBEC3G in complex with ssDNA. *Nature Communications* **9**, 2460, doi:10.1038/s41467-018-04872-8 (2018).

47 Rathore, A. *et al.* The Local Dinucleotide Preference of APOBEC3G Can Be Altered from 5′-CC to 5′-TC by a Single Amino Acid Substitution. *Journal of Molecular Biology* **425**, 4442-4454, doi:https://doi.org/10.1016/j.jmb.2013.07.040 (2013).

48 Bacman, S. R. *et al.* MitoTALEN reduces mutant mtDNA load and restores tRNA(Ala) levels in a mouse model of heteroplasmic mtDNA mutation. *Nat Med* **24**, 1696-1700, doi:10.1038/s41591-018-0166-8 (2018).
