# Supplementary material for: CRISPR-free base editors with enhanced activity and expanded targeting scope in mitochondrial and nuclear DNA
Source: Nat Biotechnol. 2022 Apr 4;40(9):1378–87. doi: 10.1038/s41587-022-01256-8 (PMC9463067; doi:10.1038/s41587-022-01256-8)
Supplement: Supplementary file 2 — Reporting Summary [file 41587_2022_1256_MOESM2_ESM.pdf]

nature research

Corresponding author(s): David R. Liu

Last updated by author(s): 02/05/2022

Reporting Summary

Nature Research wishes to improve the reproducibility of the work that we publish. This form provides structure for consistency and transparency in reporting. For further information on Nature Research policies, see Authors & Referees and the Editorial Policy Checklist.

Statistics

For all statistical analyses, confirm that the following items are present in the figure legend, table legend, main text, or Methods section.

n/a	Confirmed	
☐	☒	The exact sample size (*n*) for each experimental group/condition, given as a discrete number and unit of measurement
☐	☒	A statement on whether measurements were taken from distinct samples or whether the same sample was measured repeatedly
☐	☒	The statistical test(s) used AND whether they are one- or two-sided<br>*Only common tests should be described solely by name; describe more complex techniques in the Methods section.*
☒	☐	A description of all covariates tested
☒	☐	A description of any assumptions or corrections, such as tests of normality and adjustment for multiple comparisons
☐	☒	A full description of the statistical parameters including central tendency (e.g. means) or other basic estimates (e.g. regression coefficient) AND variation (e.g. standard deviation) or associated estimates of uncertainty (e.g. confidence intervals)
	☒	For null hypothesis testing, the test statistic (e.g. *F*, *t*, *r*) with confidence intervals, effect sizes, degrees of freedom and *P* value noted<br>*Give P values as exact values whenever suitable.*
☒	☐	For Bayesian analysis, information on the choice of priors and Markov chain Monte Carlo settings
☒	☐	For hierarchical and complex designs, identification of the appropriate level for tests and full reporting of outcomes
☒	☐	Estimates of effect sizes (e.g. Cohen's *d*, Pearson's *r*), indicating how they were calculated

Our web collection on statistics for biologists contains articles on many of the points above.

Software and code

Policy information about availability of computer code

Data collection	High-throughput sequencing data was collected on Illumina Miseq Control software (3.1) and Illumina Nextseq 550 instruments. Flow cytometry data was collected on Sony MA900 software. CRISPResso files were imported into Microsoft Excel (version 2201) for analyses
Data analysis	High-throughput sequencing data was analyzed using CRISPResso2 (v2.0.34). Sequencing reads were demultiplexed using the MiSeq Reporter (Illumina) and fastq files were analyzed using Crispresso2. Prism 8 (GraphPad) was used to generate dot plots and bar plots of these data. For deumultiplexing Nextseq reads, bcl2fastq (v2.20) was used. For analysis of NCN target sequences, grep from the SeqKit v0.16.1 -package was used. Further details and references are provided in the Methods. For mitochondrial genome analysis, genome mapping was performed with BWA (v0.7.17) using NC_012920 genome as a reference. Duplicates were marked using Picard tools (v2.20.7). Pileup data from alignments were generated with SAMtools (vl.9) and variant calling was performed with VarScan2 (v2.4.3). Variants that were present at a frequency greater than 0.1% and a p-value less than 0.05 (Fisher's Exact Test) were called as high-confidence SNPs independently in each biological replicate. Only reads with Q> 30 at a given position were taken into account when calling SNPs at that particular position. Further details and references are provided in the Methods.

For manuscripts utilizing custom algorithms or software that are central to the research but not yet described in published literature, software must be made available to editors/reviewers. We strongly encourage code deposition in a community repository (e.g. GitHub). See the Nature Research guidelines for submitting code & software for further information.

Data

Policy information about availability of data

All manuscripts must include a data availability statement. This statement should provide the following information, where applicable:

- Accession codes, unique identifiers, or web links for publicly available datasets
- A list of figures that have associated raw data
- A description of any restrictions on data availability

High-throughput sequencing and whole mitochondria sequencing data have been deposited in the NCBI Sequence Read Archive under accession code PRJNA753136. SNP identification for whole mitochondrial genome sequencing used the NC_012920 genome as a reference. Plasmids are available on Addgene. Amino acids sequences of all base editors in this study are provided in the Supplementary Information as Supplementary Sequences 1–3. TALE sequences for SIRT6-DdCBE and JAK2-DdCBE are from Addgene plasmids #TAL2406, TAL2407, TAL2454 and TAL24

Field-specific reporting

Please select the one below that is the best fit for your research. If you are not sure, read the appropriate sections before making your selection.

☒ Life sciences ☐ Behavioural & social sciences ☐ Ecological, evolutionary & environmental sciences

For a reference copy of the document with all sections, see nature.com/documents/nr-reporting-summary-flat.pdf

Life sciences study design

All studies must disclose on these points even when the disclosure is negative.

Sample size	Sample sizes were n=3 independent biological replicates, which we and others have found to be sufficient in mammalian cell gene editing experiments to yield reproducible mean result values.
Data exclusions	No data was excluded.
Replication	Biological triplicate experiments were done with distinct aliquots of cells at intervals ranging from weeks to months between experiments. All experiments were repeated at least once. All attempts were successful.
Randomization	All independent biological replicates were treated identically and all data from all replicates was included. Thus randomization was not relevant to this study.
Blinding	Mammalian cells used in this study were grown under identical conditions; blinding was not used.

Reporting for specific materials, systems and methods

We require information from authors about some types of materials, experimental systems and methods used in many studies. Here, indicate whether each material, system or method listed is relevant to your study. If you are not sure if a list item applies to your research, read the appropriate section before selecting a response.

Materials & experimental systems

n/a	Involved in the study	
☒	☐	Antibodies
☐	☒	Eukaryotic cell lines
☒	☐	Palaeontology
☒	☐	Animals and other organisms
☒	☐	Human research participants
☒	☐	Clinical data

Methods

n/a	Involved in the study	
☒	☐	ChIP-seq
☐	☒	Flow cytometry
☒	☐	MRI-based neuroimaging

Eukaryotic cell lines

Policy information about cell lines

Cell line source(s)	HEK293T (ATCC CRL-3216), U2OS (ATTC HTB-96), K562 (ATCC CCL-243) and HeLa (ATCC CCL-2)
Authentication	Cells were authenticated by the supplier using STR analysis.
Mycoplasma contamination	All cell lines tested negative for mycoplasma.
Commonly misidentified lines (See ICLAC register)	None used.

Flow Cytometry

Plots

Confirm that:

☒ The axis labels state the marker and fluorochrome used (e.g. CD4-FITC).

☒ The axis scales are clearly visible. Include numbers along axes only for bottom left plot of group (a 'group' is an analysis of identical markers).

☒ All plots are contour plots with outliers or pseudocolor plots.

☒ A numerical value for number of cells or percentage (with statistics) is provided.

Methodology

Sample preparation	Cells were grown in tissue culture and then transfected as described in the Methods. 3 days following transfection, cells were trypsinized, resuspended in 1xPBS, and then filtered to remove debris.
Instrument	LE-MA900 cell sorter (Sony)
Software	MA900 software
Cell population abundance	The abundances of GFP+/mCherry+ cells were 1.1%, 2.71%, 31.5% and 60% for HeLa, K562, U2OS and HEK293T cells, respectively.
Gating strategy	Negative control cells not transfected with GFP plasmid and mCherry plasmid were used to establish GFP-/mCherry- gates. Positive control cells transfected with GFP plasmid and mCherry plasmid was used to determine the gate for GFP+/mCherry+ cells. This gate was used for all subsequent samples, such that all samples were gated based on the same fluorescence intensity. Please see Supplementary Note 2 for more information.

☒ Tick this box to confirm that a figure exemplifying the gating strategy is provided in the Supplementary Information.
